# Cooperative and distinct functions of MK2 and MK3 in the regulation of the macrophage transcriptional response to lipopolysaccharide

**DOI:** 10.1038/s41598-019-46791-8

**Published:** 2019-07-30

**Authors:** Christian Ehlting, Julia Rex, Ute Albrecht, René Deenen, Christopher Tiedje, Karl Köhrer, Oliver Sawodny, Matthias Gaestel, Dieter Häussinger, Johannes Georg Bode

**Affiliations:** 1 Clinic for Gastroenterology, Hepatology and Infectiology, University Hospital, Medical Faculty, Heinrich Heine University of Düsseldorf, Düsseldorf, Germany; 2 Institute for System Dynamics, University of Stuttgart, Stuttgart, Germany; 3 Biological and Medical Research Center (BMFZ), Genomics & Transcriptomics Laboratory, Heinrich Heine University of Düsseldorf, Düsseldorf, Germany; 4 Institute of Cell Biochemistry, Hannover Medical School, Hannover, Germany; 5 Department of Cellular and Molecular Medicine, University of Copenhagen, Copenhagen, Denmark

**Keywords:** Monocytes and macrophages, Toll-like receptors

## Abstract

The p38^MAPK^ downstream targets MAPKAP kinases (MK) 2 and 3 are critical for the regulation of the macrophage response to LPS. The extents to which these two kinases act cooperatively and distinctly in regulating LPS-induced inflammatory cytokine expression are still unclear. To address this uncertainty, whole transcriptome analyses were performed using bone marrow-derived macrophages (BMDM) generated from MK2^−/−^ or MK2/3^−/−^ animals and their wild-type littermates. The results suggest that in BMDM, MK2 and MK3 not only cooperatively regulate the transcript expression of signaling intermediates, including IL-10, IL-19, CXCL2 and the IL-4 receptor (IL-4R)α subunit, they also exert distinct regulatory effects on the expression of specific transcripts. Based on the differential regulation of gene expression by MK2 and MK3, at least six regulatory patterns were identified. Importantly, we confirmed our previous finding, which showed that in the absence of MK2, MK3 negatively regulates IFN-β. Moreover, this genome-wide analysis identified the regulation of Cr1A, NOD1 and Serpina3f as similar to that of IFN-β. In the absence of MK2, MK3 also delayed the nuclear translocation of NFκB by delaying the ubiquitination and subsequent degradation of IκBβ, reflecting the substantial plasticity of the response of BMDM to LPS.

## Introduction

It is becoming increasingly evident that in addition to being effector cells of the immune system, macrophages have vital homeostatic functions ranging from rather janitorial tasks, such as the clearance of cellular debris or the maintenance of erythrocyte turnover, to critical functions in wound healing, regeneration and tissue repair. This broad spectrum of activities is ensured by the notable plasticity that enables the physiology of these cells to be altered in response to environmental cues, producing different cell populations with distinct functions. To this end, macrophages are tightly interconnected with the surrounding cell populations via the controlled release and permanent sensing of intercellular communication signals. These signals include signaling peptides such as cytokines, chemokines, growth factors or arachidonic acid derivatives. In macrophages, at least in response to inflammatory pathogens, the expression of many of these mediators is controlled by the MAP kinase family member p38^MAPK^ and its downstream effector molecules. In addition to being critical for the initiation and establishment of the inflammatory response, this pathway is part of a signaling network that—in a cell type-dependent manner—modifies intracellular signal transduction and the cellular response towards cytokines such as IL-6 according to the context of coacting signals^1–4^. In addition, the p38^MAPK^ pathway has been recently demonstrated to mediate not only the release of inflammatory mediators but also the type I interferon-dependent release of IL-10 and the subsequent prolonged activation of signal transducer and activator of transcription (STAT)3^2,5,6^. The latter protein is critical for the de-escalation and dissipation of the inflammatory response, indicating a key role of the p38^MAPK^ pathway in the orchestration of not only the induction but also the feedback control of the inflammatory response^2,7,8^.

An important part of the intracellular signal transduction elicited by p38^MAPK^ is mediated via the activation of the two downstream effector kinases of p38^MAPK^, MAPK-activated protein kinases (MAPKAPK or MK)2 and 3. These two kinases are highly critical for the control of the expression of a variety of intercellular communication signals, including various cytokines and chemokines such as TNF-α, IL-6, OSM, IFN-β, IL-10, CXCL2 and CXCL8. These two kinases are closely related isoenzymes and were initially demonstrated to cooperate in the regulation of inflammatory gene expression at the posttranscriptional level^9,10^. However, previous evidence indicates that in addition to their cooperative action, these kinases exhibit distinct regulatory roles in gene expression. Thus, at least in the context of the macrophage inflammatory response^5^, it has been demonstrated that MK3 exerts negative regulatory effects on lipopolysaccharide (LPS)-inducible gene expression in the absence of MK2. In particular, IκBβ protein levels and NFκB activation in response to LPS stimulation, as well as IRF3 expression, appear to be affected if only MK3 is present^5^. Therefore, it is unclear to what degree the different IκBβ levels observable in macrophages deficient in MK2 alone or both MK2 and MK3 are due to reduced expression or enhanced degradation. Moreover, it is unknown, if these distinct regulatory effects of MK2 and MK3 described for the regulation of the IFN-β gene are true for LPS-inducible expression of only IFN-β or also of other genes. The present study aims to further characterize the differential impact of MK2 and MK3 on LPS-induced gene expression in macrophages.

## Results

### Deletion of MK2 or deletion of both MK2 and MK3 strongly alters gene expression in response to LPS

**Figure 1:**
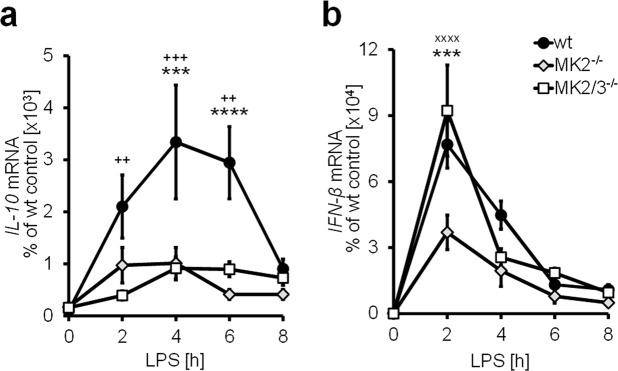
LPS-induced transcript expression of IL-10 and IFN-β is controlled by the interplay of MK2 and MK3. Bone marrow-derived macrophages (BMDM) were prepared from wild-type (wt), MK2-deficient (MK2^−/−^) and MK2/3-double-deficient (MK2/3^−/−^) mice and treated with 100 ng/ml LPS for the indicated durations. Total mRNA was extracted and analyzed using rtPCR. Data are presented as the means ± SEMs based on at least 5 replicates. Statistics were calculated by 2-way ANOVA with the Bonferroni test. A p-value of less than 0.05 was considered significant. The symbols indicate significance: *) wt vs. MK2^−/−^, ^+^) wt vs. MK2/3^−/−^ and ^x^) MK2^−/−^ vs. MK2/3^−/−^.

We previously demonstrated that in macrophages, the expression of the IL-10 gene in response to LPS requires MK2-mediated upregulation of IFN-β gene expression, activation of the IFN-α receptor (IFNAR) 1 and accumulation of the IL-10 transcript, which, in turn, is MK2/3-dependent^5^. Consistent with these findings, sustained IL-10 expression in response to LPS was significantly downregulated in primary bone marrow-derived macrophages (BMDM) that lacked MK2, an effect that - at least during early time points - tended to be enhanced upon the additional deletion of MK3 in MK2/3^−/−^ BMDM (Fig. 1a). In contrast to this pattern but consistent with previous data from our group^5^, the induction of IFN-β expression, which, in contrast to that of IL-10, occurs rapidly and is short-term, was impaired in MK2^−/−^ macrophages and restored upon the additional deletion of MK3 (MK2/3^−/−^; Fig. 1b) compared to that in wild-type control cells (wt BMDM). Notably, in macrophages that lack only MK3 (MK3^−/−^ BMDM), LPS-inducible expression of the IFN-β gene was not significantly affected (Supplemental Fig. S1b), suggesting that MK3 only exerts its effects on the expression of genes such as IFN-β in the absence of MK2.

There seems to be a complex interplay between MK2 and MK3, wherein MK2 neutralizes the negative regulatory effects of MK3 on LPS-induced IFN-β gene expression. However, it is not clear whether this effect is limited to IFN-β or is generally applicable to additional target genes. To answer this question, gene expression in response to LPS treatment was analyzed via whole genome array analyses in BMDM prepared from the bone marrow of MK2^−/−^ and MK2/3^−/−^ animals as well as from the corresponding control animals after 2 and 6 h of treatment with 100 ng/ml LPS.

**Figure 2:**
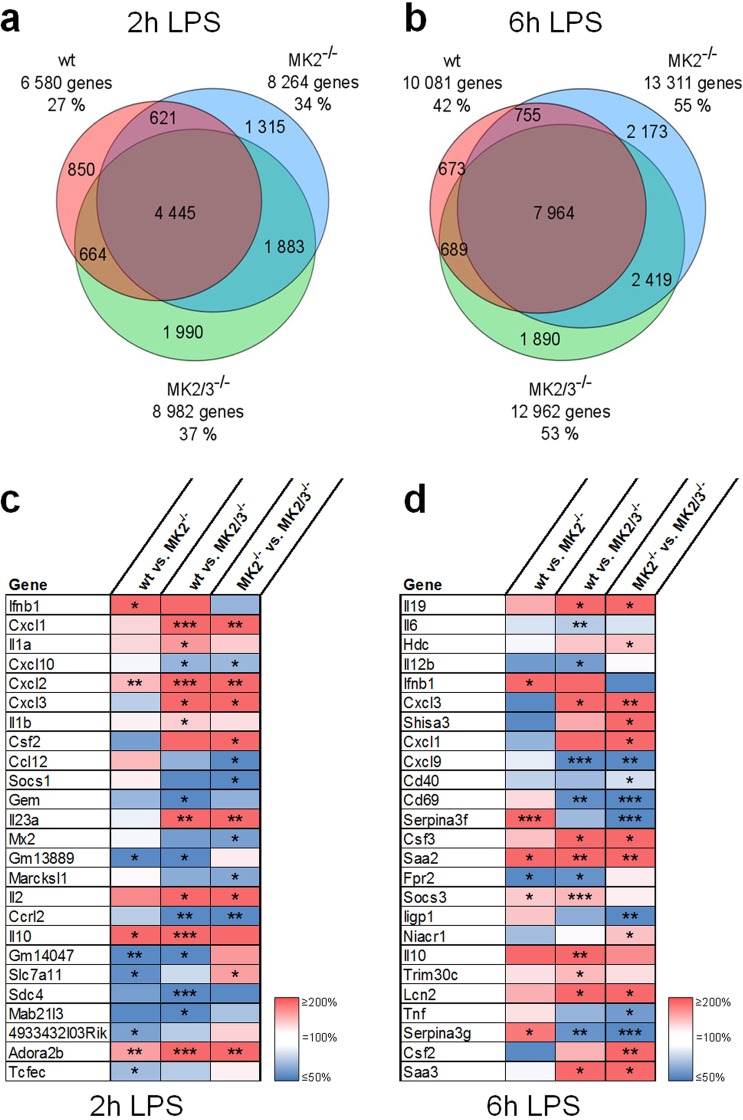
LPS-responsive genes controlled by MK2 and/or MK2/3: Whole genome array and the top 25 MK2/3-dependent genes with the strongest induction. For (**a,b**), BMDM were isolated from wild-type (wt), MK2-deficient (MK2^−/−^) and MK2/3-double-deficient (MK2/3^−/−^) mice and treated with 100 ng/ml LPS for 2 (**a**) or 6 (**b**) h. Total mRNA was extracted and subjected to microarray analysis to determine the total transcript levels. In total, 24,288 genes with known functions were analyzed and integrated into Venn diagrams when significantly up- or downregulated upon LPS treatment (t-test, p < 0.05). The numbers indicate the number of genes belonging to the indicated subset. The percentages reflect the ratio of the analyzed total genes. The red circles represent differentially regulated genes under wild-type conditions; blue, under MK2^−/−^ conditions; and green, under MK2/3^−/−^ conditions. From these genes, those with a minimum expression change of 4-fold from wild-type conditions were further analyzed after 2 (**c**) or 6 h (**d**) of LPS treatment. The top 25 differentially expressed genes with MK2- and/or MK2/3-dependency are listed. The three columns represent the relative LPS-induced expression level between the respective genotypes. wt vs. MK2^−/−^ indicates the level under wild-type conditions compared to that under MK2-deficient conditions. wt vs. MK2/3^−/−^ indicates the level under wild-type conditions compared to that under MK2/3 double-deficient conditions. MK2^−/−^ vs. MK2/3^−/−^ indicates the level under MK2-deficient conditions compared to that under MK2/3 double-deficient conditions. Therefore, the heat map color for each gene in each column reflects the degree of the difference between the expression levels of the respective genotypes. The red fields show enhanced expression in macrophages from the first listed genotype compared with the expression in the second genotype, whereas the blue fields show decreased expression in the first listed genotype compared with that in the second listed genotype. The white fields indicate no difference between the listed genotypes. Color transitions indicate patterns of differences in which at least one of the compared expression levels was significantly different (*p < 0.05; **p < 0.01; ***p < 0.001).

In this approach, the analysis was restricted to 33,325 out of 55,819 probes referring to genes related to NCBI listed accession numbers. Since several genes were proven by transcript expression with multiple probes, a total of 24,288 genes were included in the analysis. A response to LPS was considered relevant when the expression of the respective gene was significantly up- or downregulated, with a p-value of at least 0.05, upon treatment of the cells with LPS. Accordingly, after 2 h, a total of 11,768 genes and after 6 h, a total of 16,563 genes were significantly up- or downregulated independent of the genotype. Next, these genes were separated by genotype, and the results are presented in Venn diagrams (Fig. 2a,b). In wild-type macrophages, 6,580 transcripts (27% of the analyzed 24,288) exhibited a change in their expression after 2 h of treatment with 100 ng/ml LPS (Fig. 2a), while in MK2^−/−^ and MK2/3^−/−^ macrophages, 8,264 (34%) and 8,982 (37%) of the genes, respectively, were influenced in response to LPS treatment. The overall number of genes responsive to LPS increased to 10,081 (42%) in wt BMDM and to 13,311 (55%) or 12,962 (53%) in MK2^−/−^ or MK2/3^−/−^ BMDM, respectively (Fig. 2b), when stimulation with LPS was extended to 6 h.

Notably, after 120 min or 360 min of treatment, a group of 5,188 or 6,482 genes, respectively, became LPS-responsive, but this effect occurred only if either MK2 or both MK2 and MK3 were deleted. Hence, these data indicate that, in addition to being critical for the regulation of gene expression in macrophages in response to LPS, MK2 and/or MK3 are required to prevent a large number of genes from being regulated by LPS or from contributing to feedback regulation of the LPS response (Fig. 2a,b).

### Analysis of the differential regulation of LPS-inducible gene expression in macrophages by MK2 and/or MK3 reveals six different modes of regulation

Next, we aimed to focus on the LPS-responsive genes that were upregulated in wt BMDM and differentially regulated in the absence of either MK2 alone and/or both MK2 and MK3. Thus, the number of transcripts analyzed was restricted to those that were significantly upregulated in response to LPS in wt BMDM by at least four-fold. Of the 24,288 genes analyzed after 120 min of LPS treatment, 682 were identified to meet these criteria, and this number increased to 1,065 genes after 360 min of stimulation with LPS. From these genes, identified as LPS-responsive, the expression level within each genotype (wt, MK2^−/−^, MK2/3^−/−^) was compared with the respective other two genotypes (wt vs. MK2^−/−^, wt vs. MK2/3^−/−^, MK2^−/−^ vs. MK2/3^−/−^). As a result 131 (120 min LPS incubation time) or 478 (360 min LPS) of the LPS-responsive genes were at least for one condition (wt vs. MK2^−/−^ or wt vs. MK2/3^−/−^ or MK2^−/−^ vs. MK2/3^−/−^) significantly changed and therefore were defined as MK2- and/or MK2/3-dependently regulated. The LPS-responsive genes determined by this procedure and considered to being MK2- and/or MK2/3-dependently regulated in BMDM are listed according to their relative expression level in the Supplemental Material (Supplemental Fig. S2a,b, p-values are listed in S2c,d). The top 25 of these genes are summarized as heat maps in Fig. 2c,d with *Ifnb1* (gene symbol of IFN-β) as the factor, which is most prominently upregulated by LPS after 2 h (Fig. 2c) and *Il19* (IL-19) as the gene with most prominent upregulation of gene expression after 6 h of LPS treatment (Fig. 2d). Compared to wt BMDM the abundance of both transcripts was significantly diminished upon deletion of MK2 (wt > MK2^−/−^, Fig. 2c,d first column) and in the case of IL-19 transcript expression was also significantly reduced in MK2/3^−/−^ BMDM (*Il19*, Fig. 2d second column, wt > MK2/3^−/−^) as suggested by the red color in the heat maps (wt > MK2^−/−^ or wt > MK2/3^−/−^). This indicates that in BMDM MK2 plays a critical role for the upregulation of these two genes in response to LPS. Contrariwise, when compared to MK2^−/−^ BMDM, additional deletion of MK3 in MK2/3^−/−^ BMDM results in an upregulation of the *Ifnb1* transcript as indicated by the blue color (MK2^−/−^ < MK2/3^−/−^). Although in the analysis of the array data this upregulation was not significant, it was consistent with the RT-qPCR data outlined above (Fig. 1b) indicating that LPS-inducible transcript expression of the IFN-β gene is strongly diminished upon deletion of MK2 in MK2^−/−^ BMDM, whereas, compared to MK2^−/−^ BMDM, IFN-β gene expression significantly increases again upon additional deletion of MK3 in MK2/3^−/−^ BMDM and time dependently even exceeds that observed in wt BMDM. In addition, the expression levels of these genes compared to the wild-type control are listed in the Supplemental Fig. S2e1,f1 with the wild-type control defined as 100% and those genes mentioned in the following text are also presented as bar charts (Supplemental Fig. S2g–l).

**Figure 3:**
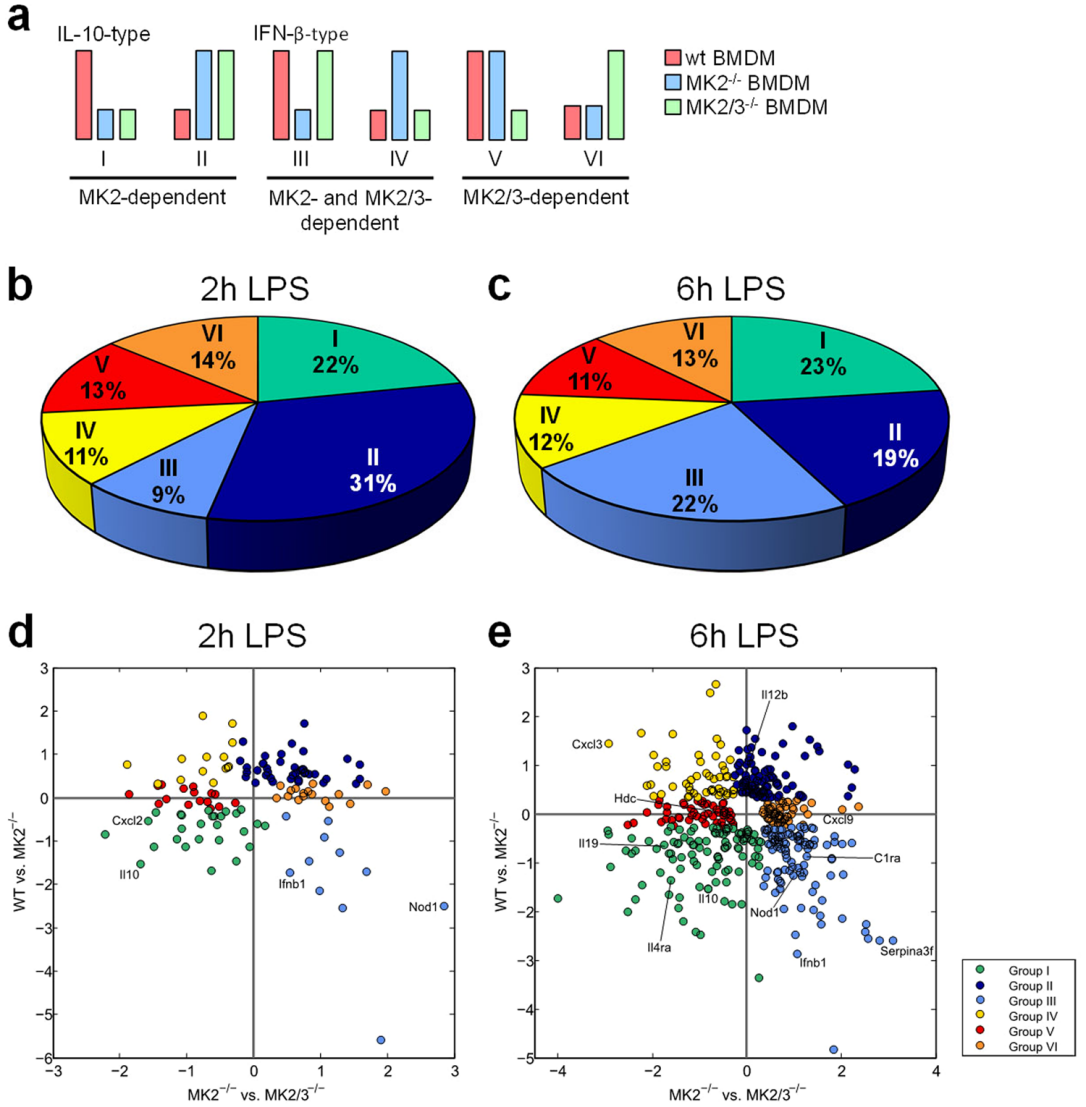
Classification and allocation of the MK2/3-dependent genes by 6 different patterns of regulation and distribution of MK2- and MK2/3-regulated genes according to their expression pattern. The proposed 6 different patterns of regulation dependent on MK2 and MK3 are shown in (**a**) (groups I-VI). The long bars indicate high induction and the short bars indicate low induction of LPS-responsive genes. The red bars reflect the condition in wild-type macrophages, the blue bars reflect the MK2^−/−^ condition, and the green bars reflect the MK2/3^−/−^ condition. According to their expression patterns, all the analyzed genes listed in Supplemental Fig. S2, of which the top 25 are listed in the heat maps in Fig. 2 (**c**,**d**), were allocated to the 6 distinct groups and are shown in pie charts for the 2 h (**b**) and 6 h (**c**) treatments with 100 ng/ml LPS. The proportion of genes in each group is depicted in percentages. For the allocation, a minimum difference of 20% in LPS-induced gene expression between the genotypes was considered relevant (Supplemental Fig. S3). Furthermore, the distinct genes summarized in these pie charts are shown in the scatter plot according to their relative expression value after 2 h (**d**) and 6 h (**e**) of treatment with 100 ng/ml LPS. The log-scaled x- and y-axis of the scatter plots indicates the relative expression level between the depicted genotypes with 0 meaning no difference, negative values meaning lower expression in MK2^−/−^ than in wt BMDM (wt vs. MK2^−/−^ on the y-axis) or in MK2/3^−/−^ than in MK2^−/−^ BMDM (MK2^−/−^ vs. MK2/3^−/−^ on the x-axis) and positive values meaning higher expression in MK2^−/−^ than in wt BMDM (wt vs. MK2^−/−^ on the y-axis) or in MK2/3^−/−^ than in MK2^−/−^ BMDM (MK2^−/−^ vs. MK2/3^−/−^ on the x-axis). Target genes that were analyzed further, as shown in Fig. 4, are depicted (**d,e**). In b to e, each group is identified by its distinct color, as shown in the figure legend.

To identify genes that are differentially regulated regarding wt vs. MK2^−/−^, wt vs. MK2/3^−/−^ or MK2^−/−^ vs. MK2/3^−/−^ a variation of the mean values between the respective genotypes of >20% (wt vs. MK2^−/−^ or wt vs. MK2/3^−/−^ or MK2^−/−^ vs. MK2/3^−/−^: >or <20%) was set as a threshold. Using this threshold, the heat maps further suggested that the regulatory pattern (wt > MK2^−/−^, MK2/3^−/−^ > MK2^−/−^) observed for *Ifnb1* is not only the case for *Ifnb1*, but also for other genes including genes like *Ccl12*, *Iigp1*, *Serpina3f* and *Serpina3g* (Supplemental Fig. S2g). The regulatory pattern suggested for this group of genes is summarized as group III in Fig. 3a. Contrariwise, in case of genes such as *Adora2b*, *Cxcl2*, *Il2*, *Il10*, *Lcn2* or *Saa2* the analysis reflected a different regulatory pattern (wt > MK2^−/−^, MK2/3^−/−^ < or = MK2^−/−^) similar to that seen for *Il19* (Supplemental Fig. S2h). These genes are summarized as group I in Fig. 3a. As it was the case for the *Ifnb1* gene, the regulatory pattern identified for the regulation of the *Il10* gene was in line with the RT-qPCR data outlined above (Fig. 1a) and with previous observations from our group^5^.

Interestingly, apart from these two groups the heat maps in Fig. 2c,d suggest additional groups with distinct regulatory patterns of LPS-induced gene expression. Thus, the genes *Cd40*, *Gem*, *Fpr2*, *Il12b*, *Mab21l3*, *Sdc4* and *Tcfec* are enhanced in the absence of either MK2 alone (MK2^−/−^) or of both MK2 and MK3 (MK2/3^−/−^) (Supplemental Fig. S2i), indicating that MK2 and/or MK2/3 restrict the LPS-inducible expression of these genes to lower levels (MK2^−/−^ > WT, MK2/3^−/−^ > WT; summarized in group II). In addition, another group of genes comprising *Cxcl3*, *Csf2*, *Niacr1*, *Slc7a1* and *Shisa3* appears to be MK3-dependently amplified in the absence of MK2 (summarized in group IV) since the enhancement of LPS-inducible gene expression is observable in MK2^−/−^ BMDM but does not occur upon combined deletion of MK2 and MK3 in MK2/3^−/−^ BMDM (MK2^−/−^ > WT, MK2/3^−/−^ < or = WT) (Supplemental Fig. S2j). Furthermore, in some cases gene expression is only up- or down-regulated when both MK2 and MK3 are deleted, whereas deletion of MK2 alone appears to have no effect on gene expression. Thus, when compared to wt or MK2^−/−^ BMDM the expression of the genes *Hdc*, *Il23a* or *Saa3* (summarized in group V) is diminished upon combined deletion of MK2 and MK3 (Supplemental Fig. S2k), while the genes *Cd69*, *Cxcl9*, *Cxcl10*, *Marcksl1*, *Mx2* or *Socs1* are upregulated in the absence of both MK2 and MK3 in MK2/3^−/−^ BMDM (summarized in group VI) (Supplemental Fig. S2l).

Based on these six groups defined on the basis of the distinct regulation of LPS-inducible gene expression by the protein kinases MK2 and MK3 (Figs 2c,d and S2a–d) the genes identified to be MK2- and/or MK2/3-dependently regulated by LPS were allocated to one of the six defined groups (Fig. 3a). Thereby, as outlined above, a gene was considered to be differentially regulated regarding wt vs. MK2^−/−^, wt vs. MK2/3^−/−^ or MK2^−/−^ vs. MK2/3^−/−^ respectively, when the mean values between the respective genotypes varied at least 20%, which was set as a threshold (wt vs. MK2^−/−^ or wt vs. MK2/3^−/−^ or MK2^−/−^ vs. MK2/3^−/−^: >or <20%). Using this approach the heatmap could be transformed into a map without any changes in the transition of color and assign a clear position for each gene within one of the six expression patterns (Supplemental Fig. S3a,b). Based on this procedure, 31% of the 131 genes that were MK2- and/or MK2/3-dependently regulated after 2 h of LPS treatment, could be allocated to group II (Figs 3b and S4a). Correspondingly, after 6 h of LPS treatment, 478 genes could be allocated to the six different expression patterns, with gene expression groups I and III as the biggest groups − 23% and 22%, respectively (Figs 3c and S4b). Moreover, the proportion of LPS-inducible genes regulated by the interplay of MK2 and MK3 in the same way as *Ifnb1* increased from 9% after 2 h to 22% after 6 h of LPS treatment.

**Figure 4:**
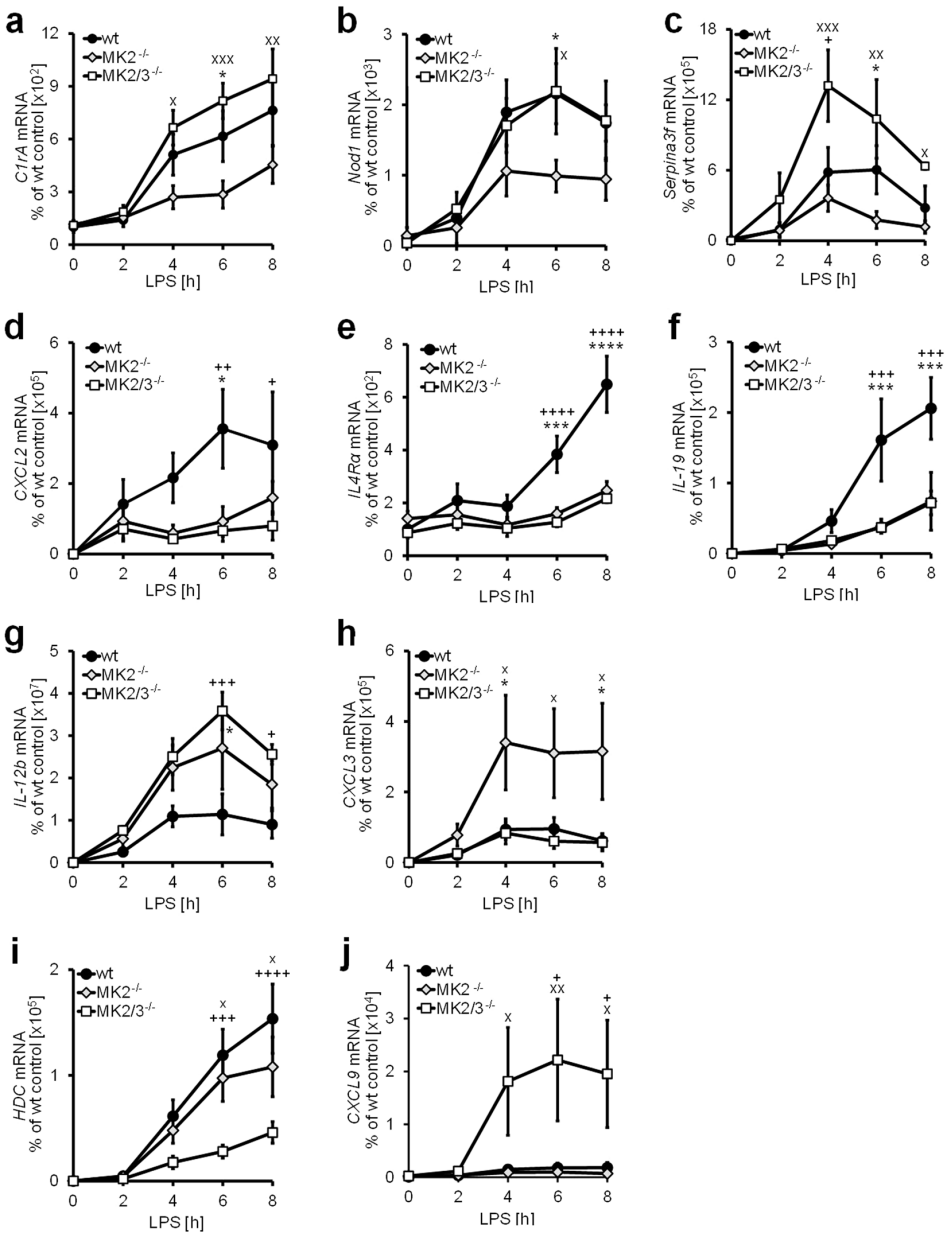
LPS-induced and MK2- and/or MK2/3-dependent expression of target genes and representatives of the 6 different types of regulation. Bone marrow-derived macrophages (BMDM) were prepared from wild-type (wt), MK2-deficient (MK2^−/−^) and MK2/3-double-deficient (MK2/3^−/−^) mice and treated with 100 ng/ml LPS for the indicated time points. Total mRNA was extracted and analyzed using rtPCR as specified in the methods section. C1rA, Nod1 and Serpina3f are representative target genes of group III (**a–c**), whereas CXCL2, IL-4Rα and IL-19 are representatives of group I (**d–f**). Group II is represented by IL-12b (**g**), group IV by CXCL3 (**h**), group V by HDC (**i**) and group VI by CXCL9 (**j**). Data are presented as the means ± SEMs based on at least 3 replicates per condition. Statistics were calculated by 2-way ANOVA with the Bonferroni test. A p-value of less than 0.05 was considered significant. The following symbols indicate significance: *) wt vs. MK2^−/−^, ^+^) wt vs. MK2/3^−/−^ and ^x^) MK2^−/−^ vs. MK2/3^−/−^.

To demonstrate the relative strength of the influence of the respective knockout phenotype compared to the other genotypes on LPS-induced expression of the respective transcripts allocated to the six different groups in Fig. 3b,c, we further constructed a two-dimensional scatter plot (Fig. 3d,e). Based on these analyses, genes representative of one of the six defined groups were selected to further validate and corroborate the different regulatory patterns characterizing the six different groups. As the original aim of the study was primarily to test the hypothesis that genes other than *Ifnb1* are also similarly regulated by the interplay of MK2 and MK3, three genes - complement component 1 r subcomponent A (*Cr1a*), the inflammasome-component nucleotide-binding oligomerization domain-containing protein 1 (*Nod1*) and the serine peptidase inhibitor clade A member 3 F (Serpina3f) - were selected to further validate the regulatory pattern characteristic of group III. As demonstrated in Fig. 4a–c, LPS-inducible transcription of these three genes was reduced upon deletion of MK2 and, as is the case for IFN-β (Fig. 1b), the additional deletion of MK3 in MK2/3^−/−^ BMDM resulted in at least partial reconstitution of the transcript levels compared to those in wt BMDM. Similarly, the chemokine CXCL2 (*Cxcl2*), the IL4 receptor (IL4R)α (*Il4ra*) and IL-19 (*Il19*) (Fig. 4d–f) were chosen as three genes that are regulated in a similar way as IL-10 (*Il10*) (Fig. 1a) and belong to the same group of genes (group I), which has been previously described as being either mainly MK2-dependently regulated or cooperatively regulated by MK2 and MK3. Additionally, based on the results shown in Fig. 3 for each of the other groups (II, IV, V and VI) defined in the present study, a representative gene was chosen, and its LPS-inducible expression was analyzed in wt BMDM, MK2^−/−^ BMDM and MK2/3^−/−^ BMDM using RT-qPCR for further validation. Consistent with the regulatory pattern described above for the genes summarized as group II, the LPS-induced accumulation of IL-12b transcripts was enhanced in MK2^−/−^ as well as in MK2/3^−/−^ BMDM compared to that in wt BMDM (Fig. 4g), corroborating the assumption that the LPS-inducible expression of these genes is limited by either MK2 alone or by MK2 and MK3 in cooperation. In contrast, the transcript level of the chemokine CXCL3 (*Cxcl3*) was enhanced in MK2^−/−^ BMDM but not in MK2/3^−/−^ BMDM compared to that in wt BMDM (Fig. 4h). Hence, as this gene is a member of group IV, its LPS-induced expression in BMDM lacking MK2 appears to be upregulated by MK3. In contrast, the transcript levels of genes such as HDC (*Hdc*) (group V) and CXCL9 (*Cxcl9*) (group VI) were either reduced (group V) or enhanced (group VI) in BMDM lacking both MK2 and MK3 (MK2/3^−/−^ BMDM) but not in wt BMDM or in MK2^−/−^ BMDM (Fig. 4i,j).

Notably, the LPS-inducible expression of none of the genes investigated in Fig. 1 (IL-10 and IFN-β) and Fig. 4 (Nod1, Cr1A, Serpina3f, CXCL2, IL-4Rα, IL-19, IL-12b, CXCL3, HDC and CXCL9) was significantly impaired in BMDM in which only MK3 was absent (Supplemental Figs S1 and S5). Hence, these data indicate that in the presence of MK2, the function of MK3 may be replaceable or compensated by another, yet unidentified, molecule or process.

### As demonstrated recently for genes such as IL-10, tristetraprolin plays an important role in the regulation of genes summarized in group I

**Figure 5:**
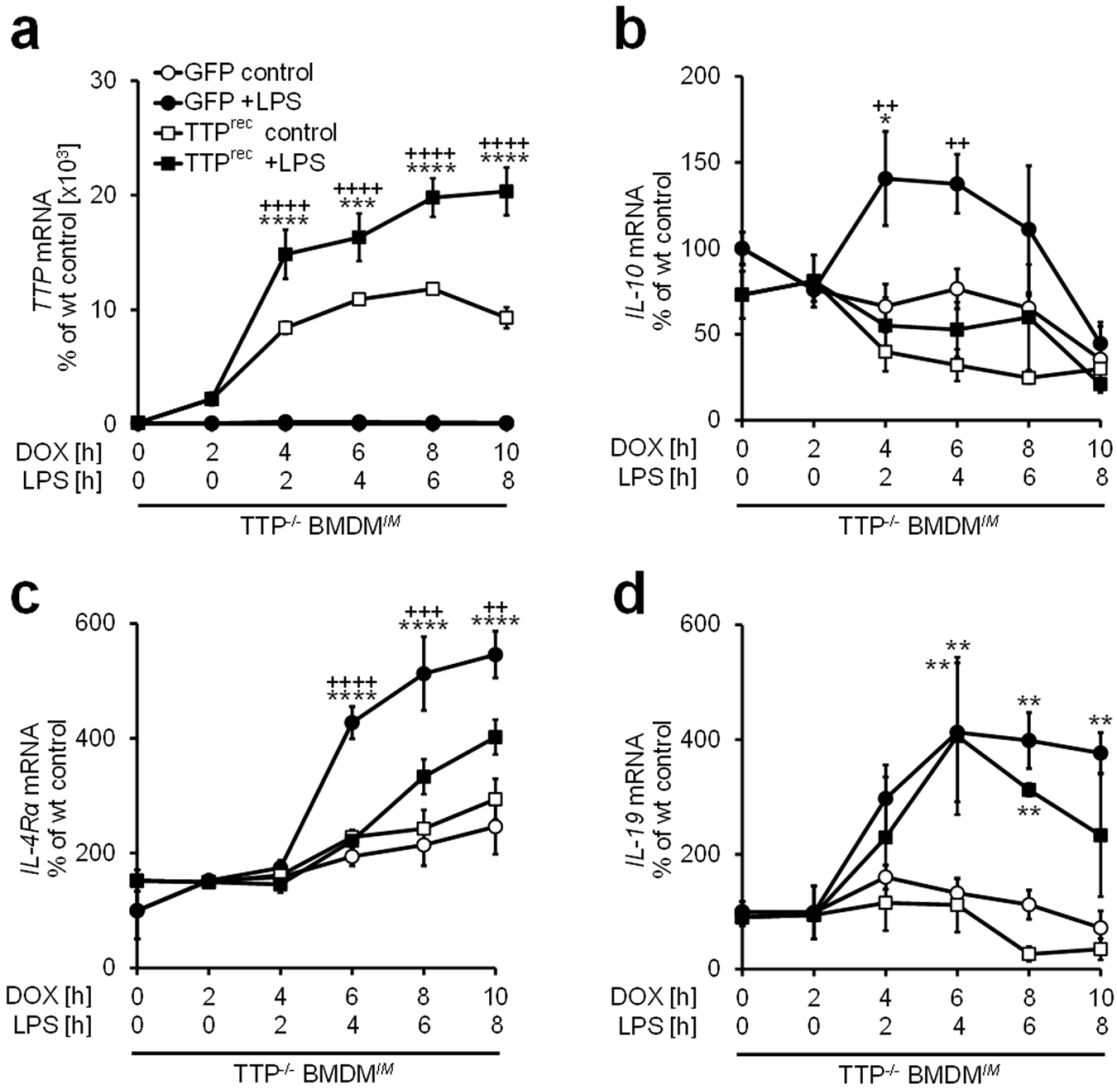
The mRNA binding protein tristetraprolin (TTP) controls the transcript expression of the genes summarized in group I. Immortalized TTP^−/−^ bone marrow-derived macrophages (TTP^−/−^ BMDM^*IM*^) stably expressing a plasmid allowing tetracycline-inducible reconstitution of tristetraprolin (TTP^rec^) expression or GFP expression as the control were used. Cells were treated with 4 µg/ml doxycycline (DOX) for 2 h and were then stimulated with 100 ng/ml LPS for the indicated durations. The expression of TTP (**a**), IL-10 (**b**), IL-4Rα (**c**) or IL-19 (**d**) was analyzed using rtPCR. Data are presented as the means ± SEMs based on at least 3 replicates per condition. Statistics were calculated by 2-way ANOVA with the Bonferroni test. A p-value of less than 0.05 was considered significant, and the following symbols indicate significance: *) control vs. LPS and ^+^) GFP vs. TTP^rec^.

In addition to being regulated at the transcriptional level, the accumulation of IL-10^11–14^ and CXCL2^9,11^ transcripts is controlled at the level of mRNA stability. In this context, the mRNA binding factor tristetraprolin (TTP), which initiates mRNA degradation, is phosphorylated by MK2 and then disassociates from the target mRNA, leading to its stabilization^11,13,15–17^. Notably, the genes *Cxcl1*, *Cxcl2* and *Socs3* (Fig. 2c,d), as well as *Cebpd* and *Zfp36* (Supplemental Fig. S2), which were all defined as genes in group I (Supplemental Fig. S4), were also identified among the top 25 target genes of TTP reported in another array-based study^18^. Hence, it was conceivable that most of the group I genes are also targets of TTP, which was another common feature of the genes in this group. To investigate whether in addition to IL-10 and CXCL2, the IL-4Rα and IL-19 genes, which are representative members of group I, are also TTP target genes, we took advantage of immortalized TTP-deficient BMDM stably transfected with a vector encoding TTP or GFP under the control of a tetracycline-dependent promoter. In these cells, TTP expression can be reconstituted upon stimulation with doxycycline, allowing analysis of putative TTP target genes^19^. Notably, doxycycline-inducible TTP expression was further enhanced upon costimulation with LPS (Fig. 5a). Consistently and in agreement with previous data, LPS-inducible IL-10 expression was substantially impaired upon reconstitution of TTP expression (Fig. 5b). Similarly, upregulated transcript expression of the α subunit of the IL-4 receptor was significantly reduced in response to LPS, if TTP expression was coinduced (Fig. 5c), indicating that its transcript expression is also controlled by TTP. In contrast, the upregulation of IL-19 transcription in response to LPS was not affected by TTP at early time points, while the decrease in the transcript abundance over time appeared to be accelerated if TTP was present (Fig. 5d). Hence, at least at later time points, the IL-19 transcript may also belong to the group of TTP-regulated transcripts.

### Reconstitution of impaired NFκB activation in macrophages lacking MK2 upon the additional deletion of MK3 is due to accelerated ubiquitination and decay of IκBβ

We previously demonstrated that in MK2^−/−^ BMDM, nuclear translocation of the p65 NFκB subunit was impaired due to enhanced expression of inhibitor of κB (IκB)β and increased binding of p65 to IκBβ^5^. This impaired activation of NFκB in MK2^−/−^ BMDM has been demonstrated to be due to the action of MK3 in the absence of MK2. Since regular activation of NFκB is required for IFN-β gene expression, impaired NFκB activation was considered to be responsible for the reduced transcript expression observed in cells that lack MK2 (Fig. 1b).

**Figure 6:**
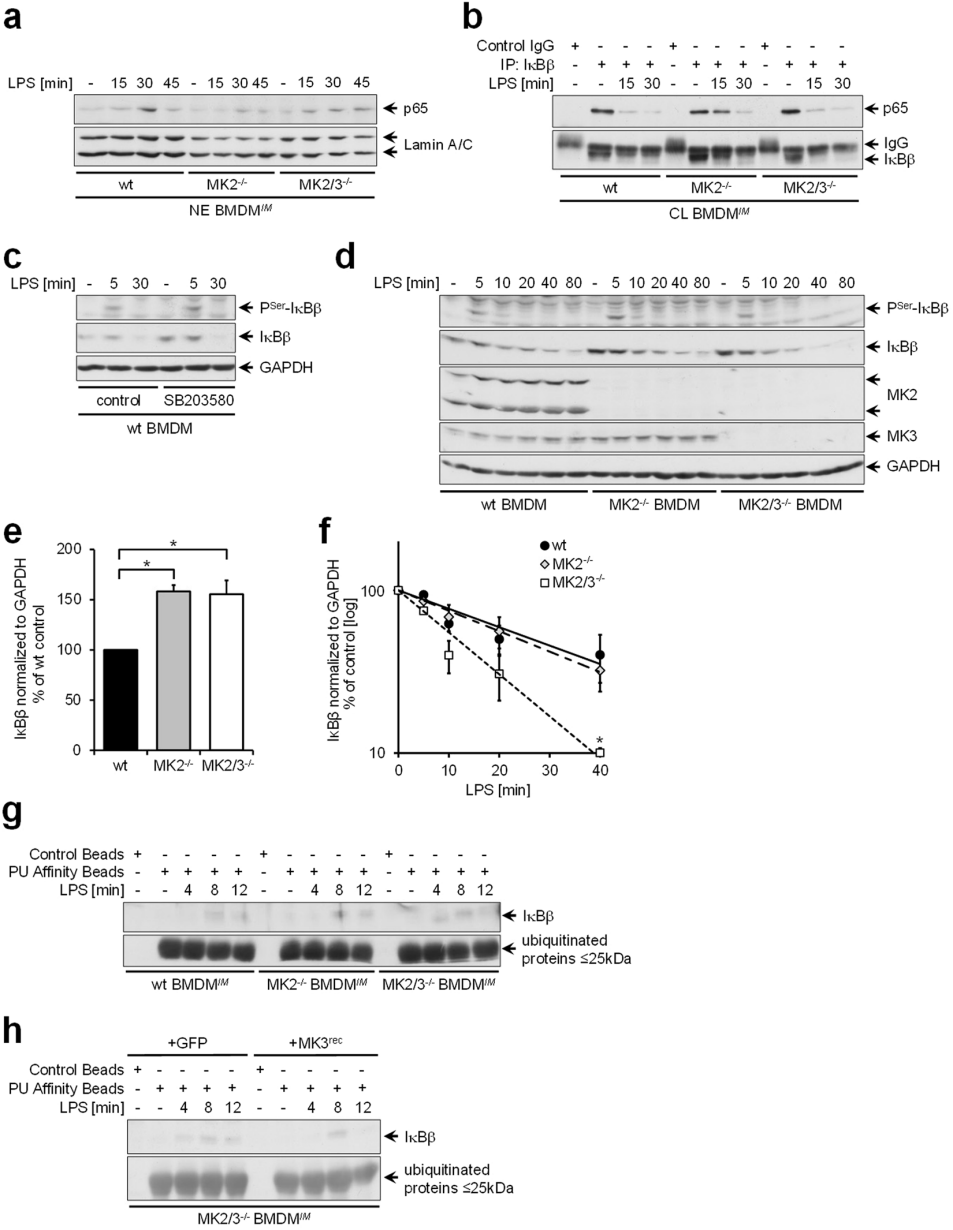
MK3 counteracts p65 NFκB nuclear translocation in the absence of MK2 by delaying the ubiquitination and subsequent degradation of the inhibitor of κB (IκB) β, whose protein accumulation is suppressed by MK2. For (**a,b**) and (**e–h**), immortalized bone marrow-derived macrophages (BMDM^*IM*^) were used. For (**a**), proteins in the nuclear extracts (NE) and for (**b**), proteins in the cytosolic lysates (CL) were prepared. For c and d, primary BMDM were prepared from wild-type (wt), MK2-deficient (MK2^−/−^) and MK2/3 double-deficient (MK2/3^−/−^) mice. For h, MK2/3^−/−^ BMDM^*IM*^ were stably transfected with a vector coexpressing MK3 or GFP as the control via an internal ribosomal reentry site (IRES) or with the same vector expressing only GFP as the control using retroviral gene transfer. Cells were treated with 100 ng/ml LPS for the indicated durations and (**c**), cells were treated with the p38^MAPK^ inhibitor SB203580 (5 µM) for 4 h prior to LPS treatment. Then, cells were harvested and further analyzed by immunoblotting using antibodies specifically recognizing the p65 subunit of the NFκB complex, producing a signal at a location corresponding to a molecular weight of 65 kDa according to the protein weight marker (**a,b**); nuclear full-length Lamin A/C, 70 kDa (**a**); IκBβ, 50 kDa (**d–h**); IκBβ phosphorylated at serine residues 19 and 23, 50 kDa (**c,d**); MK2, 40 and 50 kDa; MK3, 40 kDa (**d**); and GAPDH, 36 kDa (**c,d**). For (**b**), IκBβ and for (**g,h**), ubiquitin-ligated proteins were precipitated with polyubiquitin (PU) affinity beads and subjected to immunoblot analysis using antibodies specifically recognizing IκBβ, with a molecular weight of 50 kDa, or ubiquitinated proteins, from which the fraction containing proteins with a maximum molecular weight of 25 kDa is shown. (**a–d,g,h**) show samples from the same experiment, and the juxtaposed images show detections from different areas of the same blot or from the same area of different blots with the same samples, if the proteins of interest had approximately the same molecular weight. To set the focus on the individual antibody signals, images were cropped at the borders depicted with a black line. The time kinetics of IκBβ expression following LPS treatment shown in d were examined via densitometric analysis with n = 3 replicates, and data are presented as the means ± SEMs (**e,f**), demonstrating the basal expression (**e**) or time-dependent degradation of IκBβ (**f**). Statistics were calculated as described in the methods section; the following symbols indicate significance: *) wt vs. MK2^−/−^ or MK2/3^−/−^.

However, a more detailed analysis of the time dependency of NFκB subunit p65 appearance in nuclear extracts (Figs 6a and S6a) and its accompanying disappearance from cytosolic lysates (Figs 6b and S6b) in wt and MK2^−/−^ BMDM revealed that LPS-induced nuclear translocation of p65 is not completely abolished in MK2^−/−^ BMDM, but is diminished and delayed compared to that in wt BMDM. The additional deletion of MK3 in MK2/3^−/−^ BMDM contributed greatly to restoring the wild-type conditions with respect to the time course and extension of p65 nuclear translocation (Figs 6a,b and S6a,b). Notably, inhibition of p38^MAPK^ using the small molecule compound SB203580 (Fig. 6c) as well as deletion of MK2 (MK2^−/−^ BMDM) or both MK2 and MK3 (MK2/3^−/−^ BMDM) (Fig. 6d,e) resulted in an increased abundance of IκBβ molecules, consistent with previous observations from our group^5^. This increased availability of IκBβ molecules is considered to be responsible for the diminished and delayed release and nuclear translocation of the NFκB p65 subunit in MK2^−/−^ and in MK2/3^−/−^ BMDM compared to the wild-type conditions. Thereby, the kinetics of the decay of IκBβ as well as the time course of the phosphorylation of IκBβ at serines 19 and 23 upon stimulation with LPS did not significantly differ between wt and MK2^−/−^ BMDM (Fig. 6d,f). This pattern indicates that compared to wt BMDM, MK2^−/−^ BMDM exhibit increased IκBβ levels while the velocity of the decay of IκBβ remains equal to wild-type conditions. Interestingly, in accordance with the observation that in MK2/3^−/−^ BMDM, nuclear translocation of p65 in response to LPS treatment was almost restored to wild-type levels (Figs 6a and S6a), the velocity of LPS-triggered IκBβ decay was substantially enhanced upon the additional deletion of MK3 in MK2/3^−/−^ BMDM compared to that in wt and MK2^−/−^ BMDM, resulting in accelerated disappearance of IκBβ (Fig. 6d,f). It is therefore suggested that the restoration of the nuclear translocation of p65 to wild-type levels upon the additional deletion of MK3 in MK2/3^−/−^ BMDM is due to an enhancement in the decay of IκBβ molecules in these cells (Fig. 6d,f) that otherwise would bind p65^5^ and prevent its nuclear translocation. This enhancement was not accompanied by stronger or more prolonged Ser19/23 phosphorylation of IκBβ in MK2/3^−/−^ BMDM (Fig. 6d). In line with the increased velocity of IκBβ molecule decay, evidence is provided that compared to wild-type and MK2^−/−^ BMDM, MK2/3^−/−^ BMDM exhibit earlier ubiquitination of IκBβ (Figs 6g and S6c) and that this ubiquitination can be reduced to wild-type or MK2^−/−^ levels if MK3 is reintroduced (Figs 6h and S6d). Hence, in the absence of MK2, MK3 is responsible for delayed IκBβ ubiquitination. Our data suggest that the LPS-induced release of NFκB from IκBβ as well as the retention of NFκB in the cytosol by binding to IκBβ is controlled by the interplay of MK2 and MK3. In this case, MK2 suppresses the enhanced accumulation of IκBβ in the cytosol, whereas MK3 delays IκBβ ubiquitination and subsequent degradation to maintain temporally controlled LPS-induced NFκB release.

## Discussion

Previous reports demonstrated that MK2 and MK3 control the expression of several mediators of inflammation, such as TNF-α, CXCL2 and IL-10, in a cooperative manner^5,9^. Previous reports further suggested that MK2 and MK3 may also display distinct and, in part, contradictory regulatory effects on gene expression^5,20^. Hence, as outlined above in macrophages, MK2 upregulates the transcription of IFN-β by neutralizing the negative regulatory effects of MK3, which impedes nuclear translocation of NFκB in the absence of MK2^5^. Correspondingly, as shown previously^5^ and confirmed herein, the expression of IFN-β mRNA is lower in MK2^−/−^ BMDM than in wild-type BMDM, an effect that at least in part can be neutralized by the additional deletion of MK3, as seen in MK2/3^−/−^ BMDM (Fig. 1b). Notably, deletion of MK3 alone in MK3^−/−^ BMDM had no effect on the LPS-inducible expression of IFN-β, indicating that MK3 exerts its negative regulatory effects on IFN-β gene expression only in the absence of MK2 (Supplemental Fig. S1b). This behavior is in contrast to the LPS-inducible expression of IL-10, which was downregulated in MK2^−/−^ as well as in MK2/3^−/−^ BMDM (Fig. 1a) and tended to be reduced also in MK3^−/−^ BMDM (Supplemental Fig. S1a) compared with that in wild-type BMDM. This finding supports recent observations that the regulation of IL-10 expression is governed by MK2 in cooperation with MK3^5,9^.

A major aim of the present study was to clarify whether MK2 also controls the LPS-inducible expression of genes other than IFN-β by neutralizing the inhibitory effects of MK3 on gene expression. As outlined above, we defined a group of genes (group III in Figs 3 and S4) regulated by the interplay of MK2 and MK3 in a similar way as IFN-β that, among others, includes the acute phase protein and serine peptidase inhibitor (Serpin)a3f and the pathogen recognition receptor Nod1 as well as the serine protease and component of the complement system C1rA (Fig. 4a–c). As with the IFN-β gene (Fig. 1b), the LPS-inducible expression of these genes was significantly diminished in BMDM lacking MK2 compared to that in wild-type control BMDM but was reconstituted or even enhanced upon the additional deletion of MK3 (Fig. 4a–c). These data suggest that as it does for the IFN-β gene, MK3 negatively affects the expression of these genes in the absence of MK2, which neutralizes the negative regulatory effects of MK3. Of note, as it is the case for IFN-β gene expression, deletion of MK3 alone in MK3^−/−^ BMDM does not affect the LPS-inducible expression of these genes, indicating that the negative regulatory effect of MK3 only becomes apparent in the absence of MK2 (Supplemental Fig. S5a–c). Contrariwise, the LPS-inducible expression of genes summarized herein as group I (Fig. 3), including genes such as the chemokine CXCL2, the IL-4 receptor subunit (IL-4R)α and the cytokine IL-19 (Fig. 4d–f) is strongly impaired in MK2^−/−^ macrophages, an effect, which at least in part tends to be further enhanced upon additional deletion of MK3. Consistently, the data presented herein suggest that, although not significant, the LPS-inducible expression of these genes also tends to be impaired in the absence of MK3 (Supplemental Fig. S5d–f). This type of regulation corresponds to that noted in previous reports demonstrating that MK2 and MK3 cooperate in upregulating the biosynthesis of genes such as IL-10 and TNF-α^5,9^. Further studies as well as the results shown in Fig. 5 demonstrate that the transcripts of these genes are targets of the RNA-binding protein TTP^13–15,19,21^. These findings suggest that at least some of the genes in group I are also target genes of TTP.

Notably, the data presented herein suggest that in BMDM, the regulation of gene expression by the interplay of MK2 and MK3 in response to LPS is far more complex than the two regulatory patterns summarized as group I, or *Il10*-type, and group III, or *Ifnb1*-type. Hence, at least four additional regulatory patterns were identified based on the alteration of LPS-inducible gene expression in BMDM in response to deletion of MK2 or both MK2 and MK3 (Figs 3 and S4). These patterns suggest that in addition to cooperatively upregulating gene expression or neutralizing the negative regulatory effects of MK3, MK2 may either cooperate with MK3 to limit the upregulation of gene expression in response to LPS, as demonstrated for IL-12 subunit b (Fig. 4g, group II or *Il12b*-type), or counteract the enhancing effects of MK3, as demonstrated for CXCL3 (Fig. 4h, group IV or *Cxcl3*-type). In the latter case, gene expression was strongly enhanced in MK2^−/−^ BMDM compared with that in wild-type control cells, and this enhancement was reversed upon the additional deletion of MK3. In addition, as validated herein for the genes HDC (Fig. 4i) and CXCL9 (Fig. 4j), there are two groups - termed herein as groups V and VI (Figs 3 and S4) - in which gene expression was either diminished (group V or *Hdc*-type) or enhanced (group VI or *Cxcl9*-type) upon deletion of both MK2 and MK3, while deletion of MK2 alone did not significantly affect gene expression in response to LPS compared to that observed in wild-type BMDM. Notably, macropinocytosis of dendritic cells (DCs) in response to LPS treatment is diminished when both kinases are missing, whereas neither MK2 nor MK3 alone exert any influence on this particular function of DCs^22^. These observations indicate that in some cases, MK2 and MK3 may thoroughly compensate for each other, whereas deletion of both causes drastic effects by unbalancing the cellular response to LPS. The underlying molecular mechanisms of the complex interplay by which the two kinases MK2 and MK3 achieve these different mechanisms of regulation of gene expression in response to LPS is largely unclear and must be the subject of future research.

However, in this context, it is interesting to note that the negative regulatory effects of MK3 on IFN-β gene expression in the absence of MK2 are caused by delayed release of NFκB from its complex with IκBβ^5^. Consistent with this observation, compared to wild-type controls, MK2^−/−^ BMDM exhibited impaired LPS-inducible nuclear translocation of the NFκB complex p65 subunit (Figs 6a and S6a), coinciding with prolonged detectability of p65 in the cytoplasm (Figs 6b and S6b). As suggested by previous data from immortalized BMDM cell lines, this delayed activation of NFκB in MK2^−/−^ BMDM is due to increased expression of the IκBβ protein in the absence of MK2^5^. The latter observation was confirmed herein for primary BMDM, where IκBβ expression is also increased in cells lacking MK2 (Fig. 6d,e).

The data presented indicate that upon additional deletion of MK3 in MK2/3^−/−^ BMDM, NFκB activation is reconstituted in these cells, although the basal IκBβ levels are increased due to an accelerated decay of IκBβ (Fig. 6d,f). Thereby, as demonstrated herein for the first time, in the absence of MK2, MK3 delays ubiquitination and subsequent degradation of IκBβ. This delay is circumvented upon additional MK3 deletion in MK2/3^−/−^ BMDM. Consistently, reconstitution of MK3 expression in MK2/3^−/−^ BMDM resulted in delayed IκBβ ubiquitination, which substantiates the hypothesis that MK3 is indeed responsible for hindering the degradation of IκBβ upon MK2 deficiency (Figs 6h and S6d). Hence, we suggest that in BMDM lacking MK2 p65 translocation is delayed due to an increased abundance of IκBβ protein in the cytoplasm and a MK3-mediated delay of their ubiquitination resulting in a prolonged degradation of IκBβ. The molecular mechanism by which MK3 interferes with the ubiquitination and degradation of IκBβ in the absence of MK2 is unclear and remains to be established. However, in this context it may be interesting to note that previous work indicates that activation of MK2 is interlinked with regulation of ubiquitination. Hence, the endoplasmic reticulum-associated ubiquitin-conjugating enzyme Ube2j1 has been demonstrated to be a substrate of MK2 and MK2-dependent signaling^23^ and may be involved in the regulation of proteasomal degradation of the ARE-mRNA decay factor AUF1^24^.

## Methods

### Preparation and cultivation of murine bone marrow-derived macrophages

The establishment, genotyping, and colony maintenance of mice deficient for MK2 or for MK3 have been described elsewhere^25,26^. Wild-type bone marrow-derived macrophages (wt BMDM), MK2-deficient BMDM (MK2^−/−^ BMDM) and BMDM deficient in MK2 and 3 (MK2/3^−/−^ BMDM) were prepared and cultivated as recently described^12^. The prepared BMDM were 96.7% (±3.6) F4/80-positive, 99.9% (±1.0) CD11b-positive and 71.3% (±2.5) CD11c-negative as assessed by flow cytometry analysis. The medium was changed to M-CSF-free culture medium 1 h before experiments were performed. Cells were used after a total differentiation period of 9 days. For experiments with immortalized murine bone marrow-derived macrophages (BMDM^*IM*^), cells described previously were used^5,12^.

### Animals

The MK2-, MK3- and MK2/3-deficient animals on a C57BL/6J background have been previously described^9,22,26^. Animals were handled and housed under specific pathogen-free (SPF) conditions. Experiments were carried out in accordance with the German law for animal protection and were approved by the North-Rhine-Westphalia State Agency of Nature, Environment and Consumer Protection (LANUV) under the reference number 84-02.04.2013.A464.

### RNA isolation and real-time PCR

RNA isolation and rtPCR were performed as described previously^5^. The primers used are listed in Supplemental Table 1. The rtPCR specificity was controlled using controls lacking either template or reverse transcriptase. Semi-quantitative PCR results were analyzed using the ΔCT method, and the threshold values were normalized to the expression of hnSDHA.

### Preparation of total cell lysates, cytosolic lysates and nuclear extracts

The preparation of total cell lysates, cytosolic lysates (CL) and nuclear extracts (NE) from murine immortalized or primary BMDM, respectively, was performed as described previously^5^.

### Immunoblotting, immunoprecipitation and immunodetection

A total of 60 µg of protein extracts from cell culture lysates were subjected to SDS gel electrophoresis (8% PAA) and immunoblotted as described previously^5^. For immunodetection, the following primary antibodies were used at a 1:2,000 dilution: rabbit polyclonal anti-IκBβ (Cat. No. DB076, Delta Biolabs, Gilroy, CA), anti-phospho-Ser19/23-IκBβ (Cat. No. 4921), anti-MK2 (Cat. No. 3042), anti-lamin A/C (Cat. No. 2032, Cell Signaling, Danvers, MA), and anti-ubiquitin (Cat. No. ab7780, Abcam, Cambridge, UK); mouse monoclonal anti-MK3 (Cat. No. H00007867-M01, Abnova, Taipei, Taiwan) and anti-p65 (Cat. No. sc-372, Santa Cruz, Dallas, TX); and mouse monoclonal anti-GAPDH (Cat. No. H86504M, Meridian Life Science, Memphis, TN). The immunoprecipitation protocol has been described previously^5^. For the precipitation of polyubiquitinated proteins, a ubiquitinated protein enrichment kit (Cat. No. 662200, Merck, Darmstadt, Germany) was used according to the manufacturer’s instructions.

### Equipment and settings for the detection of immunoblotted proteins

Following an overnight incubation period at 4 °C with the appropriate primary antibody, immunoblots were incubated for 1 h with a 1:6,000 dilution of a horseradish peroxidase-conjugated secondary antibody (Dako/Agilent, Santa Clara, CA) reacting with either rabbit or mouse immunoglobulin. Next, immunoblots were washed 3 times and were then incubated for 1 min with Western Lightning Plus-ECL substrate (PerkinElmer, Waltham, MA). After immunoblots were sealed in a plastic sheet, they were exposed to film (Hyperfilm ECL, Amersham/GE Healthcare, Little Chalfont, UK) in the dark for between 10 sec and 3 min, depending on the signal intensity, for chemiluminescence detection. For this procedure, film sheets were cut into pieces covering the area on the immunoblot that included the size of the protein of interest according to the protein molecular weight marker (Precision Plus Protein Dual Color Standard, Bio-Rad, Munich, Germany). For film processing, a Curix 60 developer (Agfa, Mortsel, Belgium) was used according to the manufacturer’s instructions. Next, developed films were scanned at a pixel intensity of at least 300 dpi using a Perfection 4990 Photo flatbed scanner (Epson, Suwa, Japan) and Epson Scan Software v3.04 G and were saved as JPEG files. The figures were cropped using XnView image viewer and editor software (Kolor, Francin, France) to set the focus on the protein of interest within the respective immunoblot area. There was no further image processing for brightness or contrast. Uncropped images of the scanned original films from respective immunoblots, which were cropped for Fig. 6, are presented in Supplemental Fig. S7.

### Agilent gene chip analysis

Wild-type, MK2^−/−^ or MK2/3^−/−^ BMDM were stimulated with 100 ng/ml LPS for 120 min or 360 min. For both time points, four completely independent experiments were performed. The integrity of the isolated RNA was assessed using an Agilent 2100 Bioanalyzer (Agilent Technologies, Waldbronn, Germany). All samples in this study showed high-quality RNA integrity numbers (RIN 9,6 +/−0,08). RNA was quantified by photometric measurement on a NanoDrop (Thermo Fisher Scientific GmbH, Dreieich, Germany).

Synthesis of cDNA and subsequent fluorescent labeling of cRNA was performed according to the manufacturer’s protocol (One-Color Microarray-Based Gene Expression Analysis/Low Input Quick Amp Labeling; Agilent Technologies). Briefly, 100 ng of total RNA was converted to cDNA, followed by *in vitro* transcription and incorporation of Cy3-CTP into the newly synthesized cRNA. After fragmentation, labeled cRNA was hybridized to Agilent SurePrint G3 Mouse Gene Expression v2 8 × 60 K Microarrays (Agilent Technologies, Boeblingen, Germany) for 17 h at 65 °C and scanned as described in the manufacturer’s protocol.

Signal intensities on 20-bit TIFF images were calculated using Feature Extraction software (Agilent Technologies, Boeblingen, Germany). Data analyses were conducted with GeneSpring GX (Agilent Technologies, Boeblingen, Germany). Probe signal intensities were quantile normalized across all samples to reduce interarray variability^27^. Input data preprocessing was concluded by baseline transformation to the median of all samples.

### Microarray analysis

Data sets with 55,819 probe sets per array were compared using Microsoft Excel (Microsoft, Redmond, WA). To avoid negative ratios, average intensity differences of <5 were first set to 5. Data were normalized by the mean using the untreated wild-type sample as the baseline in each experiment. To identify differentially expressed genes, we excluded all genes from the analysis that were absent in the test sample (for upregulated genes) or absent in the baseline sample (for downregulated genes) in one or both experiments. Fold changes were calculated separately for the experiments as the ratio of the normalized average intensity difference (test sample) to the normalized average intensity difference (baseline sample). Thresholds were set for the fold change (2-fold and greater, unless otherwise indicated) and absolute difference (at least 500) between the normalized average intensity differences. The consistency between experiments varied between samples depending on the treatment and time point. To minimize the number of false positives, only genes that reproducibly met all the thresholds described above in independent experiments were considered differentially expressed.

### Further analysis of microarray data to identify distinct gene expression patterns

From the 55,819 measured probes, only those that corresponded to a gene of known function according to the NCBI database were further analyzed, restricting the dataset to 33,325 probes. If genes were proven by multiple probes, only the probe with the highest induction in wt BMDM after stimulation with 100 ng/ml LPS was selected for further analysis. Therefore, the expression levels of 24,288 genes in wt BMDM and in BMDM deficient in MK2 or both MK2 and MK3 were analyzed. Data were normalized to the mean expression value in untreated wt BMDM. Differential expression was assessed using the two-sample Student’s t-test. All computations were conducted using MATLAB R2014a (The MathWorks, Inc., Natick, MA).

### Statistical evaluation and densitometry

Statistics were calculated using GraphPad Prism 6 software. Significance was calculated using two-way ANOVA with the Bonferroni test. p < 0.05 was considered to be significant. Densitometric analyses of immunoblots were performed using ImageJ software 1.49 from the National Institutes of Health, USA. Immunoblot bands were marked using the selection mode of the software, and the analyze/measure option was selected to determine the mean signal intensity of the protein of interest (IκBβ) as well as that of the loading control (GAPDH) for normalization (Fig. 6e,f).

## Supplementary information

Supplemental figures

Supplemental figure 2

Supplemental figure 3

Supplemental figure 4

Supplemental table 1

## Data Availability

The datasets generated and analyzed during the current study are available in the GEO repository, https://www.ncbi.nlm.nih.gov/geo/, under accession number GSE123043.
