# Supplementary material for: Cooperative and distinct functions of MK2 and MK3 in the regulation of the macrophage transcriptional response to lipopolysaccharide
Source: Sci Rep. 2019 Jul 30;9:11021. doi: 10.1038/s41598-019-46791-8 (PMC6667695; doi:10.1038/s41598-019-46791-8)
Supplement: Supplementary file 1 — Supplemental figures [file 41598_2019_46791_MOESM1_ESM.pdf]

Cooperative and distinct functions of MK2 and MK3 in the regulation of the macrophage transcriptional response to lipopolysaccharide

(Short title: MK2 and MK3 regulate the LPS-induced inflammatory response of macrophages)

Supplemental figures

Christian Ehlting[1], Julia Rex[2], Ute Albrecht[1], René Deenen[3], Christopher Tiedje[4,5], Karl Köhrer[3], Oliver Sawodny[2], Matthias Gaestel[4], Dieter Häussinger[1], Johannes Georg Bode[1*]

[1] Clinic for Gastroenterology, Hepatology and Infectiology, University Hospital, Medical Faculty, Heinrich Heine University of Düsseldorf, Germany

[2] Institute for System Dynamics, University of Stuttgart, Germany

[3] Biological and Medical Research Center (BMFZ), Genomics & Transcriptomics Laboratory, Heinrich Heine University of Düsseldorf, Germany

[4] Institute of Physiological Chemistry, Hannover Medical School, Hannover, Germany

[5] Department of Cellular and Molecular Medicine, University of Copenhagen, Copenhagen, Denmark

[*] Corresponding author

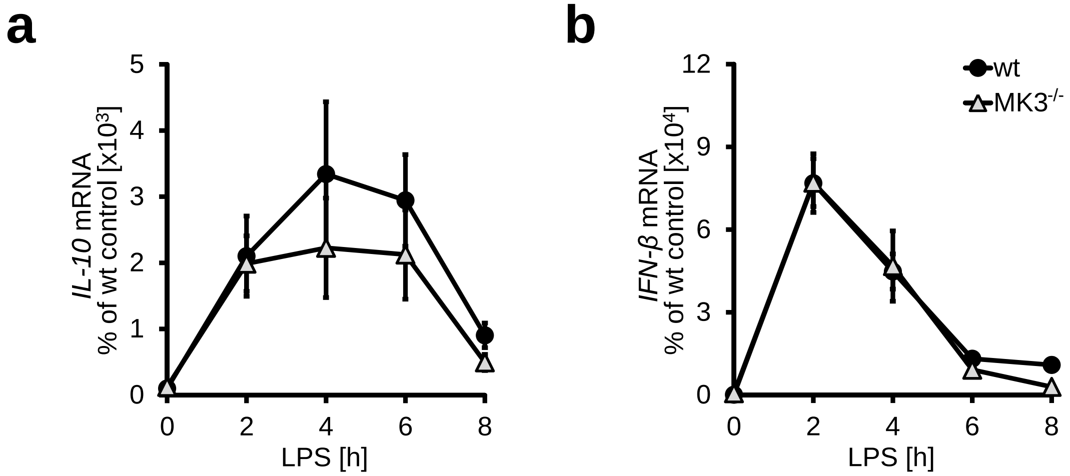

supplemental figure S1

a 2h LPS (mean)

	Gene	wt vs. $MK2^{-/-}$	wt vs. $MK2/3^{-/-}$	$MK2^{-/-}$ vs. $MK2/3^{-/-}$
1	Ifnb1	330,9	226,9	68,57
2	Cxcl1	126,3	347,3	275,1
3	Il1a	125,7	167,6	133,4
4	Cxcl10	97,74	68,44	70,03
5	Cxcl2	146,2	436,9	298,8
6	Cxcl3	79,91	215,5	269,7
7	Il1b	108,5	131,6	121,3
8	Csf2	58,84	218,5	371,4
9	Ccl12	146,2	67,13	45,92
10	Socs1	109,8	40,49	36,87
11	Gem	69,53	46,47	66,84
12	Il23a	95,06	345,1	363
13	Mx2	97,95	58,29	59,51
14	Gm13889	40,6	45,14	111,2
15	Marcksl1	103,2	65,01	63
16	Il2	179	834	465,9
17	Ccrl2	79,57	27,52	34,59
18	Il10	288,6	931	322,6
19	Gm14047	27,21	45,84	168,5
20	Slc7a11	52,54	85,45	162,6
21	Sdc4	58,96	19,53	33,12
22	Mab21l3	49,71	37,17	74,78
23	4933432I03Rik	60,94	78,85	129,4
24	Adora2b	163,2	342,7	210
25	Tcfec	72,31	78,52	108,6
26	Tmem200b	53,89	113,2	210
27	Mmp10	151,1	259,7	171,8
28	Ednrb	75,53	160,7	212,8
29	Il33	63,43	37,96	59,85
30	Nod2	103,5	83,04	80,2
31	Rel	81,94	114	139,1
32	Serpinb2	324,1	501,8	154,9
33	Oaf	118,3	185,3	156,6
34	Flrt3	111,2	296,3	266,4
35	5730559C18Rik	65,8	110,9	168,5
36	Nfkbie	97,72	119,4	122,2
37	Ppp1r15a	90	50,89	56,54
38	Fam55c	62,64	83,69	133,6
39	Htra4	42,38	28,52	67,28
40	Bcl2l15	219,9	446,8	203,2
41	Etnk2	221	229,8	104
42	Plekha4	68,52	36,18	52,81
43	Nipal1	94,26	140,6	149,2
44	Foxp4	58,33	52,85	90,61
45	Tnip3	74,76	50,81	67,97
46	Hamp	81,83	25,15	30,73
47	Cd83	61,66	40,38	65,49
48	Herc6	326	100,8	30,91
49	Klf7	66,41	57,45	86,5
50	4933439G12Rik	83,89	157,4	187,7
51	Shbg	195,8	345,7	176,6
52	Cav1	81,74	212,2	259,6
53	Fabp6	86,2	178,6	207,2
54	Pard6g	79,14	119,6	151,2
55	Pydc3	4802	1283	26,72
56	Plscr1	66,47	71,16	107,1
57	Cdkn1a	88,98	60,41	67,89
58	2500002B13Rik	82,67	222,5	269,1
59	Dusp16	116,4	52,9	45,45
60	Adhfe1	52,31	70,89	135,5
61	Plaur	134,7	96,21	71,41
62	Gpr4	91,4	169,1	185
63	Trim13	114,8	163,4	142,3
64	3930401B19Rik	93,11	143,9	154,6
65	Csf3	441,8	222	50,24
66	F930015N05Rik	47,97	28,6	59,63
67	Cd44	51,07	45,15	88,4
68	5830432E09Rik	111,4	218,1	195,8
69	Phldb1	78,65	76,74	97,57
70	Txk	133,8	187,4	140
71	Gpr132	83,11	62,95	75,74
72	Plagl1	69,22	39,04	56,4
73	E230025N22Rik	105,6	184	174,3
74	Osgin2	121,3	138,2	114
75	Rnd3	93,01	55,58	59,76
76	Lag3	90,69	23,04	25,41
77	Cass4	64,23	62,03	96,58
78	Srgn	81,49	69,42	85,19
79	F830208F22Rik	62,89	54,53	86,71
80	Slc16a10	128,5	153,9	119,8
81	1600029D21Rik	134,5	205,9	153,1
82	Nfil3	94,36	38,94	41,27
83	Arid5a	74,29	24,78	33,36
84	4930469K13Rik	64,21	39,3	61,21
85	Il1r1	155,5	227,3	146,2
86	Ier3	187,3	89,64	47,85
87	Tmem39a	72,8	61,1	83,93
88	Ampd3	55,3	63,46	114,8
89	Pgf	274,9	328	119,3
90	Tagap1	74,54	35,17	47,19
91	Cd59b	581,1	231,8	39,9
92	Zswim4	77,35	48,66	62,9
93	Fndc7	106,5	171	160,5
94	Nod1	564	78,21	13,87
95	Pcdh7	122,8	195,3	159,1
96	Pvrl2	68,02	43,91	64,55
97	Ppap2b	130,3	277,7	213,2
98	Tmem27	30,6	37,91	123,9
99	Slc25a37	82,01	66,09	80,59
100	AW061096	133,2	279,1	209,5
101	Rffl	58,54	53,09	90,69
102	Rcor2	172,3	189,4	109,9
103	Ppm1k	241	98,61	40,92
104	Rcan1	86,6	69,79	80,6
105	Ifna12	274,9	154,2	56,08
106	Rbpj	86,47	55,91	64,66
107	Tagap	78,73	38,25	48,59
108	Unc93a	61,56	80,57	130,9
109	Igsf9	63,29	38,25	60,43
110	Itga5	92,63	83,45	90,09
111	A130078K24Rik	56,4	42,53	75,4
112	Gm505	156,4	138,3	88,41
113	Eif4e	193,8	429,4	221,6
114	Dnajb2	61,61	65,95	107
115	1700109H08Rik	41,39	51,54	124,5
116	Ifrd1	152,9	146,9	96,08
117	Rbm33	221,2	621	280,7
118	Dusp4	101,9	251,8	247,2
119	Pdpn	198,5	284,4	143,3
120	Tnfsf18	53,71	20,25	37,71
121	Phxr4	30,41	17,89	58,83
122	Itpkc	80,16	43,47	54,23
123	Arg2	77,93	64,03	82,17
124	B3gnt2	94,16	51,61	54,81
125	Cdk12	75,25	47,2	62,72
126	Itpka	67,89	40,23	59,25
127	4833422F24Rik	134,4	202,7	150,8
128	Mobkl2c	66,63	38,97	58,49
129	Rasgef1b	69,74	61,47	88,14
130	Zfp296	105	49,79	47,4
131	Baz1a	100,6	79,24	78,75

supplemental figure S2

b

6h LPS (mean)

	Gene	wt vs. $MK2^{-/-}$	wt vs. $MK2/3^{-/-}$	$MK2^{-/-}$ vs. $MK2/3^{-/-}$
1	Il19	155,4	525,4	338
2	Il6	88,85	78,94	88,85
3	Hdc	97,9	137,4	140,3
4	Il12b	57,28	58,13	101,5
5	Ifnb1	727,7	347,3	47,73
6	Cxcl3	36,85	279,2	757,5
7	Shisa3	47,73	156,5	327,9
8	Cxcl1	68,17	201,1	295
9	Cxcl9	93,57	23,18	24,78
10	Cd40	81,66	71,44	87,49
11	Cd69	124,3	52,97	42,63
12	Serpina3f	608,1	71,04	11,68
13	Csf3	141,4	765,6	541,5
14	Saa2	331,1	5276	1594
15	Fpr2	48,52	53,97	111,2
16	Socs3	134,3	147,4	109,7
17	Iigp1	138	66,12	47,93
18	Niacr1	72,68	102,1	140,5
19	Il10	292,3	516,7	176,8
20	Trim30c	119,8	145,7	121,6
21	Lcn2	152,4	419,7	275,3
22	Tnf	119	68,71	57,75
23	Serpina3g	189,4	54,63	28,85
24	Csf2	31,7	150,3	474,2
25	Saa3	98,19	319	324,9
26	Lhx2	145,4	53,95	37,11
27	Mmp13	70,94	62,37	87,92
28	Usp18	149,6	121,7	81,34
29	AW112010	107	76,92	71,89
30	Gbp2	88,25	77,3	87,59
31	Saa1	109,8	332	302,3
32	Slc28a2	185,1	155,4	83,96
33	D730047E02Rik	46,58	59,17	127,1
34	Socs1	119	169,9	142,9
35	Gm14085	164,9	139,4	84,55
36	Ptges	64,85	157,8	243,4
37	Tnfsf10	169,4	95,87	56,61
38	Mmp25	103,9	63,74	61,35
39	Gm9706	148,4	98,94	66,67
40	Ms4a4c	64,27	68,64	106,8
41	Steap4	458,9	1168	254,5
42	Il27	155	58,42	37,68
43	Gm10522	117,4	672,3	572,5
44	Ifit2	105,6	66,25	62,73
45	Oasl1	94,41	85,41	90,48
46	Batf	76,36	115,4	151,1
47	Cd200	34,54	30,42	88,06
48	Isg15	73,62	70,41	95,65
49	LOC100503637	71,35	87,88	123,2
50	Pydc3	2835	794,7	28,03
51	Gm4951	154,7	61,52	39,77
52	Ptx3	138,7	349	251,6
53	LOC100503337	82,87	158	190,7
54	Ccnd2	70,44	63,84	90,64
55	Gbp4	133,2	72,96	54,77
56	Tnfsf15	40,13	39,53	98,5
57	Ms4a4b	71,49	56,03	78,36
58	Ccl17	43,2	168,9	390,9
59	Upp1	83,99	45,96	54,71
60	Plat	37,26	54,38	146
61	U90926	66,92	289,8	433,1
62	Pfkfb3	127,2	980,6	771,1
63	Lipg	278,8	218,4	78,31
64	Rnd1	133	1014	761,8
65	Ifi47	95,92	83,61	87,16
66	Herc6	379	170,8	45,05
67	Apol9b	155,2	342,6	220,7
68	Il1f9	337,6	1725	511,1
69	Adora2b	131,4	208,4	158,5
70	Trim30b	182,6	193,5	106
71	Enpp4	143,7	152,9	106,5
72	Adhfe1	53,74	74,47	138,6
73	Gch1	117,7	116,7	99,18
74	Phf11	116	80,13	69,08
75	Aqp9	163,2	614,7	376,8
76	Atp10a	58	68,51	118,1
77	D14Ertd668e	73,65	57,81	78,49
78	Ccrl2	81,22	37,95	46,73
79	Lta	58,4	40,9	70,03
80	Cish	77,57	179,9	231,9
81	Plekha4	100,9	66,52	65,92
82	Slc7a2	69,63	67,76	97,31
83	Slamf7	110,3	76,67	69,49
84	LOC100417829	146,9	66,88	45,51
85	Slc7a11	51,35	191,3	372,6
86	AA467197	90,18	80,47	89,24
87	Il17rd	87,61	319,8	365
88	Ppef2	118,9	348,7	293,3
89	Serpinb2	176,5	961,1	544,4
90	4933432I03Rik	48,8	45,88	94,02
91	Igtp	143,8	165,1	114,8
92	Sdc4	38,62	13,78	35,67
93	Fcgr2b	78,58	185,6	236,1
94	Nod1	240,6	120,8	50,21
95	Fgl2	120,5	51,89	43,08
96	BC006779	79,13	59,98	75,8
97	1190003J15Rik	189,4	562,3	296,8
98	Btla	28,8	14,73	51,14
99	A430084P05Rik	79,19	34,65	43,76
100	Gm10851	174,7	536,2	306,9
101	1200009I06Rik	73,6	108,6	147,6
102	Gm14047	15,79	24,99	158,3
103	Clec4e	59,48	79,94	134,4
104	Sele	53,48	10,94	20,45
105	Ier3	115,7	212	183,3
106	Gbp9	113,5	71,76	63,25
107	Gda	85,64	166,2	194,1
108	Lag3	83,11	40,11	48,26
109	Tiparp	119,8	183,6	153,2
110	Apol9a	148,1	298,5	201,6
111	Tslp	51,09	166,8	326,5
112	Gnb4	79,29	55,64	70,18
113	Arid5a	152,8	111	72,66
114	Tnfaip6	1034	862,6	83,45
115	Kdm5b	156,4	66,61	42,59
116	Rab11fip1	58,15	101,1	173,8
117	Tmem2	148,6	202,8	136,5
118	Lrrc4	89,72	17,39	19,38
119	Fpr-rs6	190,6	54,76	28,72
120	Ddx60	216	101,6	47,04
121	Il4ra	255,9	784,2	306,4
122	Dusp16	221,9	289,3	130,3
123	Il23r	201,2	184,7	91,83
124	Trp53i11	88,79	145	163,3
125	Hp	313,7	978	311,7
126	Sytl3	44,2	18,83	42,6
127	5730508B09Rik	119,7	85,52	71,43
128	Nt5c3	124,5	92,76	74,54
129	Tgtp1	138,1	64,81	46,92
130	Fcgr4	102,9	57,44	55,83
131	Ehd1	67,72	71,99	106,3
132	Ddx58	119,5	124,2	103,9
133	LOC668917	134,2	114,5	85,32
134	Rhou	211,5	1559	737,3
135	Gm614	90,19	51,17	56,74
136	H2-M2	34,22	52,99	154,9
137	Rapgef2	85,89	50,43	58,72
138	Hivep2	263,6	354,1	134,3
139	Fgl1	53,34	37,71	70,71
140	Gpr31c	60,31	43,25	71,71
141	Dusp1	147,8	154,5	104,6
142	Pdpn	75,93	267	351,6
143	Csprs	189	104,8	55,41
144	Nox1	163,4	183,2	112,1
145	Tnfaip2	73,21	132,5	180,9
146	S100a8	327,8	884,7	269,9
147	Map3k8	124	168	135,5
148	Slc39a14	89,97	290,8	323,2
149	Nudt17	43,91	29,03	66,1
150	Zc3h12a	113,7	133	117
151	I830077J02Rik	253,3	160,3	63,29
152	Rtn1	50,37	72,04	143
153	Ebi3	73,66	75,39	102,4
154	LOC546061	206,1	89,29	43,32
155	Plagl2	99,12	175,6	177,1
156	Zeb1	107,2	205,9	192
157	Gbp11	481,7	83,75	17,39
158	Slc25a22	164,2	99,01	60,28
159	Cd247	58,3	121,8	209
160	Atic	90,64	61,98	68,38
161	Procr	57,57	32,31	56,12
162	Fdxacb1	146	92,28	63,2
163	Flrt3	65,71	271,2	412,8
164	Tap1	104,9	84,89	80,89
165	Irf7	79,2	62,63	79,08
166	Ankrd33b	67,5	49,96	74,01
167	Rcl1	87,32	161,8	185,3
168	Il12a	49,79	11,29	22,68
169	Atp8a1	144,3	95,39	66,09
170	Xaf1	73,95	78,83	106,6
171	Klf7	86,36	155,3	179,8
172	Golga7b	167	114,8	68,75
173	Gm9883	161	182,7	113,5
174	Nfkb2	70,1	81,27	115,9
175	Mab21l3	40,95	14,2	34,68
176	Pla2g4a	134,4	133,4	99,27
177	A130040M12Rik	54,95	60,83	110,7
178	Ext1	97,26	125,9	129,5
179	3930401B19Rik	66,67	61,66	92,49
180	Fam55c	46,91	45,82	97,69
181	1100001G20Rik	195,1	423	216,8
182	Ripk2	154,5	87,55	56,68
183	Gm6907	164,9	94,05	57,03
184	Dgat2	148,5	432,5	291,4
185	Tor1aip2	114,7	94,57	82,44
186	9130017N09Rik	101,8	227,5	223,6
187	Ddx4	223,5	165,5	74,04
188	Bcl2l15	264,1	415,2	157,2
189	Aldh1b1	162,3	76,6	47,2
190	Ttll2	76,5	34,69	45,34
191	Naa25	130,3	140,9	108,2
192	Mtmr7	74,78	54,51	72,89
193	Magohb	98,36	137,3	139,5
194	Spint2	81,68	296,4	362,8
195	Art3	268,8	163,8	60,95
196	Tcstv3	120,5	55,18	45,81
197	4932415M13Rik	154,5	38,17	24,71
198	Shbg	191,7	289,8	151,2
199	Fst	137,4	37,89	27,57
200	Cx3cl1	97,89	423,3	432,5
201	Tmed5	229,4	105,6	46,02
202	Oas1g	182,4	161,5	88,51
203	Samsn1	94,37	147,6	156,4
204	Slc6a4	154,9	336,5	217,2
205	Ms4a6b	94,58	47,48	50,2
206	Rel	236,3	57,47	24,33
207	Gm4759	146,1	68,39	46,81
208	Tmem171	109,5	63,77	58,22
209	Nfil3	107	288,1	269,2
210	LOC100503357	179,4	100,8	56,2
211	Macc1	17,98	31,01	172,5
212	Gm10649	151,9	32,54	21,42
213	Gm8369	100,9	52,5	52,03
214	Sod2	83,03	106	127,7
215	G630030J09Rik	231,2	818,9	354,2
216	9030625A04Rik	52,81	38,76	73,39
217	Ets2	81,89	102,7	125,4
218	Olfr1417	139,7	86,79	62,13
219	Zfp36	158,2	210	132,7
220	Mthfd2	162,4	221,1	136,2
221	Acsl1	55,97	101,7	181,6
222	Gypc	108,5	62,37	57,51
223	AA960436	129,6	129,9	100,2
224	Nlrp3	62,7	53,95	86,06
225	Trim21	102,7	145,4	141,6
226	AI447881	289,7	178,3	61,55
227	Rin2	77,1	48,25	62,58
228	Gm1966	62,53	54,03	86,41
229	Tmem67	125,8	96,68	76,88
230	Tnnt2	148,1	625,3	422,1
231	Unc93a	90,94	46,58	51,22
232	Ikbke	66,65	59,21	88,83
233	Ambp	125,1	337	269,3
234	LOC236220	143,8	65,97	45,87
235	Dusp5	92,89	39,95	43,01
236	Parp14	87,21	80,86	92,71
237	Nmi	147,7	123,4	83,6
238	Gvin1	71,26	68,69	96,41
239	Gm10537	102,4	63,97	62,45
240	Nrg4	162,6	225,9	138,9
241	Etnk2	362,7	391,4	107,9
242	Cd83	37	21,78	58,86
243	Rln3	111,9	66,96	59,85
244	Clec2d	150	94,01	62,67
245	Mfsd7a	122,1	160,7	131,6
246	Trim12c	77,28	84,9	109,9
247	Col27a1	47,78	94,65	198,1
248	N4bp1	115,1	140,7	122,3
249	Txlnb	61,79	61,84	100,1
250	Aida	78,69	67,65	85,97
251	Etv6	96,07	76,9	80,04
252	Hopx	66,65	34,01	51,02
253	Flnb	75,17	67,78	90,16
254	Iigp1b	592,5	99,13	16,73
255	Lass6	65,99	66,84	101,3
256	St3gal1	75,82	92,35	121,8
257	D16Ertd472e	79,19	162,9	205,7
258	Gm9789	163,1	67,06	41,11
259	Tnfrsf1b	97,86	147,5	150,7
260	Papd7	99,36	71,96	72,43
261	Ppfia3	43,59	88,27	202,5
262	B430306N03Rik	223,2	119,3	53,44
263	Clic4	98,56	78,42	79,57
264	Cebpd	185,9	485,3	261
265	Clcf1	64,32	41,21	64,07
266	Lrrc8c	83,03	95,38	114,9
267	Arhgef3	63,01	50,66	80,39
268	Fam49a	95,94	133,6	139,3
269	Stard7	100,5	136,1	135,5
270	Olfr832	230,4	100,2	43,5
271	4732419C18Rik	276,6	1289	466
272	Elavl4	159,4	102,5	64,31
273	Areg	32,03	95,39	297,8
274	Gm5483	382,3	1044	273
275	Ccr9	73,24	79,11	108
276	Tmem39a	70,29	39	55,49
277	Scube3	112,2	392,5	349,8
278	Stk40	103,4	170,7	165,1
279	Epb4.1l4b	147	337,7	229,8
280	Gja1	362,7	448,8	123,7
281	Tank	102,2	85,71	83,83
282	Ncoa7	113,9	69,81	61,32
283	Casp7	85,9	121,6	141,6
284	Tyms	144,9	134,9	93,12
285	Pml	122,8	89,33	72,72
286	Gm5544	121,9	201,6	165,3
287	Ms4a6c	75,42	69,69	92,4
288	Prl6a1	67,25	42,22	62,78
289	Stard5	123,4	150,6	122
290	B3gnt2	124,8	83,61	67,02
291	Prm1	150,1	41,38	27,57
292	Calcrl	108,1	352,1	325,7
293	Rnf213	74,92	60,79	81,14
294	Adora3	560,5	1111	198,2
295	Ednrb	85,34	217,9	255,3
296	Pard6g	211,5	108,6	51,34
297	Alpk2	61,66	140,8	228,4
298	Pax3	107,6	78,5	72,92
299	Fam82a2	103,3	69,28	67,04
300	Dtx2	112,4	163	145,1
301	Cd86	78,49	50,4	64,21
302	Parp10	210,2	175,1	83,3
303	9930111J21Rik1	117	79,59	68
304	4930420K17Rik	82,7	68,87	83,29
305	Rbpms	129,3	175,2	135,5
306	Srgn	66,52	61,85	92,98
307	D2Bwg0886e	148,3	86,19	58,11
308	Tha1	178,1	400,9	225
309	Kcnn3	133,9	61,24	45,74
310	Tbrg4	137,3	83,61	60,88
311	Lphn2	105	286,5	272,8
312	Osgin2	83,14	61,64	74,15
313	Ccdc25	100,3	67,3	67,1
314	Zfp599	168,6	995,2	590,3
315	Gcnt2	284,7	570,2	200,3
316	BC147527	83,6	49,83	59,6
317	Krtap16-10	123,3	46,59	37,8
318	Rab20	54,08	47,63	88,08
319	Ppa1	137	90,66	66,19
320	Tpst1	260,5	160,2	61,49
321	Camta1	78,21	306,3	391,6
322	Bcl3	121,9	265,1	217,6
323	Ms4a6d	64,5	65,8	102
324	Gstt1	113,4	174,9	154,3
325	Pnp	90,65	91,81	101,3
326	Stat2	73,44	73,6	100,2
327	Tlr1	62,99	65,21	103,5
328	Kdm4a	60,83	98,6	162,1
329	Lck	77,43	17,55	22,67
330	Mcoln2	88,38	221,5	250,6
331	Snx10	117,2	125,2	106,9
332	Mtdh	65,9	75,69	114,9
333	Ggct	537,3	93,81	17,46
334	Prokr1	400,9	2288	570,9
335	Gfpt2	226,3	254,7	112,6
336	Rnf19b	103,6	64,92	62,7
337	Gas7	67,43	157,5	233,6
338	Maff	59,65	73,29	122,9
339	Fscn1	44,06	69,23	157,1
340	Gna13	87,4	64,34	73,61
341	Glipr2	142,8	98,7	69,13
342	Wars	134,1	120,6	89,95
343	Flt1	162,3	325,1	200,4
344	Ppm1k	442,2	108,7	24,58
345	Spink2	245,9	185,8	75,58
346	Osm	59,82	54,75	91,53
347	Tagap1	93,45	66,76	71,44
348	Pou3f1	101,3	41,21	40,7
349	Slfn9	44,69	42,23	94,48
350	Phactr1	170,9	303,4	177,6
351	Pdss1	118,2	74,93	63,41
352	Gm10791	149,7	63,92	42,7
353	Sco1	105,5	57,99	54,95
354	Slamf8	54,87	42,13	76,78
355	Bco2	49,69	49,72	100,1
356	Trem3	120,6	370,2	306,9
357	1700054O13Rik	30,43	30,44	100
358	Carhsp1	125,4	76,89	61,33
359	Lsm14a	135,1	94,18	69,69
360	Tcfec	74,65	163	218,3
361	Aff1	186,3	286,5	153,8
362	Slc7a5	207,7	432,2	208,1
363	Zfp459	537,8	1141	212,1
364	Ddit4	114,1	606,1	531
365	Insl6	132,9	148,5	111,7
366	St7	70,45	118,2	167,8
367	Itln1	226,5	378,4	167,1
368	Htra4	35,06	14,02	39,98
369	Pnpt1	141,2	146,1	103,5
370	Slc7a7	131,9	195,7	148,4
371	C1ql2	116,6	51,01	43,76
372	4921513D23Rik	84,47	84,84	100,4
373	Chrna5	239,8	69,71	29,08
374	Arg2	168,5	869,1	515,7
375	Vasp	75,16	100,5	133,7
376	Pttg1	101,5	83,42	82,17
377	Hoxb5	230,8	147	63,68
378	Sgk3	107	138,8	129,7
379	Olfr187	301,4	114,6	38,03
380	Rin3	59,27	88,35	149,1
381	Prkx	79,01	62,79	79,47
382	Hoxd13	601,8	85,99	14,29
383	Fhod3	219	583,5	266,5
384	Pfkp	101,2	71,35	70,48
385	1190002H23Rik	122,5	47,06	38,42
386	Gm3367	76,8	35,82	46,64
387	Osta	150,6	412,1	273,7
388	Olfr560	84,84	46,88	55,26
389	Tor1aip1	92,53	67,54	72,99
390	Tagap	95,86	58,63	61,16
391	Src	122,8	190,2	154,9
392	Pik3r5	97,56	211,5	216,8
393	Lnpep	82,75	51,49	62,23
394	Paqr5	153	60,81	39,75
395	Lrrc25	133,1	188,5	141,7
396	Sbno2	128,3	289,3	225,5
397	Fam160a1	388,1	224,6	57,86
398	Pilra	67,83	51,32	75,66
399	Gm10404	150,3	135,9	90,42
400	Ido1	560,2	274,1	48,93
401	Tmem161a	133	70,87	53,29
402	BC013712	121,6	63,15	51,93
403	Piwil4	175,2	550,3	314,1
404	Cebpb	103,7	216,4	208,8
405	Dbnl	82,09	76,46	93,14
406	Oas1f	127,4	93,51	73,39
407	Dgkh	98,14	63,63	64,84
408	1700047I17Rik2	72,2	118,2	163,8
409	Fosl2	108,7	252,1	232
410	1600014C10Rik	107,5	65,87	61,28
411	2400003C14Rik	109,4	137,2	125,4
412	Mmp28	221	126,2	57,12
413	Gm2701	110,3	59,5	53,94
414	5730528L13Rik	130,6	208,6	159,7
415	Col11a2	86,51	123,1	142,3
416	Fcrlb	92,39	209,3	226,6
417	Mcmbp	91,75	56,43	61,5
418	Itgb6	210,9	327,5	155,2
419	4933404M02Rik	228,6	311	136,1
420	Irak3	72,16	165,5	229,3
421	Tmem170b	125,9	170,4	135,4
422	A630012P03Rik	340,2	112,6	33,1
423	Tlr6	103,1	63,91	61,97
424	Prkag3	155,5	47,74	30,71
425	Wdr43	140,6	141,8	100,8
426	Slc2a1	71,24	120,6	169,3
427	Ifrd1	72,64	62,08	85,45
428	Bcl2l1	104,4	86,92	83,23
429	C1ra	184	76,3	41,47
430	A630001G21Rik	72,23	57,03	78,96
431	A530064D06Rik	306,9	202,1	65,84
432	3110003A17Rik	70,58	85,53	121,2
433	Psme2	80,28	86,76	108,1
434	Gm10414	147,4	268,3	182
435	D17Wsu92e	77,68	90,76	116,8
436	Fam133b	65,36	42,43	64,92
437	Sell	85,52	32,88	38,44
438	Ptprj	88,03	108,3	123
439	Sept11	69,87	104,8	150,1
440	Wdr16	58,64	48,96	83,49
441	Gm5712	393,7	147,4	37,43
442	Golga3	132,1	99,88	75,62
443	Lnx1	243,9	108	44,27
444	Irak2	76,84	63,05	82,07
445	2810408A11Rik	125	75,14	60,1
446	Znfx1	100,9	77,16	76,51
447	Trim36	41,4	33,43	80,74
448	Ranbp2	92,67	65,1	70,25
449	Sbds	60,1	40,9	68,05
450	Pvr	58,77	78,06	132,8
451	2210403K04Rik	50,49	57,66	114,2
452	Snx2	93,87	73,54	78,35
453	2200002D01Rik	82,89	170,9	206,1
454	Casz1	68,75	14,74	21,44
455	Odz4	424	143,9	33,93
456	Gm12592	227,4	143,1	62,93
457	Ccdc50	54,45	48,35	88,8
458	Hck	102,5	128,8	125,7
459	Sypl2	276,1	101	36,58
460	Etv2	128,1	409,5	319,7
461	Yipf2	154,8	109,9	70,99
462	Wdr37	105,8	77,99	73,69
463	Gm5039	482,4	161,2	33,42
464	Ms4a8a	290,1	511,9	176,5
465	Rilpl1	183,9	283	153,9
466	Sprr2e	61,29	46,43	75,75
467	Arl5c	40,48	45,59	112,6
468	Ccl9	76,4	102,8	134,6
469	Rnf135	102,6	83,19	81,05
470	Trim12a	128,1	125,8	98,26
471	Dcun1d3	102,6	190,5	185,7
472	Zfp11	138	71,01	51,46
473	BC100530	349	482,4	138,2
474	Trim26	125,7	124,3	98,89
475	LOC100046147	198,8	103,6	52,12
476	Ccdc86	94,72	114,4	120,8
477	Tmtc2	39,05	36,52	93,52
478	Dck	154,2	96,39	62,49

supplemental figure S2

c 2h LPS (p-value)

	Gene	wt vs. $MK2^{-/-}$	wt vs. $MK2/3^{-/-}$	$MK2^{-/-}$ vs. $MK2/3^{-/-}$
1	Ifnb1	0,038	0,052	0,446
2	Cxcl1	0,264	0,000	0,005
3	Il1a	0,282	0,043	0,179
4	Cxcl10	0,859	0,038	0,021
5	Cxcl2	0,009	0,000	0,002
6	Cxcl3	0,544	0,027	0,017
7	Il1b	0,406	0,019	0,066
8	Csf2	0,339	0,055	0,046
9	Ccl12	0,298	0,265	0,019
10	Socs1	0,797	0,098	0,024
11	Gem	0,232	0,025	0,104
12	Il23a	0,906	0,008	0,006
13	Mx2	0,947	0,140	0,032
14	Gm13889	0,028	0,034	0,740
15	Marcksl1	0,864	0,056	0,011
16	Il2	0,257	0,014	0,046
17	Ccrl2	0,340	0,002	0,001
18	Il10	0,045	0,000	0,059
19	Gm14047	0,004	0,022	0,187
20	Slc7a11	0,023	0,522	0,013
21	Sdc4	0,354	0,001	0,082
22	Mab21l3	0,113	0,041	0,514
23	4933432I03Rik	0,035	0,144	0,228
24	Adora2b	0,002	0,000	0,003
25	Tcfec	0,037	0,141	0,632
26	Tmem200b	0,071	0,743	0,036
27	Mmp10	0,387	0,024	0,244
28	Ednrb	0,155	0,164	0,029
29	Il33	0,173	0,042	0,268
30	Nod2	0,799	0,199	0,022
31	Rel	0,191	0,370	0,013
32	Serpinb2	0,015	0,003	0,315
33	Oaf	0,625	0,030	0,146
34	Flrt3	0,777	0,036	0,012
35	5730559C18Rik	0,175	0,649	0,025
36	Nfkbie	0,819	0,016	0,074
37	Ppp1r15a	0,669	0,009	0,032
38	Fam55c	0,030	0,386	0,065
39	Htra4	0,009	0,005	0,112
40	Bcl2l15	0,018	0,001	0,036
41	Etnk2	0,013	0,007	0,877
42	Plekha4	0,278	0,035	0,100
43	Nipal1	0,828	0,220	0,035
44	Foxp4	0,025	0,038	0,642
45	Tnip3	0,228	0,006	0,077
46	Hamp	0,596	0,010	0,018
47	Cd83	0,075	0,006	0,009
48	Herc6	0,033	0,988	0,024
49	Klf7	0,029	0,015	0,185
50	4933439G12Rik	0,450	0,065	0,020
51	Shbg	0,080	0,001	0,105
52	Cav1	0,651	0,120	0,019
53	Fabp6	0,758	0,018	0,173
54	Pard6g	0,358	0,501	0,009
55	Pydc3	0,001	0,143	0,368
56	Plscr1	0,023	0,095	0,726
57	Cdkn1a	0,546	0,014	0,014
58	2500002B13Rik	0,618	0,016	0,036
59	Dusp16	0,515	0,063	0,009
60	Adhfe1	0,015	0,053	0,154
61	Plaur	0,032	0,746	0,041
62	Gpr4	0,684	0,075	0,025
63	Trim13	0,380	0,024	0,025
64	3930401B19Rik	0,700	0,115	0,049
65	Csf3	0,001	0,496	0,560
66	F930015N05Rik	0,124	0,025	0,289
67	Cd44	0,012	0,001	0,500
68	5830432E09Rik	0,654	0,006	0,014
69	Phldb1	0,078	0,048	0,700
70	Txk	0,253	0,032	0,055
71	Gpr132	0,195	0,007	0,059
72	Plagl1	0,309	0,034	0,087
73	E230025N22Rik	0,768	0,007	0,030
74	Osgin2	0,205	0,048	0,326
75	Rnd3	0,800	0,075	0,031
76	Lag3	0,835	0,027	0,033
77	Cass4	0,040	0,096	0,858
78	Srgn	0,168	0,029	0,138
79	F830208F22Rik	0,044	0,027	0,311
80	Slc16a10	0,149	0,014	0,118
81	1600029D21Rik	0,323	0,045	0,287
82	Nfil3	0,806	0,021	0,017
83	Arid5a	0,522	0,041	0,048
84	4930469K13Rik	0,154	0,025	0,135
85	Il1r1	0,213	0,027	0,176
86	Ier3	0,138	0,730	0,025
87	Tmem39a	0,143	0,031	0,144
88	Ampd3	0,033	0,127	0,504
89	Pgf	0,014	0,010	0,444
90	Tagap1	0,276	0,014	0,045
91	Cd59b	0,038	0,397	0,335
92	Zswim4	0,337	0,020	0,063
93	Fndc7	0,788	0,009	0,107
94	Nod1	0,015	0,652	0,014
95	Pcdh7	0,126	0,002	0,023
96	Pvrl2	0,243	0,028	0,190
97	Ppap2b	0,288	0,003	0,001
98	Tmem27	0,099	0,009	0,739
99	Slc25a37	0,387	0,099	0,042
100	AW061096	0,368	0,011	0,044
101	Rffl	0,029	0,023	0,374
102	Rcor2	0,045	0,015	0,709
103	Ppm1k	0,078	0,969	0,043
104	Rcan1	0,410	0,032	0,302
105	Ifna12	0,036	0,387	0,258
106	Rbpj	0,489	0,053	0,024
107	Tagap	0,347	0,023	0,033
108	Unc93a	0,045	0,308	0,025
109	Igsf9	0,065	0,002	0,025
110	Itga5	0,549	0,047	0,433

	Gene	wt vs. $MK2^{-/-}$	wt vs. $MK2/3^{-/-}$	$MK2^{-/-}$ vs. $MK2/3^{-/-}$
111	A130078K24Rik	0,084	0,035	0,308
112	Gm505	0,077	0,042	0,573
113	Eif4e	0,009	0,000	0,002
114	Dnajb2	0,024	0,109	0,787
115	1700109H08Rik	0,011	0,036	0,381
116	Ifrd1	0,099	0,023	0,850
117	Rbm33	0,030	0,006	0,036
118	Dusp4	0,950	0,054	0,034
119	Pdpn	0,172	0,037	0,475
120	Tnfsf18	0,050	0,009	0,074
121	Phxr4	0,050	0,009	0,349
122	Itpkc	0,445	0,002	0,066
123	Arg2	0,103	0,012	0,100
124	B3gnt2	0,814	0,055	0,024
125	Cdk12	0,100	0,004	0,009
126	Itpka	0,189	0,023	0,052
127	4833422F24Rik	0,273	0,011	0,157
128	Mobkl2c	0,266	0,037	0,075
129	Rasgef1b	0,136	0,041	0,365
130	Zfp296	0,801	0,008	0,001
131	Baz1a	0,952	0,058	0,023

supplemental figure S2

d

6h LPS (p-value)

	Gene	wt vs. $MK2^{-/-}$	wt vs. $MK2/3^{-/-}$	$MK2^{-/-}$ vs. $MK2/3^{-/-}$
1	Il19	0,364	0,043	0,046
2	Il6	0,180	0,007	0,080
3	Hdc	0,906	0,060	0,014
4	Il12b	0,055	0,049	0,902
5	Ifnb1	0,016	0,077	0,160
6	Cxcl3	0,095	0,039	0,002
7	Shisa3	0,134	0,289	0,042
8	Cxcl1	0,285	0,076	0,025
9	Cxcl9	0,831	0,000	0,001
10	Cd40	0,214	0,056	0,044
11	Cd69	0,311	0,003	0,001
12	Serpina3f	0,001	0,129	0,000
13	Csf3	0,563	0,015	0,032
14	Saa2	0,015	0,002	0,002
15	Fpr2	0,034	0,043	0,469
16	Socs3	0,011	0,000	0,191
17	Iigp1	0,190	0,062	0,008
18	Niacr1	0,066	0,918	0,041
19	Il10	0,054	0,007	0,238
20	Trim30c	0,215	0,021	0,184
21	Lcn2	0,133	0,021	0,041
22	Tnf	0,688	0,357	0,026
23	Serpina3g	0,034	0,002	0,000
24	Csf2	0,211	0,609	0,008
25	Saa3	0,951	0,037	0,017
26	Lhx2	0,319	0,053	0,011
27	Mmp13	0,023	0,017	0,329
28	Usp18	0,013	0,000	0,063
29	AW112010	0,629	0,062	0,008
30	Gbp2	0,104	0,038	0,175
31	Saa1	0,775	0,052	0,040
32	Slc28a2	0,008	0,047	0,179
33	D730047E02Rik	0,008	0,053	0,300
34	Socs1	0,236	0,026	0,067
35	Gm14085	0,003	0,036	0,141
36	Ptges	0,137	0,125	0,003
37	Tnfsf10	0,077	0,862	0,040
38	Mmp25	0,880	0,018	0,034
39	Gm9706	0,001	0,931	0,009
40	Ms4a4c	0,044	0,136	0,739
41	Steap4	0,007	0,015	0,156
42	Il27	0,253	0,031	0,009
43	Gm10522	0,562	0,002	0,001
44	Ifit2	0,757	0,042	0,028
45	Oasl1	0,489	0,025	0,142
46	Batf	0,032	0,447	0,042
47	Cd200	0,006	0,019	0,725
48	Isg15	0,014	0,004	0,594
49	LOC100503637	0,043	0,357	0,060
50	Pydc3	0,000	0,038	0,098
51	Gm4951	0,219	0,147	0,018
52	Ptx3	0,456	0,055	0,037
53	LOC100503337	0,474	0,085	0,012
54	Ccnd2	0,020	0,004	0,174
55	Gbp4	0,186	0,221	0,049
56	Tnfsf15	0,023	0,019	0,963
57	Ms4a4b	0,009	0,043	0,216
58	Ccl17	0,300	0,372	0,025
59	Upp1	0,293	0,001	0,001
60	Plat	0,003	0,038	0,109
61	U90926	0,446	0,049	0,013
62	Pfkfb3	0,430	0,001	0,001
63	Lipg	0,011	0,013	0,278
64	Rnd1	0,389	0,000	0,001
65	Ifi47	0,656	0,044	0,128
66	Herc6	0,003	0,042	0,007
67	Apol9b	0,093	0,001	0,006
68	Il1f9	0,093	0,012	0,024
69	Adora2b	0,295	0,013	0,012
70	Trim30b	0,055	0,010	0,799
71	Enpp4	0,131	0,022	0,765
72	Adhfe1	0,023	0,155	0,124
73	Gch1	0,039	0,055	0,891
74	Phf11	0,318	0,180	0,021
75	Aqp9	0,300	0,015	0,018
76	Atp10a	0,004	0,078	0,329
77	D14Ertd668e	0,033	0,004	0,097
78	Ccrl2	0,055	0,000	0,001
79	Lta	0,302	0,004	0,383
80	Cish	0,402	0,057	0,012
81	Plekha4	0,963	0,049	0,066
82	Slc7a2	0,171	0,002	0,892
83	Slamf7	0,603	0,106	0,019
84	LOC100417829	0,047	0,333	0,051
85	Slc7a11	0,027	0,003	0,000
86	AA467197	0,205	0,043	0,181
87	Il17rd	0,592	0,016	0,005
88	Ppef2	0,507	0,003	0,004
89	Serpinb2	0,403	0,026	0,043
90	4933432I03Rik	0,051	0,025	0,788
91	Igtp	0,029	0,006	0,225
92	Sdc4	0,122	0,001	0,073
93	Fcgr2b	0,469	0,075	0,006
94	Nod1	0,004	0,084	0,003
95	Fgl2	0,600	0,079	0,006
96	BC006779	0,134	0,018	0,009
97	1190003J15Rik	0,052	0,005	0,008
98	Btla	0,118	0,031	0,400
99	A430084P05Rik	0,496	0,026	0,001
100	Gm10851	0,020	0,000	0,000
101	1200009I06Rik	0,162	0,690	0,033
102	Gm14047	0,020	0,018	0,452
103	Clec4e	0,014	0,191	0,109
104	Sele	0,366	0,019	0,003
105	Ier3	0,428	0,013	0,004
106	Gbp9	0,298	0,016	0,009
107	Gda	0,411	0,039	0,010
108	Lag3	0,727	0,040	0,198
109	Tiparp	0,409	0,033	0,107
110	Apol9a	0,114	0,006	0,035
111	Tslp	0,143	0,245	0,019
112	Gnb4	0,133	0,004	0,029
113	Arid5a	0,039	0,449	0,099
114	Tnfaip6	0,014	0,011	0,805
115	Kdm5b	0,177	0,189	0,004
116	Rab11fip1	0,038	0,969	0,049
117	Tmem2	0,009	0,038	0,199
118	Lrrc4	0,869	0,010	0,013
119	Fpr-rs6	0,089	0,079	0,001
120	Ddx60	0,012	0,965	0,035
121	Il4ra	0,043	0,001	0,002
122	Dusp16	0,019	0,004	0,352
123	Il23r	0,162	0,014	0,816
124	Trp53i11	0,466	0,188	0,033
125	Hp	0,054	0,006	0,018
126	Sytl3	0,139	0,042	0,065
127	5730508B09Rik	0,094	0,046	0,008
128	Nt5c3	0,191	0,463	0,024
129	Tgtp1	0,280	0,051	0,023
130	Fcgr4	0,813	0,032	0,016
131	Ehd1	0,001	0,013	0,416
132	Ddx58	0,003	0,005	0,434
133	LOC668917	0,023	0,783	0,706
134	Rhou	0,169	0,003	0,007
135	Gm614	0,796	0,106	0,011
136	H2-M2	0,014	0,367	0,453
137	Rapgef2	0,558	0,031	0,017
138	Hivep2	0,060	0,015	0,520
139	Fgl1	0,285	0,048	0,537
140	Gpr31c	0,016	0,014	0,179
141	Dusp1	0,202	0,041	0,847
142	Pdpn	0,525	0,064	0,016
143	Csprs	0,017	0,875	0,040
144	Nox1	0,148	0,035	0,629
145	Tnfaip2	0,104	0,245	0,004
146	S100a8	0,095	0,023	0,158
147	Map3k8	0,258	0,006	0,110
148	Slc39a14	0,432	0,000	0,000
149	Nudt17	0,034	0,005	0,182
150	Zc3h12a	0,539	0,218	0,033
151	I830077J02Rik	0,002	0,150	0,110
152	Rtn1	0,035	0,072	0,113
153	Ebi3	0,004	0,034	0,797
154	LOC546061	0,006	0,402	0,001
155	Plagl2	0,944	0,013	0,005
156	Zeb1	0,597	0,063	0,037
157	Gbp11	0,003	0,625	0,003
158	Slc25a22	0,001	0,968	0,041
159	Cd247	0,100	0,264	0,022
160	Atic	0,721	0,015	0,108
161	Procr	0,156	0,017	0,061
162	Fdxacb1	0,012	0,864	0,276
163	Flrt3	0,332	0,126	0,021
164	Tap1	0,655	0,066	0,047
165	Irf7	0,077	0,012	0,092
166	Ankrd33b	0,147	0,019	0,019
167	Rcl1	0,404	0,052	0,010
168	Il12a	0,222	0,024	0,017
169	Atp8a1	0,005	0,919	0,317
170	Xaf1	0,033	0,082	0,628
171	Klf7	0,425	0,133	0,034
172	Golga7b	0,007	0,810	0,457
173	Gm9883	0,215	0,002	0,663
174	Nfkb2	0,003	0,116	0,197
175	Mab21l3	0,026	0,000	0,002
176	Pla2g4a	0,075	0,026	0,949
177	A130040M12Rik	0,002	0,012	0,322
178	Ext1	0,765	0,107	0,020
179	3930401B19Rik	0,006	0,009	0,418
180	Fam55c	0,069	0,047	0,884
181	1100001G20Rik	0,310	0,027	0,139
182	Ripk2	0,192	0,595	0,031
183	Gm6907	0,044	0,466	0,009
184	Dgat2	0,306	0,003	0,004
185	Tor1aip2	0,214	0,313	0,045
186	9130017N09Rik	0,985	0,046	0,353
187	Ddx4	0,022	0,141	0,301
188	Bcl2l15	0,159	0,037	0,420
189	Aldh1b1	0,106	0,198	0,020
190	Ttll2	0,481	0,024	0,088
191	Naa25	0,055	0,040	0,384
192	Mtmr7	0,115	0,028	0,050
193	Magohb	0,903	0,083	0,043
194	Spint2	0,606	0,005	0,010
195	Art3	0,018	0,097	0,166
196	Tcstv3	0,456	0,047	0,025
197	4932415M13Rik	0,080	0,018	0,000
198	Shbg	0,067	0,006	0,039
199	Fst	0,635	0,069	0,030
200	Cx3cl1	0,970	0,044	0,029
201	Tmed5	0,021	0,814	0,011
202	Oas1g	0,012	0,076	0,571
203	Samsn1	0,803	0,187	0,015
204	Slc6a4	0,191	0,001	0,033
205	Ms4a6b	0,873	0,043	0,019
206	Rel	0,026	0,241	0,009
207	Gm4759	0,147	0,206	0,024
208	Tmem171	0,775	0,172	0,018
209	Nfil3	0,728	0,013	0,009
210	LOC100503357	0,010	0,990	0,331
211	Macc1	0,044	0,380	0,580
212	Gm10649	0,379	0,019	0,001
213	Gm8369	0,956	0,036	0,014
214	Sod2	0,038	0,354	0,006
215	G630030J09Rik	0,157	0,001	0,034
216	9030625A04Rik	0,030	0,011	0,034
217	Ets2	0,158	0,814	0,005
218	Olfr1417	0,128	0,467	0,014
219	Zfp36	0,042	0,039	0,236
220	Mthfd2	0,117	0,037	0,302
221	Acsl1	0,044	0,958	0,088
222	Gypc	0,669	0,019	0,015
223	AA960436	0,060	0,033	0,983
224	Nlrp3	0,061	0,029	0,171
225	Trim21	0,861	0,038	0,076
226	AI447881	0,002	0,014	0,048
227	Rin2	0,361	0,010	0,040
228	Gm1966	0,016	0,004	0,281
229	Tmem67	0,048	0,692	0,017
230	Tnnt2	0,422	0,048	0,068
231	Unc93a	0,700	0,000	0,012
232	Ikbke	0,043	0,022	0,211
233	Ambp	0,436	0,025	0,017
234	LOC236220	0,119	0,138	0,023
235	Dusp5	0,839	0,031	0,004
236	Parp14	0,167	0,040	0,279
237	Nmi	0,026	0,113	0,229
238	Gvin1	0,024	0,071	0,811
239	Gm10537	0,916	0,060	0,018
240	Nrg4	0,104	0,045	0,212
241	Etnk2	0,002	0,002	0,646
242	Cd83	0,049	0,010	0,001
243	Rln3	0,650	0,067	0,026
244	Clec2d	0,153	0,786	0,026
245	Mfsd7a	0,416	0,023	0,202
246	Trim12c	0,004	0,051	0,195
247	Col27a1	0,025	0,813	0,023
248	N4bp1	0,326	0,028	0,109
249	Txlnb	0,149	0,026	0,998
250	Aida	0,070	0,004	0,170
251	Etv6	0,785	0,041	0,152
252	Hopx	0,326	0,023	0,029
253	Flnb	0,015	0,061	0,513
254	Iigp1b	0,003	0,986	0,008
255	Lass6	0,007	0,046	0,934
256	St3gal1	0,040	0,734	0,310
257	D16Ertd472e	0,419	0,111	0,014
258	Gm9789	0,126	0,097	0,006
259	Tnfrsf1b	0,789	0,002	0,001
260	Papd7	0,973	0,113	0,037
261	Ppfia3	0,004	0,629	0,023
262	B430306N03Rik	0,038	0,626	0,105
263	Clic4	0,871	0,001	0,018
264	Cebpd	0,020	0,001	0,007
265	Clcf1	0,105	0,025	0,071
266	Lrrc8c	0,032	0,653	0,098
267	Arhgef3	0,056	0,006	0,069
268	Fam49a	0,707	0,058	0,036
269	Stard7	0,983	0,181	0,031
270	Olfr832	0,073	0,995	0,034
271	4732419C18Rik	0,055	0,002	0,001
272	Elavl4	0,028	0,962	0,358
273	Areg	0,083	0,858	0,036
274	Gm5483	0,162	0,008	0,177
275	Ccr9	0,045	0,146	0,512
276	Tmem39a	0,211	0,007	0,018
277	Scube3	0,772	0,027	0,027
278	Stk40	0,825	0,028	0,001
279	Epb4.1l4b	0,455	0,035	0,053
280	Gja1	0,024	0,005	0,559
281	Tank	0,807	0,055	0,045
282	Ncoa7	0,248	0,027	0,001
283	Casp7	0,233	0,214	0,036
284	Tyms	0,037	0,311	0,769
285	Pml	0,191	0,159	0,038
286	Gm5544	0,372	0,002	0,032
287	Ms4a6c	0,056	0,031	0,430
288	Prl6a1	0,105	0,000	0,034
289	Stard5	0,079	0,024	0,126
290	B3gnt2	0,125	0,061	0,008
291	Prm1	0,109	0,005	0,001
292	Calcrl	0,374	0,000	0,000
293	Rnf213	0,077	0,028	0,238
294	Adora3	0,031	0,012	0,259
295	Ednrb	0,671	0,063	0,001
296	Pard6g	0,007	0,736	0,028
297	Alpk2	0,067	0,098	0,003
298	Pax3	0,580	0,081	0,040
299	Fam82a2	0,853	0,016	0,028
300	Dtx2	0,423	0,040	0,087
301	Cd86	0,476	0,048	0,138
302	Parp10	0,003	0,003	0,240
303	9930111J21Rik1	0,369	0,063	0,022
304	4930420K17Rik	0,387	0,034	0,252
305	Rbpms	0,318	0,012	0,162
306	Srgn	0,000	0,001	0,262
307	D2Bwg0886e	0,020	0,687	0,129
308	Tha1	0,027	0,000	0,006
309	Kcnn3	0,260	0,053	0,000
310	Tbrg4	0,008	0,394	0,020
311	Lphn2	0,818	0,069	0,038
312	Osgin2	0,593	0,189	0,022
313	Ccdc25	0,989	0,048	0,039
314	Zfp599	0,553	0,000	0,041
315	Gcnt2	0,018	0,017	0,206
316	BC147527	0,404	0,009	0,022
317	Krtap16-10	0,221	0,068	0,008
318	Rab20	0,008	0,007	0,290
319	Ppa1	0,091	0,538	0,002
320	Tpst1	0,001	0,232	0,145
321	Camta1	0,545	0,078	0,009
322	Bcl3	0,260	0,009	0,013
323	Ms4a6d	0,010	0,057	0,903
324	Gstt1	0,611	0,011	0,077
325	Pnp	0,040	0,029	0,648
326	Stat2	0,101	0,038	0,982
327	Tlr1	0,014	0,082	0,827
328	Kdm4a	0,024	0,951	0,070
329	Lck	0,220	0,000	0,001
330	Mcoln2	0,733	0,058	0,010
331	Snx10	0,178	0,006	0,442
332	Mtdh	0,003	0,095	0,312
333	Ggct	0,000	0,566	0,000
334	Prokr1	0,031	0,000	0,002
335	Gfpt2	0,011	0,044	0,736
336	Rnf19b	0,914	0,046	0,107
337	Gas7	0,292	0,201	0,021
338	Maff	0,016	0,007	0,155
339	Fscn1	0,034	0,390	0,283
340	Gna13	0,590	0,042	0,155
341	Glipr2	0,004	0,949	0,062
342	Wars	0,024	0,278	0,461
343	Flt1	0,198	0,015	0,043
344	Ppm1k	0,005	0,729	0,001
345	Spink2	0,027	0,163	0,476
346	Osm	0,004	0,032	0,611
347	Tagap1	0,693	0,032	0,019
348	Pou3f1	0,979	0,042	0,038
349	Slfn9	0,002	0,019	0,818
350	Phactr1	0,058	0,009	0,074
351	Pdss1	0,360	0,048	0,010
352	Gm10791	0,204	0,203	0,001
353	Sco1	0,850	0,017	0,023
354	Slamf8	0,029	0,041	0,426
355	Bco2	0,004	0,020	0,997
356	Trem3	0,738	0,026	0,081
357	1700054O13Rik	0,012	0,017	0,999
358	Carhsp1	0,197	0,005	0,010
359	Lsm14a	0,042	0,871	0,288
360	Tcfec	0,397	0,215	0,032
361	Aff1	0,142	0,011	0,178
362	Slc7a5	0,169	0,042	0,208
363	Zfp459	0,046	0,002	0,122
364	Ddit4	0,644	0,003	0,000
365	Insl6	0,058	0,034	0,406
366	St7	0,147	0,323	0,044
367	Itln1	0,134	0,000	0,224
368	Htra4	0,071	0,009	0,143
369	Pnpt1	0,006	0,013	0,740
370	Slc7a7	0,298	0,023	0,086
371	C1ql2	0,449	0,066	0,004
372	4921513D23Rik	0,042	0,288	0,973
373	Chrna5	0,025	0,421	0,008
374	Arg2	0,351	0,007	0,010
375	Vasp	0,019	0,969	0,018
376	Pttg1	0,846	0,026	0,042
377	Hoxb5	0,019	0,120	0,135
378	Sgk3	0,289	0,008	0,005
379	Olfr187	0,000	0,822	0,094
380	Rin3	0,030	0,612	0,133
381	Prkx	0,153	0,012	0,084
382	Hoxd13	0,013	0,836	0,032
383	Fhod3	0,401	0,048	0,254
384	Pfkp	0,942	0,051	0,033
385	1190002H23Rik	0,234	0,017	0,005
386	Gm3367	0,517	0,031	0,086
387	Osta	0,313	0,003	0,016
388	Olfr560	0,487	0,010	0,016
389	Tor1aip1	0,525	0,037	0,052
390	Tagap	0,766	0,054	0,031
391	Src	0,238	0,013	0,052
392	Pik3r5	0,822	0,001	0,001
393	Lnpep	0,220	0,001	0,008
394	Paqr5	0,010	0,047	0,001
395	Lrrc25	0,167	0,006	0,090
396	Sbno2	0,198	0,003	0,010
397	Fam160a1	0,038	0,059	0,250
398	Pilra	0,241	0,028	0,220
399	Gm10404	0,009	0,166	0,582
400	Ido1	0,000	0,068	0,087
401	Tmem161a	0,026	0,469	0,144
402	BC013712	0,257	0,004	0,003
403	Piwil4	0,067	0,002	0,011
404	Cebpb	0,772	0,004	0,004
405	Dbnl	0,012	0,003	0,209
406	Oas1f	0,174	0,724	0,037
407	Dgkh	0,895	0,009	0,025
408	1700047I17Rik2	0,041	0,568	0,062
409	Fosl2	0,705	0,005	0,001
410	1600014C10Rik	0,577	0,012	0,001
411	2400003C14Rik	0,328	0,022	0,028
412	Mmp28	0,044	0,426	0,070
413	Gm2701	0,767	0,140	0,035
414	5730528L13Rik	0,421	0,045	0,204
415	Col11a2	0,286	0,182	0,004
416	Fcrlb	0,739	0,072	0,007
417	Mcmbp	0,643	0,004	0,011
418	Itgb6	0,163	0,015	0,327
419	4933404M02Rik	0,073	0,045	0,498
420	Irak3	0,343	0,141	0,025
421	Tmem170b	0,326	0,030	0,067
422	A630012P03Rik	0,004	0,464	0,004
423	Tlr6	0,811	0,018	0,003
424	Prkag3	0,203	0,003	0,004
425	Wdr43	0,042	0,021	0,946
426	Slc2a1	0,257	0,425	0,018
427	Ifrd1	0,045	0,040	0,368
428	Bcl2l1	0,703	0,256	0,012
429	C1ra	0,036	0,136	0,002
430	A630001G21Rik	0,082	0,008	0,018
431	A530064D06Rik	0,036	0,205	0,440
432	3110003A17Rik	0,005	0,051	0,043
433	Psme2	0,012	0,215	0,409
434	Gm10414	0,348	0,037	0,131
435	D17Wsu92e	0,018	0,517	0,223
436	Fam133b	0,175	0,033	0,049
437	Sell	0,707	0,055	0,040
438	Ptprj	0,146	0,286	0,014
439	11. Sep	0,028	0,712	0,013
440	Wdr16	0,077	0,011	0,497
441	Gm5712	0,007	0,206	0,006
442	Golga3	0,020	0,984	0,014
443	Lnx1	0,009	0,786	0,004
444	Irak2	0,048	0,007	0,032
445	2810408A11Rik	0,352	0,198	0,012
446	Znfx1	0,906	0,009	0,010
447	Trim36	0,007	0,001	0,228
448	Ranbp2	0,427	0,004	0,004
449	Sbds	0,031	0,015	0,095
450	Pvr	0,006	0,434	0,309
451	2210403K04Rik	0,003	0,039	0,454
452	Snx2	0,488	0,019	0,027
453	2200002D01Rik	0,556	0,156	0,027
454	Casz1	0,313	0,010	0,009
455	Odz4	0,053	0,462	0,046
456	Gm12592	0,048	0,397	0,304
457	Ccdc50	0,064	0,049	0,401
458	Hck	0,631	0,018	0,011
459	Sypl2	0,018	0,967	0,015
460	Etv2	0,836	0,004	0,270
461	Yipf2	0,006	0,860	0,460
462	Wdr37	0,807	0,253	0,024
463	Gm5039	0,001	0,269	0,021
464	Ms4a8a	0,075	0,002	0,127
465	Rilpl1	0,161	0,030	0,230
466	Sprr2e	0,100	0,043	0,194
467	Arl5c	0,004	0,012	0,392
468	Ccl9	0,021	0,758	0,017
469	Rnf135	0,810	0,048	0,041
470	Trim12a	0,040	0,013	0,849
471	Dcun1d3	0,949	0,031	0,067
472	Zfp11	0,179	0,177	0,002
473	BC100530	0,105	0,004	0,527
474	Trim26	0,146	0,017	0,917
475	LOC100046147	0,030	0,847	0,032
476	Ccdc86	0,603	0,184	0,026
477	Tmtc2	0,079	0,038	0,873
478	Dck	0,003	0,797	0,012

supplemental figure S2

e1 2h treatment (mean)

	Gene	wt control	wt LPS	$MK2^{-/-}$ control	$MK2^{-/-}$ LPS	$MK2/3^{-/-}$ control	$MK2/3^{-/-}$ LPS
1	Ifnb1	100	712270	109	215270	128	313960
2	Cxcl1	100	132960	114	105320	83	38285
3	Il1a	100	126820	77	100910	110	75657
4	Cxcl10	100	74362	80	76084	78	108650
5	Cxcl2	100	48125	147	32917	147	11016
6	Cxcl3	100	42315	99	52950	81	19633
7	Il1b	100	39700	249	36601	229	30167
8	Csf2	100	20175	103	34292	112	9234
9	Ccl12	100	18702	70	12792	80	27858
10	Socs1	100	17857	118	16259	132	44103
11	Gem	100	11880	117	17087	76	25564
12	Il23a	100	10856	94	11421	101	3146
13	Mx2	100	10099	117	10310	123	17324
14	Gm13889	100	7054	111	17375	136	15627
15	Marcksl1	100	6722	133	6514	159	10340
16	Il2	100	6709	88	3748	94	804
17	Ccrl2	100	6403	133	8047	287	23266
18	Il10	100	5767	114	1999	125	620
19	Gm14047	100	5171	92	19004	152	11281
20	Slc7a11	100	4518	220	8599	124	5287
21	Sdc4	100	4426	271	7507	616	22667
22	Mab21l3	100	3954	74	7954	97	10636
23	4933432I03Rik	100	3580	185	5875	89	4540
24	Adora2b	100	3272	109	2005	91	955
25	Tcfec	100	3225	123	4460	124	4107
26	Tmem200b	100	3097	131	5748	108	2737
27	Mmp10	100	2971	45	1966	73	1144
28	Ednrb	100	2930	267	3879	173	1823
29	Il33	100	2897	103	4567	99	7631
30	Nod2	100	2777	143	2682	149	3344
31	Rel	100	2727	133	3328	101	2392
32	Serpinb2	100	2556	12	789	6	509
33	Oaf	100	2520	54	2130	39	1360
34	Flrt3	100	2475	232	2225	155	835
35	5730559C18Rik	100	2234	74	3396	76	2016
36	Nfkbie	100	2199	125	2250	99	1841
37	Ppp1r15a	100	1922	110	2136	132	3778
38	Fam55c	100	1892	120	3020	119	2261
39	Htra4	100	1828	278	4312	164	6410
40	Bcl2l15	100	1796	97	817	94	402
41	Etnk2	100	1738	93	786	112	756
42	Plekha4	100	1702	188	2484	98	4703
43	Nipal1	100	1696	161	1799	61	1206
44	Foxp4	100	1687	144	2891	108	3191
45	Tnip3	100	1641	123	2195	143	3230
46	Hamp	100	1618	81	1978	78	6435
47	Cd83	100	1607	166	2607	197	3981
48	Herc6	100	1603	62	492	89	1591
49	Klf7	100	1583	113	2384	105	2756
50	4933439G12Rik	100	1474	68	1757	39	936
51	Shbg	100	1426	118	728	199	412
52	Cav1	100	1421	166	1738	59	669
53	Fabp6	100	1401	61	1626	103	785
54	Pard6g	100	1298	59	1640	61	1085
55	Pydc3	100	1284	29	27	30	100
56	Plscr1	100	1234	147	1856	121	1734
57	Cdkn1a	100	1224	151	1375	117	2026
58	2500002B13Rik	100	1223	196	1479	85	550
59	Dusp16	100	1189	152	1021	143	2247
60	Adhfe1	100	1163	108	2223	139	1640
61	Plaur	100	1133	105	841	103	1178
62	Gpr4	100	1127	83	1233	100	667
63	Trim13	100	1104	113	962	94	676
64	3930401B19Rik	100	1068	83	1147	64	742
65	Csf3	100	1043	97	236	113	470
66	F930015N05Rik	100	1040	141	2169	78	3637
67	Cd44	100	1038	125	2032	171	2299
68	5830432E09Rik	100	891	98	800	66	409
69	Phldb1	100	842	110	1071	100	1098
70	Txk	100	837	211	625	121	447
71	Gpr132	100	827	163	995	142	1314
72	Plagl1	100	821	71	1186	57	2103
73	E230025N22Rik	100	818	78	775	120	444
74	Osgin2	100	814	98	671	107	589
75	Rnd3	100	758	294	815	232	1364
76	Lag3	100	751	99	828	109	3259
77	Cass4	100	734	102	1143	76	1184
78	Srgn	100	708	165	869	143	1020
79	F830208F22Rik	100	705	111	1122	82	1294
80	Slc16a10	100	684	133	532	122	444
81	1600029D21Rik	100	682	87	507	138	331
82	Nfil3	100	678	134	719	95	1742
83	Arid5a	100	676	140	910	172	2727
84	4930469K13Rik	100	674	141	1049	126	1714
85	Il1r1	100	663	155	426	54	292
86	Ier3	100	662	79	354	76	739
87	Tmem39a	100	661	92	909	90	1083
88	Ampd3	100	622	97	1125	85	980
89	Pgf	100	618	65	225	79	188
90	Tagap1	100	613	166	822	143	1742
91	Cd59b	100	585	47	101	51	252
92	Zswim4	100	576	121	744	146	1183
93	Fndc7	100	573	146	538	146	335
94	Nod1	100	573	66	102	103	732
95	Pcdh7	100	567	84	462	80	290
96	Pvrl2	100	551	125	810	108	1255
97	Ppap2b	100	541	90	416	85	195
98	Tmem27	100	537	100	1754	103	1416
99	Slc25a37	100	534	135	651	124	807
100	AW061096	100	531	56	399	49	190
101	Rffl	100	528	160	902	132	994
102	Rcor2	100	501	85	291	121	264
103	Ppm1k	100	498	59	206	77	505
104	Rcan1	100	497	108	574	132	713
105	Ifna12	100	486	72	177	52	315
106	Rbpj	100	479	111	554	132	857
107	Tagap	100	473	147	601	119	1237
108	Unc93a	100	466	394	757	189	578
109	Igsf9	100	463	103	731	198	1209
110	Itga5	100	462	82	499	102	554
111	A130078K24Rik	100	461	110	818	109	1085
112	Gm505	100	460	148	294	121	333
113	Eif4e	100	457	98	236	107	107
114	Dnajb2	100	449	110	729	109	681
115	1700109H08Rik	100	446	99	1078	88	866
116	Ifrd1	100	437	84	286	89	297
117	Rbm33	100	435	221	197	61	70
118	Dusp4	100	434	124	426	120	172
119	Pdpn	100	432	71	217	75	152
120	Tnfsf18	100	431	89	802	89	2127
121	Phxr4	100	429	161	1412	131	2399
122	Itpkc	100	426	108	531	113	980
123	Arg2	100	426	114	546	134	665
124	B3gnt2	100	425	108	451	109	824
125	Cdk12	100	423	131	563	122	897
126	Itpka	100	419	128	617	138	1041
127	4833422F24Rik	100	418	98	311	93	206
128	Mobkl2c	100	416	111	624	126	1067
129	Rasgef1b	100	414	124	593	168	673
130	Zfp296	100	414	109	394	95	831
131	Baz1a	100	411	114	409	96	519

e2 2h treatment (SEM)

	Gene	wt control	wt LPS	$MK2^{-/-}$ control	$MK2^{-/-}$ LPS	$MK2/3^{-/-}$ control	$MK2/3^{-/-}$ LPS
1	Ifnb1	5	181210	3	102570	26	89410
2	Cxcl1	40	12036	22	19300	42	6580
3	Il1a	27	19825	5	13752	25	11365
4	Cxcl10	7	8309	42	5030	23	10911
5	Cxcl2	33	4452	40	1648	67	2401
6	Cxcl3	111	10614	40	16199	109	2946
7	Il1b	33	2641	43	2430	94	1772
8	Csf2	3	4967	9	20071	13	2565
9	Ccl12	30	6030	12	2558	38	4961
10	Socs1	32	7118	25	1596	33	16521
11	Gem	38	2930	9	2992	36	3689
12	Il23a	3	3763	1	3789	3	387
13	Mx2	40	3217	33	1146	32	2903
14	Gm13889	14	1672	10	4507	18	3448
15	Marcksl1	22	1127	36	571	60	1040
16	Il2	18	2661	17	1434	29	548
17	Ccrl2	20	1326	11	979	7	3618
18	Il10	18	693	19	993	19	217
19	Gm14047	14	642	2	5832	59	2851
20	Slc7a11	76	993	66	647	66	661
21	Sdc4	11	793	194	4857	212	4367
22	Mab21l3	33	1050	10	2715	26	3704
23	4933432I03Rik	35	343	25	1009	34	518
24	Adora2b	29	275	9	88	21	148
25	Tcfec	6	214	26	481	9	558
26	Tmem200b	1	915	39	692	11	779
27	Mmp10	56	836	1	873	14	255
28	Ednrb	50	501	67	281	117	529
29	Il33	1	501	1	1281	1	3095
30	Nod2	10	351	26	146	35	166
31	Rel	14	339	7	231	15	166
32	Serpinb2	185	548	20	265	3	161
33	Oaf	55	567	17	621	17	115
34	Flrt3	79	964	46	352	70	220
35	5730559C18Rik	24	526	24	635	17	79
36	Nfkbie	38	94	27	204	30	63
37	Ppp1r15a	9	311	7	422	12	387
38	Fam55c	13	314	40	195	21	269
39	Htra4	12	442	192	294	49	1438
40	Bcl2l15	57	286	29	177	25	77
41	Etnk2	21	250	30	158	46	130
42	Plekha4	11	500	70	501	25	1476
43	Nipal1	85	443	197	225	27	112
44	Foxp4	32	291	105	267	10	632
45	Tnip3	3	251	23	389	15	265
46	Hamp	27	480	0	554	0	2008
47	Cd83	37	366	45	253	32	260
48	Herc6	6	710	5	124	4	600
49	Klf7	16	235	16	91	11	257
50	4933439G12Rik	35	247	54	289	10	129
51	Shbg	17	237	30	235	135	45
52	Cav1	64	619	84	431	19	151
53	Fabp6	15	158	18	919	34	123
54	Pard6g	51	336	8	83	21	112
55	Pydc3	67	1245	3	1	3	288
56	Plscr1	18	104	31	212	30	283
57	Cdkn1a	8	191	32	161	19	35
58	2500002B13Rik	16	217	131	563	22	107
59	Dusp16	18	268	19	89	44	476
60	Adhfe1	7	131	16	384	39	165
61	Plaur	42	72	12	77	24	118
62	Gpr4	21	216	24	152	76	124
63	Trim13	14	154	8	65	8	71
64	3930401B19Rik	40	161	18	130	6	111
65	Csf3	2	154	1	61	5	930
66	F930015N05Rik	9	326	28	780	16	1380
67	Cd44	11	117	21	341	34	178
68	5830432E09Rik	26	152	28	146	21	45
69	Phldb1	16	90	13	55	10	38
70	Txk	25	190	29	69	47	44
71	Gpr132	11	75	41	96	33	104
72	Plagl1	53	257	12	245	30	486
73	E230025N22Rik	21	76	44	126	49	60
74	Osgin2	7	87	9	63	14	50
75	Rnd3	24	207	37	113	75	188
76	Lag3	3	285	1	304	19	1534
77	Cass4	25	130	31	56	11	233
78	Srgn	7	83	36	62	17	67
79	F830208F22Rik	10	133	22	68	26	157
80	Slc16a10	9	87	21	51	12	16
81	1600029D21Rik	27	80	4	145	36	100
82	Nfil3	9	141	36	96	2	468
83	Arid5a	17	311	13	224	29	1274
84	4930469K13Rik	26	160	32	192	48	442
85	Il1r1	47	183	25	96	10	44
86	Ier3	33	220	14	91	8	75
87	Tmem39a	5	120	7	84	6	61
88	Ampd3	6	133	14	111	7	181
89	Pgf	26	189	28	30	5	36
90	Tagap1	28	120	29	150	52	487
91	Cd59b	36	391	6	53	6	291
92	Zswim4	6	121	14	126	28	164
93	Fndc7	14	38	45	130	10	44
94	Nod1	12	213	11	50	6	373
95	Pcdh7	13	33	24	49	10	36
96	Pvrl2	10	123	14	197	17	279
97	Ppap2b	59	124	24	41	17	14
98	Tmem27	0	100	2	1383	2	302
99	Slc25a37	16	121	15	40	14	49
100	AW061096	73	121	41	94	16	42
101	Rffl	11	107	17	26	11	102
102	Rcor2	32	58	22	59	17	44
103	Ppm1k	11	167	6	72	12	102
104	Rcan1	6	30	24	94	9	87
105	Ifna12	8	149	21	53	14	146
106	Rbpj	17	103	21	14	52	132
107	Tagap	17	109	19	68	7	333
108	Unc93a	6	93	448	47	54	40
109	Igsf9	17	73	45	110	37	121
110	Itga5	19	22	8	59	11	32
111	A130078K24Rik	18	123	17	128	10	250
112	Gm505	9	45	8	61	27	30
113	Eif4e	10	60	11	31	14	10
114	Dnajb2	15	44	26	103	24	151
115	1700109H08Rik	6	90	20	191	20	155
116	Ifrd1	13	48	13	60	12	22
117	Rbm33	26	124	231	26	17	31
118	Dusp4	37	119	45	66	78	60
119	Pdpn	63	123	26	95	19	53
120	Tnfsf18	13	65	2	188	2	1044
121	Phxr4	27	145	73	675	26	991
122	Itpkc	5	50	10	149	8	122
123	Arg2	35	47	30	43	55	46
124	B3gnt2	12	104	4	51	6	153
125	Cdk12	6	58	15	41	13	94
126	Itpka	18	112	11	73	9	212
127	4833422F24Rik	11	58	14	72	10	32
128	Mobkl2c	14	142	9	103	6	232
129	Rasgef1b	26	80	45	70	46	44
130	Zfp296	22	72	24	39	12	67
131	Baz1a	8	35	17	23	9	30

supplemental figure S2

f1

6h treatment (mean)

	Gene	wt control	wt LPS	MK2$^{-/-}$ control	MK2$^{-/-}$ LPS	MK2/3$^{-/-}$ control	MK2/3$^{-/-}$ LPS
1	Il19	100	36038	7	23185	7	6860
2	Il6	100	28044	7	31564	8	35526
3	Hdc	100	22861	21	23351	20	16644
4	Il12b	100	21413	10	37383	12	36838
5	Ifnb1	100	21006	111	2887	58	6048
6	Cxcl3	100	18329	57	49737	80	6566
7	Shisa3	100	16689	18	34964	23	10665
8	Cxcl1	100	16091	28	23606	51	8002
9	Cxcl9	100	15004	18	16035	23	64723
10	Cd40	100	13449	29	16470	41	18826
11	Cd69	100	12679	60	10203	58	23936
12	Serpina3f	100	12186	102	2004	99	17155
13	Csf3	100	12111	51	8567	47	1582
14	Saa2	100	10654	60	3218	48	202
15	Fpr2	100	10580	52	21804	75	19604
16	Socs3	100	10564	43	7866	50	7170
17	Iigp1	100	10352	15	7504	25	15657
18	Niacr1	100	9932	37	13666	58	9728
19	Il10	100	9297	48	3181	56	1799
20	Trim30c	100	9189	52	7674	35	6309
21	Lcn2	100	9156	91	6007	82	2182
22	Tnf	100	8651	70	7272	106	12592
23	Serpina3g	100	8612	47	4547	41	15764
24	Csf2	100	8532	88	26916	81	5676
25	Saa3	100	8488	44	8644	12	2660
26	Lhx2	100	8487	67	5838	52	15731
27	Mmp13	100	8462	37	11930	43	13569
28	Usp18	100	8370	50	5594	53	6877
29	AW112010	100	8024	84	7500	73	10432
30	Gbp2	100	7987	61	9050	68	10333
31	Saa1	100	7317	43	6662	52	2204
32	Slc28a2	100	7078	60	3825	83	4556
33	D730047E02Rik	100	6861	118	14731	107	11595
34	Socs1	100	6681	45	5616	84	3931
35	Gm14085	100	6384	61	3872	68	4579
36	Ptges	100	6333	68	9765	87	4012
37	Tnfsf10	100	6180	24	3649	28	6446
38	Mmp25	100	6011	30	5786	53	9430
39	Gm9706	100	6004	34	4046	74	6069
40	Ms4a4c	100	5951	58	9260	69	8670
41	Steap4	100	5930	95	1292	87	508
42	Il27	100	5483	129	3536	122	9386
43	Gm10522	100	5388	63	4589	59	802
44	Ifit2	100	5283	68	5002	100	7974
45	Oasl1	100	5235	33	5545	49	6129
46	Batf	100	4981	55	6523	54	4316
47	Cd200	100	4787	27	13858	57	15737
48	Isg15	100	4747	63	6448	64	6741
49	LOC100503637	100	4726	80	6623	78	5377
50	Pydc3	100	4551	8	161	25	573
51	Gm4951	100	4536	44	2932	65	7373
52	Ptx3	100	4526	28	3263	27	1297
53	LOC100503337	100	4370	79	5273	37	2765
54	Ccnd2	100	4302	27	6107	31	6738
55	Gbp4	100	4244	45	3186	49	5817
56	Tnfsf15	100	4170	62	10392	67	10550
57	Ms4a4b	100	4154	84	5810	77	7414
58	Ccl17	100	4146	81	9596	79	2455
59	Upp1	100	4139	86	4928	70	9006
60	Plat	100	4087	19	10969	12	7515
61	U90926	100	4065	6	6074	90	1403
62	Pfkfb3	100	4035	81	3173	60	411
63	Lipg	100	3917	68	1405	92	1794
64	Rnd1	100	3785	79	2845	48	373
65	Ifi47	100	3660	72	3816	94	4378
66	Herc6	100	3567	37	941	61	2089
67	Apol9b	100	3539	64	2280	53	1033
68	Il1f9	100	3464	52	1026	39	201
69	Adora2b	100	3374	47	2567	56	1619
70	Trim30b	100	3373	121	1848	69	1743
71	Enpp4	100	3294	65	2293	87	2154
72	Adhfe1	100	3087	121	5743	94	4145
73	Gch1	100	3050	67	2592	94	2613
74	Phf11	100	2989	56	2576	86	3730
75	Aqp9	100	2904	66	1780	57	472
76	Atp10a	100	2884	83	4972	37	4209
77	D14Ertd668e	100	2790	108	3788	103	4825
78	Ccrl2	100	2776	130	3419	173	7316
79	Lta	100	2773	82	4749	111	6781
80	Cish	100	2753	44	3548	9	1530
81	Plekha4	100	2749	48	2725	41	4133
82	Slc7a2	100	2692	63	3866	69	3973
83	Slamf7	100	2630	97	2383	111	3430
84	LOC100417829	100	2588	22	1761	45	3870
85	Slc7a11	100	2583	84	5031	112	1350
86	AA467197	100	2523	56	2798	81	3135
87	Il17rd	100	2492	17	2844	31	779
88	Ppef2	100	2481	125	2086	96	711
89	Serpinb2	100	2453	4	1389	4	255
90	4933432I03Rik	100	2331	62	4777	46	5081
91	Igtp	100	2311	65	1607	71	1400
92	Sdc4	100	2298	144	5950	742	16680
93	Fcgr2b	100	2198	142	2797	101	1184
94	Nod1	100	2197	49	913	64	1819
95	Fgl2	100	2154	84	1788	73	4151
96	BC006779	100	2003	85	2531	102	3339
97	1190003J15Rik	100	1992	101	1052	101	354
98	Btla	100	1977	143	6865	122	13424
99	A430084P05Rik	100	1946	60	2458	73	5616
100	Gm10851	100	1944	181	1113	115	363
101	1200009I06Rik	100	1939	66	2634	53	1785
102	Gm14047	100	1936	183	12259	101	7747
103	Clec4e	100	1932	109	3248	89	2416
104	Sele	100	1900	101	3552	109	17368
105	Ier3	100	1833	56	1585	46	865
106	Gbp9	100	1830	98	1613	83	2550
107	Gda	100	1819	51	2124	31	1094
108	Lag3	100	1794	102	2158	100	4472
109	Tiparp	100	1755	71	1464	97	956
110	Apol9a	100	1734	57	1171	51	581
111	Tslp	100	1733	75	3393	78	1039
112	Gnb4	100	1728	83	2179	82	3106
113	Arid5a	100	1699	96	1112	108	1531
114	Tnfaip6	100	1659	31	161	83	192
115	Kdm5b	100	1627	207	1040	176	2443
116	Rab11fip1	100	1626	80	2795	46	1609
117	Tmem2	100	1621	55	1091	66	799
118	Lrrc4	100	1613	75	1798	250	9276
119	Fpr-rs6	100	1601	29	840	147	2924
120	Ddx60	100	1596	22	739	77	1571
121	Il4ra	100	1589	88	621	67	203
122	Dusp16	100	1569	104	707	92	542
123	Il23r	100	1555	95	773	88	842
124	Trp53i11	100	1549	98	1745	64	1069
125	Hp	100	1495	51	477	66	153
126	Sytl3	100	1478	101	3345	130	7852
127	5730508B09Rik	100	1476	72	1233	102	1726
128	Nt5c3	100	1475	75	1185	92	1590
129	Tgtp1	100	1456	122	1054	189	2246
130	Fcgr4	100	1452	133	1412	148	2528
131	Ehd1	100	1450	75	2140	87	2014
132	Ddx58	100	1447	90	1211	93	1166
133	LOC668917	100	1441	74	1074	118	1259
134	Rhou	100	1441	11	681	18	92
135	Gm614	100	1407	141	1560	91	2749
136	H2-M2	100	1404	128	4104	291	2650
137	Rapgef2	100	1403	75	1633	108	2781
138	Hivep2	100	1398	55	531	72	395
139	Fgl1	100	1395	63	2615	135	3698
140	Gpr31c	100	1391	63	2306	81	3216
141	Dusp1	100	1369	86	926	152	886
142	Pdpn	100	1366	37	1799	41	512
143	Csprs	100	1351	56	715	52	1290
144	Nox1	100	1333	55	815	54	727
145	Tnfaip2	100	1332	133	1819	87	1006
146	S100a8	100	1312	63	400	144	148
147	Map3k8	100	1312	99	1058	102	781
148	Slc39a14	100	1297	52	1441	77	446
149	Nudt17	100	1296	27	2951	92	4464
150	Zc3h12a	100	1275	42	1121	86	958
151	I830077J02Rik	100	1273	69	503	58	794
152	Rtn1	100	1258	109	2498	108	1747
153	Ebi3	100	1258	99	1707	96	1668
154	LOC546061	100	1253	97	608	125	1404
155	Plagl2	100	1253	112	1264	104	714
156	Zeb1	100	1251	45	1167	61	608
157	Gbp11	100	1245	30	258	57	1486
158	Slc25a22	100	1233	42	751	125	1245
159	Cd247	100	1232	83	2114	82	1011
160	Atic	100	1219	67	1345	75	1967
161	Procr	100	1212	57	2105	85	3750
162	Fdxacb1	100	1201	60	823	150	1302
163	Flrt3	100	1189	63	1810	66	438
164	Tap1	100	1189	108	1133	96	1401
165	Irf7	100	1178	91	1488	72	1881
166	Ankrd33b	100	1174	50	1739	46	2350
167	Rcl1	100	1172	59	1342	72	724
168	Il12a	100	1158	101	2326	95	10255
169	Atp8a1	100	1150	69	797	139	1205
170	Xaf1	100	1148	119	1553	124	1456
171	Klf7	100	1144	71	1325	75	737
172	Golga7b	100	1143	73	684	101	996
173	Gm9883	100	1140	101	708	118	624
174	Nfkb2	100	1134	106	1618	87	1395
175	Mab21l3	100	1123	95	2742	117	7905
176	Pla2g4a	100	1107	43	824	80	830
177	A130040M12Rik	100	1093	150	1989	90	1796
178	Ext1	100	1093	55	1123	66	868
179	3930401B19Rik	100	1090	116	1634	69	1767
180	Fam55c	100	1075	58	2291	63	2345
181	1100001G20Rik	100	1074	93	550	112	254
182	Ripk2	100	1073	53	695	56	1226
183	Gm6907	100	1072	46	650	41	1140
184	Dgat2	100	1067	69	719	63	247
185	Tor1aip2	100	1065	83	929	80	1127
186	9130017N09Rik	100	1062	114	1043	202	467
187	Ddx4	100	1055	47	472	44	637
188	Bcl2l15	100	1051	95	398	117	253
189	Aldh1b1	100	1049	95	646	80	1369
190	Ttll2	100	1048	63	1370	78	3022
191	Naa25	100	1039	56	798	68	737
192	Mtmr7	100	1034	89	1382	97	1896
193	Magohb	100	1031	83	1048	105	751
194	Spint2	100	1029	48	1260	35	347
195	Art3	100	1024	67	381	63	625
196	Tcstv3	100	1016	61	844	66	1842
197	4932415M13Rik	100	1009	43	653	114	2645
198	Shbg	100	1009	165	526	142	348
199	Fst	100	1007	9	733	7	2657
200	Cx3cl1	100	1002	60	1024	80	237
201	Tmed5	100	998	88	435	83	946
202	Oas1g	100	998	49	547	72	618
203	Samsn1	100	997	84	1057	122	676
204	Slc6a4	100	997	125	644	94	296
205	Ms4a6b	100	995	73	1053	80	2097
206	Rel	100	994	21	421	66	1730
207	Gm4759	100	981	56	671	116	1434
208	Tmem171	100	980	135	895	133	1536
209	Nfil3	100	976	80	912	74	339
210	LOC100503357	100	975	82	544	149	968
211	Macc1	100	973	41	5415	44	3139
212	Gm10649	100	972	120	640	261	2986
213	Gm8369	100	962	45	953	54	1831
214	Sod2	100	954	95	1148	100	899
215	G630030J09Rik	100	947	95	410	100	116
216	9030625A04Rik	100	945	136	1789	114	2438
217	Ets2	100	944	65	1153	113	919
218	Olfr1417	100	937	119	671	116	1080
219	Zfp36	100	932	60	589	104	444
220	Mthfd2	100	932	52	574	64	422
221	Acsl1	100	929	104	1660	83	914
222	Gypc	100	928	112	855	77	1487
223	AA960436	100	925	99	714	89	712
224	Nlrp3	100	916	117	1461	116	1698
225	Trim21	100	911	68	887	82	626
226	AI447881	100	909	115	314	76	510
227	Rin2	100	900	93	1167	138	1865
228	Gm1966	100	899	149	1438	155	1664
229	Tmem67	100	896	78	712	75	927
230	Tnnt2	100	895	34	604	24	143
231	Unc93a	100	895	169	984	203	1921
232	Ikbke	100	892	110	1339	111	1507
233	Ambp	100	890	67	712	70	264
234	LOC236220	100	890	85	619	68	1349
235	Dusp5	100	889	73	957	49	2224
236	Parp14	100	884	87	1014	117	1094
237	Nmi	100	884	74	599	88	716
238	Gvin1	100	871	141	1223	178	1268
239	Gm10537	100	871	45	850	72	1361
240	Nrg4	100	868	62	534	81	384
241	Etnk2	100	852	137	235	121	218
242	Cd83	100	851	122	2300	183	3907
243	Rln3	100	850	74	760	70	1270
244	Clec2d	100	849	83	566	122	903
245	Mfsd7a	100	844	59	691	63	526
246	Trim12c	100	844	133	1092	101	994
247	Col27a1	100	842	99	1761	106	889
248	N4bp1	100	837	62	727	82	595
249	Txlnb	100	836	97	1353	100	1352
250	Aida	100	830	80	1054	85	1226
251	Etv6	100	827	86	861	103	1075
252	Hopx	100	825	133	1238	127	2425
253	Flnb	100	822	50	1094	43	1213
254	Iigp1b	100	822	121	139	95	829
255	Lass6	100	821	73	1244	83	1228
256	St3gal1	100	820	95	1082	113	888
257	D16Ertd472e	100	818	84	1033	57	502
258	Gm9789	100	813	195	498	169	1212
259	Tnfrsf1b	100	808	106	826	113	548
260	Papd7	100	797	92	802	116	1107
261	Ppfia3	100	792	110	1817	93	897
262	B430306N03Rik	100	788	62	353	75	661
263	Clic4	100	788	103	799	102	1005
264	Cebpd	100	782	91	420	125	161
265	Clcf1	100	780	83	1213	47	1893
266	Lrrc8c	100	780	93	939	81	817
267	Arhgef3	100	777	97	1233	83	1533
268	Fam49a	100	776	111	809	100	581
269	Stard7	100	775	74	771	100	569
270	Olfr832	100	772	171	335	207	770
271	4732419C18Rik	100	770	34	278	28	60
272	Elavl4	100	768	67	482	134	749
273	Areg	100	764	96	2386	90	801
274	Gm5483	100	762	63	199	47	73
275	Ccr9	100	758	167	1035	108	958
276	Tmem39a	100	744	52	1059	76	1909
277	Scube3	100	730	22	651	26	186
278	Stk40	100	726	107	702	86	425
279	Epb4.1l4b	100	724	87	493	113	214
280	Gja1	100	718	24	198	22	160
281	Tank	100	717	87	702	97	837
282	Ncoa7	100	716	107	629	77	1025
283	Casp7	100	714	80	832	82	587
284	Tyms	100	713	143	492	99	528
285	Pml	100	710	78	578	92	794
286	Gm5544	100	708	107	581	130	352
287	Ms4a6c	100	707	123	937	86	1014
288	Prl6a1	100	704	173	1046	114	1667
289	Stard5	100	703	67	569	71	467
290	B3gnt2	100	701	93	562	111	839
291	Prm1	100	699	150	465	189	1688
292	Calcrl	100	697	64	644	84	198
293	Rnf213	100	694	93	926	153	1141
294	Adora3	100	691	49	123	39	62
295	Ednrb	100	690	111	808	127	317
296	Pard6g	100	689	47	326	44	635
297	Alpk2	100	684	101	1110	95	486
298	Pax3	100	684	114	636	116	872
299	Fam82a2	100	684	86	662	89	987
300	Dtx2	100	682	62	607	73	418
301	Cd86	100	681	97	867	114	1351
302	Parp10	100	680	74	324	75	389
303	9930111J21Rik1	100	679	107	580	80	854
304	4930420K17Rik	100	679	110	821	131	985
305	Rbpms	100	677	59	524	57	386
306	Srgn	100	676	147	1016	152	1092
307	D2Bwg0886e	100	675	99	455	136	783
308	Tha1	100	673	83	378	62	168
309	Kcnn3	100	665	147	496	156	1085
310	Tbrg4	100	664	139	484	126	795
311	Lphn2	100	661	55	629	126	231
312	Osgin2	100	651	73	784	111	1057
313	Ccdc25	100	649	108	647	109	964
314	Zfp599	100	648	82	384	93	65
315	Gcnt2	100	644	45	226	30	113
316	BC147527	100	644	131	770	145	1292
317	Krtap16-10	100	643	168	522	154	1380
318	Rab20	100	642	115	1188	124	1349
319	Ppa1	100	641	59	468	59	707
320	Tpst1	100	640	21	246	100	399
321	Camta1	100	639	101	817	95	209
322	Bcl3	100	639	77	525	88	241
323	Ms4a6d	100	638	108	989	95	969
324	Gstt1	100	629	103	554	85	359
325	Pnp	100	626	106	691	91	682
326	Stat2	100	626	101	852	120	850
327	Tlr1	100	624	84	991	107	957
328	Kdm4a	100	623	84	1023	92	631
329	Lck	100	621	118	803	92	3541
330	Mcoln2	100	620	104	702	116	280
331	Snx10	100	620	116	529	86	495
332	Mtdh	100	619	111	939	100	817
333	Ggct	100	616	61	115	73	657
334	Prokr1	100	616	114	154	126	27
335	Gfpt2	100	615	162	272	193	241
336	Rnf19b	100	615	85	594	81	947
337	Gas7	100	613	126	910	105	389
338	Maff	100	613	36	1027	58	836
339	Fscn1	100	608	89	1380	46	879
340	Gna13	100	605	69	692	58	940
341	Glipr2	100	603	77	422	80	611
342	Wars	100	602	95	449	83	500
343	Flt1	100	598	98	369	99	184
344	Ppm1k	100	590	42	133	66	543
345	Spink2	100	581	73	236	69	313
346	Osm	100	579	108	969	95	1058
347	Tagap1	100	575	121	615	89	861
348	Pou3f1	100	573	152	566	147	1391
349	Slfn9	100	569	112	1273	120	1347
350	Phactr1	100	567	124	332	95	187
351	Pdss1	100	567	77	480	63	757
352	Gm10791	100	567	78	378	45	886
353	Sco1	100	566	61	536	70	976
354	Slamf8	100	565	138	1030	234	1342
355	Bco2	100	565	132	1137	69	1136
356	Trem3	100	560	43	464	30	151
357	1700054O13Rik	100	558	112	1832	105	1832
358	Carhsp1	100	556	93	443	91	723
359	Lsm14a	100	555	82	411	108	590
360	Tcfec	100	555	87	743	93	340
361	Aff1	100	553	27	297	31	193
362	Slc7a5	100	549	32	264	30	127
363	Zfp459	100	547	73	102	51	48
364	Ddit4	100	546	77	478	57	90
365	Insl6	100	546	97	411	82	368
366	St7	100	545	76	773	93	461
367	Itln1	100	543	170	240	98	143
368	Htra4	100	542	78	1546	75	3867
369	Pnpt1	100	541	69	383	94	370
370	Slc7a7	100	541	75	410	77	276
371	C1ql2	100	540	91	463	94	1059
372	4921513D23Rik	100	534	69	632	79	629
373	Chrna5	100	532	144	222	302	764
374	Arg2	100	532	67	316	31	61
375	Vasp	100	532	164	708	128	529
376	Pttg1	100	530	85	522	79	635
377	Hoxb5	100	526	97	228	55	358
378	Sgk3	100	526	102	492	92	379
379	Olfr187	100	524	47	174	114	457
380	Rin3	100	523	100	883	54	592
381	Prkx	100	522	122	661	119	832
382	Hoxd13	100	521	40	87	76	606
383	Fhod3	100	520	39	238	40	89
384	Pfkp	100	519	85	513	101	727
385	1190002H23Rik	100	514	60	420	58	1093
386	Gm3367	100	514	72	669	39	1434
387	Osta	100	512	90	340	91	124
388	Olfr560	100	509	102	600	125	1085
389	Tor1aip1	100	507	81	548	100	751
390	Tagap	100	505	97	526	108	861
391	Src	100	504	122	410	63	265
392	Pik3r5	100	500	138	512	125	236
393	Lnpep	100	499	97	603	112	969
394	Paqr5	100	498	91	326	107	819
395	Lrrc25	100	498	94	374	90	264
396	Sbno2	100	497	138	387	116	172
397	Fam160a1	100	496	38	128	105	221
398	Pilra	100	494	108	729	137	963
399	Gm10404	100	494	95	328	102	363
400	Ido1	100	491	87	88	163	179
401	Tmem161a	100	491	82	369	123	692
402	BC013712	100	487	106	400	101	771
403	Piwil4	100	487	99	278	72	88
404	Cebpb	100	484	93	467	134	224
405	Dbnl	100	484	127	590	93	633
406	Oas1f	100	483	61	379	98	517
407	Dgkh	100	482	104	491	144	758
408	1700047I17Rik2	100	481	116	667	85	407
409	Fosl2	100	480	80	442	104	190
410	1600014C10Rik	100	475	87	442	67	722
411	2400003C14Rik	100	475	134	435	94	347
412	Mmp28	100	475	124	215	123	376
413	Gm2701	100	472	69	428	70	794
414	5730528L13Rik	100	469	58	359	53	225
415	Col11a2	100	469	89	542	80	381
416	Fcrlb	100	468	211	507	137	224
417	Mcmbp	100	467	106	509	95	828
418	Itgb6	100	464	91	220	99	142
419	4933404M02Rik	100	464	117	203	113	149
420	Irak3	100	464	115	643	102	280
421	Tmem170b	100	459	79	365	64	269
422	A630012P03Rik	100	459	64	135	106	407
423	Tlr6	100	458	68	444	89	717
424	Prkag3	100	458	44	295	157	960
425	Wdr43	100	458	85	325	96	323
426	Slc2a1	100	457	74	642	98	379
427	Ifrd1	100	454	64	625	105	732
428	Bcl2l1	100	451	64	432	117	519
429	C1ra	100	451	90	245	57	592
430	A630001G21Rik	100	451	141	624	121	790
431	A530064D06Rik	100	450	37	147	33	223
432	3110003A17Rik	100	450	82	638	91	526
433	Psme2	100	449	118	559	93	518
434	Gm10414	100	448	256	304	74	167
435	D17Wsu92e	100	447	139	575	81	493
436	Fam133b	100	446	94	683	95	1052
437	Sell	100	446	140	522	157	1357
438	Ptprj	100	445	86	505	93	411
439	Sept11	100	444	72	635	48	423
440	Wdr16	100	442	322	754	349	903
441	Gm5712	100	441	108	112	151	299
442	Golga3	100	440	85	333	83	440
443	Lnx1	100	437	59	179	89	405
444	Irak2	100	437	97	568	99	692
445	2810408A11Rik	100	435	120	348	141	579
446	Znfx1	100	435	86	432	116	564
447	Trim36	100	435	82	1051	94	1302
448	Ranbp2	100	433	80	468	116	666
449	Sbds	100	432	81	719	108	1057
450	Pvr	100	432	69	735	75	553
451	2210403K04Rik	100	431	149	854	89	747
452	Snx2	100	431	101	459	111	586
453	2200002D01Rik	100	428	82	516	61	250
454	Casz1	100	427	42	621	39	2897
455	Odz4	100	425	133	100	177	296
456	Gm12592	100	425	112	187	167	297
457	Ccdc50	100	421	96	774	122	872
458	Hck	100	421	78	411	98	327
459	Sypl2	100	421	36	152	105	417
460	Etv2	100	421	97	328	95	103
461	Yipf2	100	420	108	271	97	382
462	Wdr37	100	420	90	397	101	538
463	Gm5039	100	419	14	87	71	260
464	Ms4a8a	100	418	120	144	80	82
465	Rilpl1	100	417	91	227	63	147
466	Sprr2e	100	417	260	681	341	899
467	Arl5c	100	415	125	1025	120	910
468	Ccl9	100	414	120	542	122	402
469	Rnf135	100	411	122	400	83	493
470	Trim12a	100	410	128	320	110	326
471	Dcun1d3	100	409	99	399	110	215
472	Zfp11	100	409	103	297	108	576
473	BC100530	100	407	29	117	40	84
474	Trim26	100	407	111	323	114	327
475	LOC100046147	100	403	81	203	84	389
476	Ccdc86	100	402	127	424	100	351
477	Tmtc2	100	401	73	1028	63	1100
478	Dck	100	401	58	260	115	416

supplemental figure S2

f2

6h treatment (SEM)

	Gene	wt control	wt LPS	MK2$^{-/-}$ control	MK2$^{-/-}$ LPS	MK2/3$^{-/-}$ control	MK2/3$^{-/-}$ LPS
1	Il19	212	18553	0	6002	0	3662
2	Il6	174	1588	2	1650	3	849
3	Hdc	102	3749	6	2342	2	459
4	Il12b	149	5607	2	3685	1	2511
5	Ifnb1	47	13024	36	1105	33	2346
6	Cxcl3	57	6948	22	20335	74	1559
7	Shisa3	141	1983	1	14200	7	3867
8	Cxcl1	31	1760	8	7023	23	2321
9	Cxcl9	134	2514	8	4029	6	6246
10	Cd40	82	2300	3	757	2	528
11	Cd69	66	1852	9	1435	15	665
12	Serpina3f	59	1146	40	473	48	2699
13	Csf3	67	3949	1	4584	0	848
14	Saa2	93	3768	12	637	0	129
15	Fpr2	74	3025	8	2660	46	1542
16	Socs3	75	243	8	498	8	97
17	Iigp1	82	1467	12	1239	20	1862
18	Niacr1	52	1598	7	645	16	1268
19	Il10	24	2162	4	1242	13	589
20	Trim30c	46	568	9	795	10	563
21	Lcn2	65	1389	11	1125	8	913
22	Tnf	21	4602	26	1331	17	1082
23	Serpina3g	47	927	6	851	4	659
24	Csf2	6	11176	5	10419	3	1414
25	Saa3	148	2061	5	1728	8	974
26	Lhx2	52	2136	9	1564	1	2212
27	Mmp13	97	690	6	875	7	1404
28	Usp18	49	104	15	523	31	99
29	AW112010	81	995	39	578	24	451
30	Gbp2	66	307	17	427	35	781
31	Saa1	114	1809	3	1565	9	979
32	Slc28a2	51	1134	16	253	17	457
33	D730047E02Rik	68	672	45	2119	51	2050
34	Socs1	52	578	13	539	11	551
35	Gm14085	46	517	14	224	10	389
36	Ptges	60	1600	23	1380	29	583
37	Tnfsf10	35	1192	17	622	18	1040
38	Mmp25	84	946	6	1109	34	345
39	Gm9706	51	147	4	176	35	617
40	Ms4a4c	48	803	38	1048	63	1457
41	Steap4	8	2029	2	298	0	361
42	Il27	20	1204	19	1006	5	597
43	Gm10522	30	1159	2	848	0	208
44	Ifit2	43	573	15	646	66	890
45	Oasl1	64	268	6	313	23	140
46	Batf	41	213	9	485	10	667
47	Cd200	68	383	16	2865	9	5276
48	Isg15	44	213	9	414	30	338
49	LOC100503637	30	608	7	424	11	362
50	Pydc3	38	725	2	36	9	488
51	Gm4951	50	1078	17	704	38	1505
52	Ptx3	118	2364	15	602	26	457
53	LOC100503337	82	990	15	826	8	317
54	Ccnd2	60	447	5	340	10	230
55	Gbp4	53	141	22	539	26	1210
56	Tnfsf15	34	517	8	2602	8	2523
57	Ms4a4b	36	354	32	215	51	1394
58	Ccl17	50	3459	16	5300	39	370
59	Upp1	42	486	4	508	3	379
60	Plat	82	544	9	1401	9	1313
61	U90926	62	1615	2	2425	2	408
62	Pfkfb3	41	789	8	704	9	132
63	Lipg	52	817	30	263	26	213
64	Rnd1	31	751	7	706	8	85
65	Ifi47	40	195	17	256	37	192
66	Herc6	11	704	5	171	7	238
67	Apol9b	39	464	3	387	9	125
68	Il1f9	25	2778	14	320	12	120
69	Adora2b	25	840	2	326	10	80
70	Trim30b	20	401	23	381	18	212
71	Enpp4	18	77	27	421	6	246
72	Adhfe1	8	401	37	842	6	533
73	Gch1	15	136	6	103	20	114
74	Phf11	36	363	9	211	26	345
75	Aqp9	51	1289	5	498	0	185
76	Atp10a	34	213	12	407	8	611
77	D14Ertd668e	35	98	9	337	37	451
78	Ccrl2	23	110	10	229	21	736
79	Lta	20	584	6	2137	98	526
80	Cish	41	605	10	732	4	236
81	Plekha4	27	255	11	422	6	515
82	Slc7a2	36	149	26	798	16	180
83	Slamf7	34	422	10	267	27	154
84	LOC100417829	32	289	4	183	16	1398
85	Slc7a11	61	262	28	926	50	99
86	AA467197	46	131	30	138	33	187
87	Il17rd	63	403	4	508	21	227
88	Ppef2	46	425	13	391	15	123
89	Serpinb2	125	1426	1	713	2	172
90	4933432I03Rik	31	592	28	910	9	722
91	Igtp	16	223	6	130	18	96
92	Sdc4	19	222	90	3180	352	3846
93	Fcgr2b	26	715	37	509	41	153
94	Nod1	18	154	3	134	3	106
95	Fgl2	27	804	22	330	20	503
96	BC006779	16	325	23	79	28	228
97	1190003J15Rik	14	583	12	124	9	102
98	Btla	64	545	90	4809	116	9063
99	A430084P05Rik	23	876	27	143	22	682
100	Gm10851	30	261	49	127	46	47
101	1200009I06Rik	11	384	8	258	7	198
102	Gm14047	9	86	188	7217	16	3083
103	Clec4e	37	147	26	370	56	292
104	Sele	4	1883	3	850	16	4988
105	Ier3	23	349	13	119	23	106
106	Gbp9	20	23	7	155	19	207
107	Gda	24	218	22	276	6	155
108	Lag3	10	110	19	1128	13	1424
109	Tiparp	11	172	11	248	21	169
110	Apol9a	25	97	2	216	8	125
111	Tslp	49	559	22	1009	44	312
112	Gnb4	23	137	11	217	19	255
113	Arid5a	25	66	6	150	35	168
114	Tnfaip6	76	123	9	113	29	111
115	Kdm5b	7	511	24	160	2	303
116	Rab11fip1	21	268	11	364	5	334
117	Tmem2	28	130	8	68	11	184
118	Lrrc4	27	912	31	951	283	1970
119	Fpr-rs6	40	499	16	160	11	401
120	Ddx60	9	347	13	77	11	468
121	Il4ra	14	643	5	115	1	25
122	Dusp16	8	86	12	152	7	104
123	Il23r	4	272	2	315	1	71
124	Trp53i11	23	275	24	96	14	198
125	Hp	29	835	18	121	7	48
126	Sytl3	69	1031	15	418	123	3399
127	5730508B09Rik	16	28	3	93	18	85
128	Nt5c3	14	168	9	119	10	39
129	Tgtp1	21	150	60	252	1	300
130	Fcgr4	26	83	50	130	58	419
131	Ehd1	26	85	22	72	20	128
132	Ddx58	8	20	16	33	21	44
133	LOC668917	16	21	12	84	1	607
134	Rhou	62	483	8	273	15	41
135	Gm614	22	695	42	252	5	139
136	H2-M2	60	375	37	796	488	1787
137	Rapgef2	5	364	13	215	9	309
138	Hivep2	13	170	12	206	7	128
139	Fgl1	43	197	30	1422	25	1308
140	Gpr31c	40	68	11	279	10	660
141	Dusp1	13	205	16	208	18	84
142	Pdpn	48	492	9	541	17	161
143	Csprs	17	250	20	72	14	292
144	Nox1	24	296	1	178	2	83
145	Tnfaip2	11	267	23	56	15	135
146	S100a8	42	702	16	187	112	92
147	Map3k8	12	76	11	155	15	70
148	Slc39a14	21	119	13	126	6	34
149	Nudt17	56	248	5	673	44	893
150	Zc3h12a	7	330	11	24	5	52
151	I830077J02Rik	12	149	44	55	12	196
152	Rtn1	18	182	32	485	42	137
153	Ebi3	26	56	9	70	2	133
154	LOC546061	10	142	9	72	16	105
155	Plagl2	16	122	7	103	2	81
156	Zeb1	28	175	20	58	2	164
157	Gbp11	30	140	18	70	20	476
158	Slc25a22	12	108	10	17	1	266
159	Cd247	13	141	18	503	37	116
160	Atic	18	186	8	290	16	98
161	Procr	36	422	10	421	32	710
162	Fdxacb1	14	53	10	65	6	593
163	Flrt3	59	476	8	467	33	211
164	Tap1	14	74	9	88	11	59
165	Irf7	35	81	23	116	26	177
166	Ankrd33b	17	321	11	166	5	55
167	Rcl1	13	147	7	132	3	107
168	Il12a	4	855	3	402	0	5441
169	Atp8a1	16	6	12	53	7	546
170	Xaf1	15	26	19	139	35	139
171	Klf7	15	162	6	151	5	150
172	Golga7b	16	20	6	71	13	583
173	Gm9883	8	128	7	222	43	28
174	Nfkb2	11	42	19	83	3	129
175	Mab21l3	21	249	14	591	7	115
176	Pla2g4a	17	92	7	83	10	46
177	A130040M12Rik	35	115	26	93	13	154
178	Ext1	24	107	9	39	2	67
179	3930401B19Rik	40	98	26	71	10	145
180	Fam55c	11	438	25	334	6	177
181	1100001G20Rik	35	604	17	262	38	67
182	Ripk2	26	292	5	133	6	131
183	Gm6907	13	94	9	102	3	32
184	Dgat2	27	344	3	173	1	29
185	Tor1aip2	4	55	7	71	12	26
186	9130017N09Rik	9	155	24	1190	238	129
187	Ddx4	14	207	15	85	4	147
188	Bcl2l15	6	504	3	202	29	97
189	Aldh1b1	15	101	9	141	3	201
190	Ttll2	22	139	18	450	27	916
191	Naa25	12	120	8	37	2	54
192	Mtmr7	19	188	15	73	22	238
193	Magohb	12	111	13	90	2	80
194	Spint2	13	133	17	440	1	64
195	Art3	24	40	2	103	0	141
196	Tcstv3	13	76	33	174	30	375
197	4932415M13Rik	6	238	20	52	13	521
198	Shbg	20	306	9	82	26	22
199	Fst	80	572	4	388	8	460
200	Cx3cl1	41	484	11	450	21	103
201	Tmed5	20	197	11	80	7	136
202	Oas1g	12	99	5	66	11	113
203	Samsn1	14	285	30	59	18	88
204	Slc6a4	19	13	15	178	7	44
205	Ms4a6b	20	341	22	219	31	233
206	Rel	13	214	7	87	11	661
207	Gm4759	18	150	5	114	21	312
208	Tmem171	17	352	24	128	19	160
209	Nfil3	5	112	24	132	2	85
210	LOC100503357	14	78	18	63	5	683
211	Macc1	44	972	63	1439	30	4607
212	Gm10649	20	432	46	189	139	306
213	Gm8369	16	139	36	91	20	330
214	Sod2	17	45	6	54	13	33
215	G630030J09Rik	5	211	3	202	11	30
216	9030625A04Rik	14	265	10	77	3	269
217	Ets2	34	127	20	58	24	17
218	Olfr1417	18	170	20	71	21	109
219	Zfp36	12	161	17	54	12	96
220	Mthfd2	34	169	21	114	31	96
221	Acsl1	9	99	7	305	8	247
222	Gypc	22	96	31	123	29	147
223	AA960436	8	46	2	62	7	50
224	Nlrp3	4	222	11	85	8	138
225	Trim21	8	29	8	112	12	74
226	AI447881	18	59	25	51	8	67
227	Rin2	19	196	19	214	57	114
228	Gm1966	14	82	25	138	56	143
229	Tmem67	14	56	17	45	7	50
230	Tnnt2	77	293	10	230	12	108
231	Unc93a	13	85	50	200	50	60
232	Ikbke	15	160	20	69	36	107
233	Ambp	21	255	21	103	18	82
234	LOC236220	17	31	26	109	14	295
235	Dusp5	47	361	14	170	34	233
236	Parp14	10	67	8	52	19	44
237	Nmi	6	40	10	64	13	65
238	Gvin1	20	69	32	91	40	173
239	Gm10537	9	183	11	112	3	111
240	Nrg4	27	229	17	71	8	85
241	Etnk2	16	213	8	23	10	29
242	Cd83	45	482	43	173	26	167
243	Rln3	16	159	7	127	5	106
244	Clec2d	9	212	17	78	10	87
245	Mfsd7a	9	110	15	128	4	49
246	Trim12c	12	21	18	44	22	52
247	Col27a1	26	138	21	317	17	146
248	N4bp1	9	85	10	65	11	40
249	Txlnb	35	115	10	334	15	133
250	Aida	7	43	14	89	10	68
251	Etv6	8	37	7	103	9	84
252	Hopx	16	346	29	265	19	351
253	Flnb	62	26	7	71	18	175
254	Iigp1b	4	127	28	41	0	383
255	Lass6	3	26	9	100	10	166
256	St3gal1	15	88	22	50	19	167
257	D16Ertd472e	23	209	17	174	5	76
258	Gm9789	14	159	17	102	29	137
259	Tnfrsf1b	12	37	6	48	16	26
260	Papd7	21	150	12	78	14	90
261	Ppfia3	7	70	11	237	11	195
262	B430306N03Rik	13	156	19	80	17	193
263	Clic4	10	23	9	57	9	17
264	Cebpd	15	80	16	63	23	34
265	Clcf1	39	197	10	130	1	364
266	Lrrc8c	18	56	10	19	11	57
267	Arhgef3	14	150	24	124	6	37
268	Fam49a	8	41	7	68	27	57
269	Stard7	17	182	14	62	9	45
270	Olfr832	4	296	78	78	31	191
271	4732419C18Rik	6	383	8	59	2	13
272	Elavl4	14	26	6	65	5	396
273	Areg	5	247	3	1168	0	64
274	Gm5483	52	511	15	159	0	25
275	Ccr9	10	83	11	71	30	89
276	Tmem39a	7	170	10	170	4	214
277	Scube3	57	190	7	204	27	74
278	Stk40	9	134	14	15	5	38
279	Epb4.1l4b	12	371	6	156	4	50
280	Gja1	81	216	10	66	16	34
281	Tank	11	41	4	44	10	29
282	Ncoa7	3	78	16	27	8	70
283	Casp7	15	57	17	67	8	64
284	Tyms	9	70	9	46	4	126
285	Pml	6	32	9	67	5	39
286	Gm5544	29	56	38	102	135	29
287	Ms4a6c	19	79	23	59	28	75
288	Prl6a1	14	61	9	180	28	107
289	Stard5	6	54	15	36	10	46
290	B3gnt2	13	44	9	54	18	39
291	Prm1	31	65	12	83	28	257
292	Calcrl	17	36	9	38	14	16
293	Rnf213	18	53	11	94	27	154
294	Adora3	16	404	8	54	5	32
295	Ednrb	27	313	25	118	33	26
296	Pard6g	31	51	9	48	14	131
297	Alpk2	4	103	3	172	0	52
298	Pax3	18	52	31	61	5	71
299	Fam82a2	11	67	15	89	3	57
300	Dtx2	7	30	3	70	4	66
301	Cd86	10	177	19	203	15	220
302	Parp10	21	36	11	39	9	33
303	9930111J21Rik1	9	67	14	72	11	40
304	4930420K17Rik	10	102	11	121	9	45
305	Rbpms	34	95	10	99	6	32
306	Srgn	14	22	29	27	20	59
307	D2Bwg0886e	13	23	1	46	7	266
308	Tha1	11	50	11	61	16	23
309	Kcnn3	15	173	8	54	17	15
310	Tbrg4	3	51	19	17	10	132
311	Lphn2	44	132	10	74	42	101
312	Osgin2	11	292	10	63	14	65
313	Ccdc25	8	111	16	95	6	64
314	Zfp599	28	132	17	376	44	1
315	Gcnt2	19	4	21	66	15	59
316	BC147527	9	97	24	109	27	140
317	Krtap16-10	16	121	14	18	11	391
318	Rab20	30	106	14	74	20	129
319	Ppa1	24	110	9	29	5	41
320	Tpst1	4	89	20	21	2	128
321	Camta1	4	308	3	140	0	80
322	Bcl3	9	58	10	65	10	50
323	Ms4a6d	4	43	26	82	25	141
324	Gstt1	9	70	13	110	21	35
325	Pnp	11	18	10	16	9	9
326	Stat2	10	86	18	83	25	15
327	Tlr1	20	84	14	58	50	145
328	Kdm4a	13	32	3	137	3	125
329	Lck	11	31	10	130	6	687
330	Mcoln2	18	226	15	145	34	50
331	Snx10	5	32	7	43	5	11
332	Mtdh	7	26	9	57	12	96
333	Ggct	12	47	15	10	3	48
334	Prokr1	10	242	54	57	19	0
335	Gfpt2	73	44	31	49	57	81
336	Rnf19b	9	106	6	156	2	78
337	Gas7	26	192	20	210	29	75
338	Maff	25	32	10	128	10	41
339	Fscn1	45	92	7	336	18	325
340	Gna13	12	99	7	126	6	85
341	Glipr2	40	44	9	15	18	104
342	Wars	9	43	3	28	4	64
343	Flt1	29	192	8	79	4	39
344	Ppm1k	11	138	13	32	6	71
345	Spink2	51	104	33	57	16	109
346	Osm	9	62	4	50	7	180
347	Tagap1	11	90	14	58	6	48
348	Pou3f1	8	223	14	201	8	224
349	Slfn9	28	38	22	149	14	313
350	Phactr1	20	73	31	59	37	44
351	Pdss1	11	70	5	57	6	34
352	Gm10791	29	208	27	29	9	126
353	Sco1	13	109	17	114	6	42
354	Slamf8	32	36	32	180	220	405
355	Bco2	9	67	11	102	6	191
356	Trem3	27	123	19	246	11	58
357	1700054O13Rik	13	30	16	535	6	596
358	Carhsp1	15	23	3	59	10	28
359	Lsm14a	10	3	5	40	6	203
360	Tcfec	11	177	24	148	20	82
361	Aff1	19	156	4	83	16	29
362	Slc7a5	63	187	27	101	40	64
363	Zfp459	35	353	19	51	17	5
364	Ddit4	54	167	11	65	18	21
365	Insl6	17	50	13	33	13	38
366	St7	15	40	7	139	5	58
367	Itln1	4	67	85	110	2	4
368	Htra4	23	79	33	705	30	1869
369	Pnpt1	3	20	7	23	2	31
370	Slc7a7	17	110	12	68	19	34
371	C1ql2	12	134	13	11	6	216
372	4921513D23Rik	4	19	10	30	9	76
373	Chrna5	20	166	55	32	139	257
374	Arg2	31	261	17	121	15	21
375	Vasp	12	43	19	33	9	43
376	Pttg1	12	12	5	33	0	30
377	Hoxb5	40	35	15	51	25	65
378	Sgk3	5	30	1	14	12	20
379	Olfr187	12	40	6	5	8	285
380	Rin3	8	43	19	129	3	121
381	Prkx	9	60	5	62	15	56
382	Hoxd13	19	75	8	42	15	474
383	Fhod3	40	330	12	210	20	51
384	Pfkp	9	67	10	57	12	53
385	1190002H23Rik	21	20	10	55	10	216
386	Gm3367	23	128	14	215	11	400
387	Osta	4	131	3	102	6	20
388	Olfr560	11	93	1	92	7	125
389	Tor1aip1	10	42	6	44	8	82
390	Tagap	14	65	18	41	4	148
391	Src	13	36	10	52	12	38
392	Pik3r5	13	24	20	44	21	23
393	Lnpep	5	29	13	68	10	59
394	Paqr5	11	56	6	16	7	125
395	Lrrc25	5	40	20	56	13	28
396	Sbno2	8	24	14	57	6	32
397	Fam160a1	21	140	6	57	24	54
398	Pilra	16	128	31	148	71	85
399	Gm10404	8	26	12	26	8	61
400	Ido1	16	85	2	2	161	75
401	Tmem161a	6	32	7	24	25	309
402	BC013712	5	29	15	52	16	51
403	Piwil4	13	11	11	62	6	25
404	Cebpb	6	23	17	46	19	31
405	Dbnl	6	15	12	22	10	22
406	Oas1f	10	85	8	23	15	54
407	Dgkh	2	9	28	59	4	73
408	1700047I17Rik2	14	67	8	26	12	96
409	Fosl2	15	105	12	49	10	18
410	1600014C10Rik	7	55	12	31	8	36
411	2400003C14Rik	8	36	10	21	1	23
412	Mmp28	6	118	9	46	4	67
413	Gm2701	7	155	7	80	21	128
414	5730528L13Rik	24	57	18	99	22	55
415	Col11a2	4	66	3	23	5	25
416	Fcrlb	29	142	23	16	48	50
417	Mcmbp	6	58	6	66	2	43
418	Itgb6	16	122	5	92	24	35
419	4933404M02Rik	8	106	17	63	33	58
420	Irak3	5	118	9	150	7	57
421	Tmem170b	6	96	14	46	2	19
422	A630012P03Rik	20	8	2	30	34	55
423	Tlr6	4	55	9	30	2	56
424	Prkag3	19	44	2	82	14	99
425	Wdr43	14	34	4	32	8	24
426	Slc2a1	18	123	11	102	11	27
427	Ifrd1	14	35	12	57	4	107
428	Bcl2l1	32	57	17	15	37	20
429	C1ra	6	70	12	40	8	49
430	A630001G21Rik	8	74	11	43	9	25
431	A530064D06Rik	12	71	10	54	9	108
432	3110003A17Rik	4	12	8	38	2	26
433	Psme2	9	20	5	22	12	42
434	Gm10414	44	137	45	88	8	43
435	D17Wsu92e	4	31	7	23	16	56
436	Fam133b	9	149	7	74	9	160
437	Sell	16	183	23	132	9	452
438	Ptprj	8	27	11	24	10	16
439	Sept11	26	38	5	53	9	37
440	Wdr16	32	20	63	167	87	145
441	Gm5712	10	127	24	24	48	44
442	Golga3	7	9	3	24	2	20
443	Lnx1	24	110	29	16	61	70
444	Irak2	6	46	4	28	6	37
445	2810408A11Rik	10	96	7	44	7	49
446	Znfx1	10	14	7	24	22	28
447	Trim36	14	72	13	145	15	128
448	Ranbp2	5	31	8	27	3	35
449	Sbds	6	75	19	68	6	198
450	Pvr	18	30	6	64	8	151
451	2210403K04Rik	11	49	11	64	11	120
452	Snx2	12	28	9	27	24	38
453	2200002D01Rik	42	135	5	89	15	52
454	Casz1	13	139	26	138	15	1221
455	Odz4	42	237	94	44	21	75
456	Gm12592	8	38	16	55	43	113
457	Ccdc50	7	144	32	50	13	107
458	Hck	3	17	8	13	8	18
459	Sypl2	6	43	15	41	17	82
460	Etv2	8	124	13	516	19	16
461	Yipf2	10	21	10	20	9	204
462	Wdr37	5	106	9	40	0	18
463	Gm5039	11	45	7	14	14	97
464	Ms4a8a	11	170	34	54	1	1
465	Rilpl1	8	137	12	62	6	35
466	Sprr2e	19	121	17	72	54	157
467	Arl5c	25	89	19	76	10	103
468	Ccl9	17	17	26	35	42	28
469	Rnf135	5	29	8	30	2	18
470	Trim12a	8	10	10	24	10	16
471	Dcun1d3	9	115	5	124	27	12
472	Zfp11	4	102	4	23	7	58
473	BC100530	30	174	12	70	11	9
474	Trim26	10	29	8	34	4	6
475	LOC100046147	5	29	16	42	13	59
476	Ccdc86	11	38	18	23	10	13
477	Tmtc2	7	112	24	384	21	310
478	Dck	16	5	18	17	3	50

supplemental figure S2

g

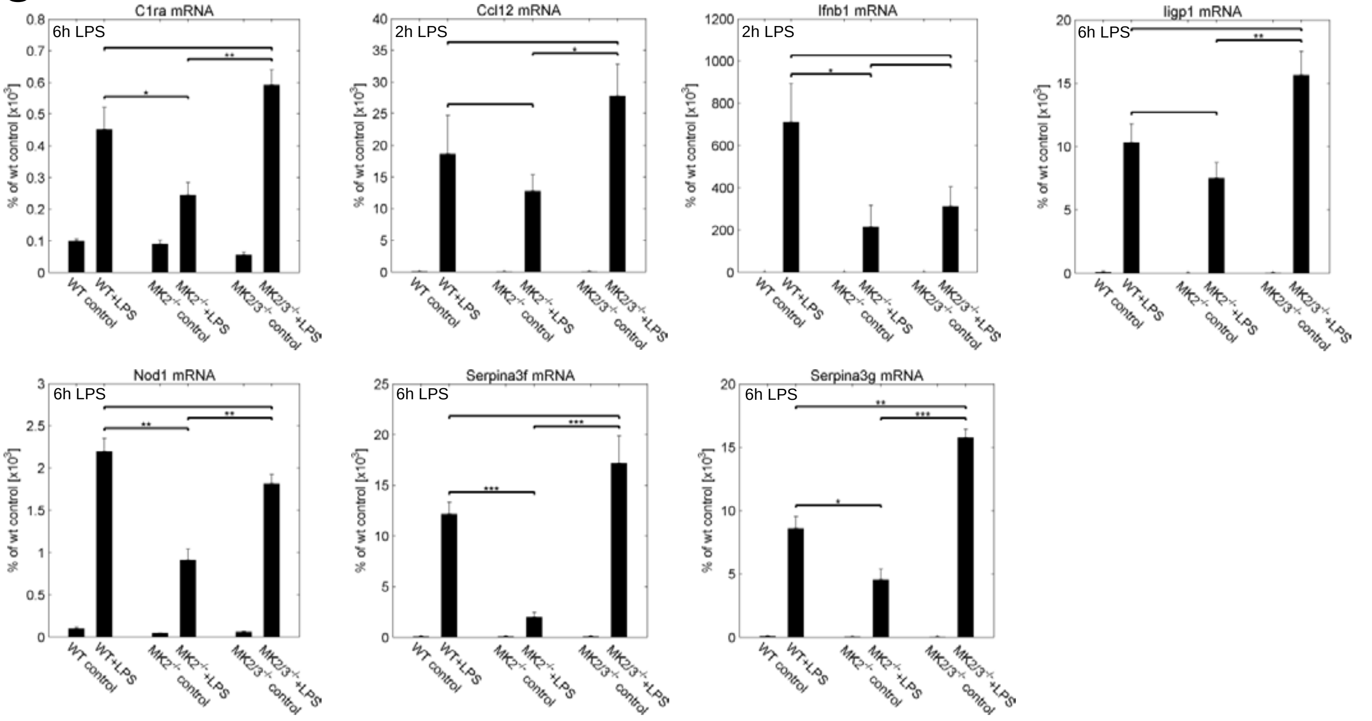

h

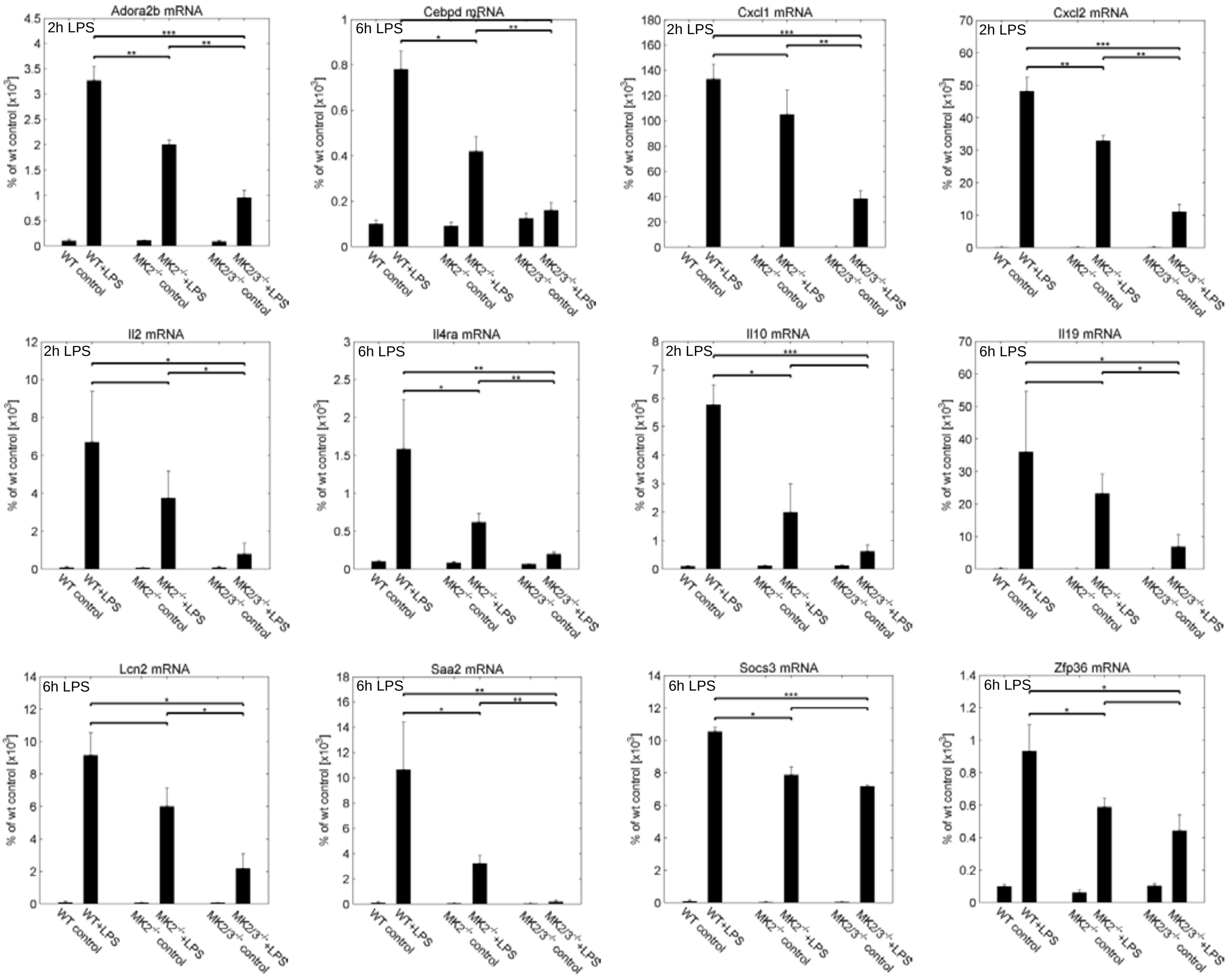

supplemental figure S2

i

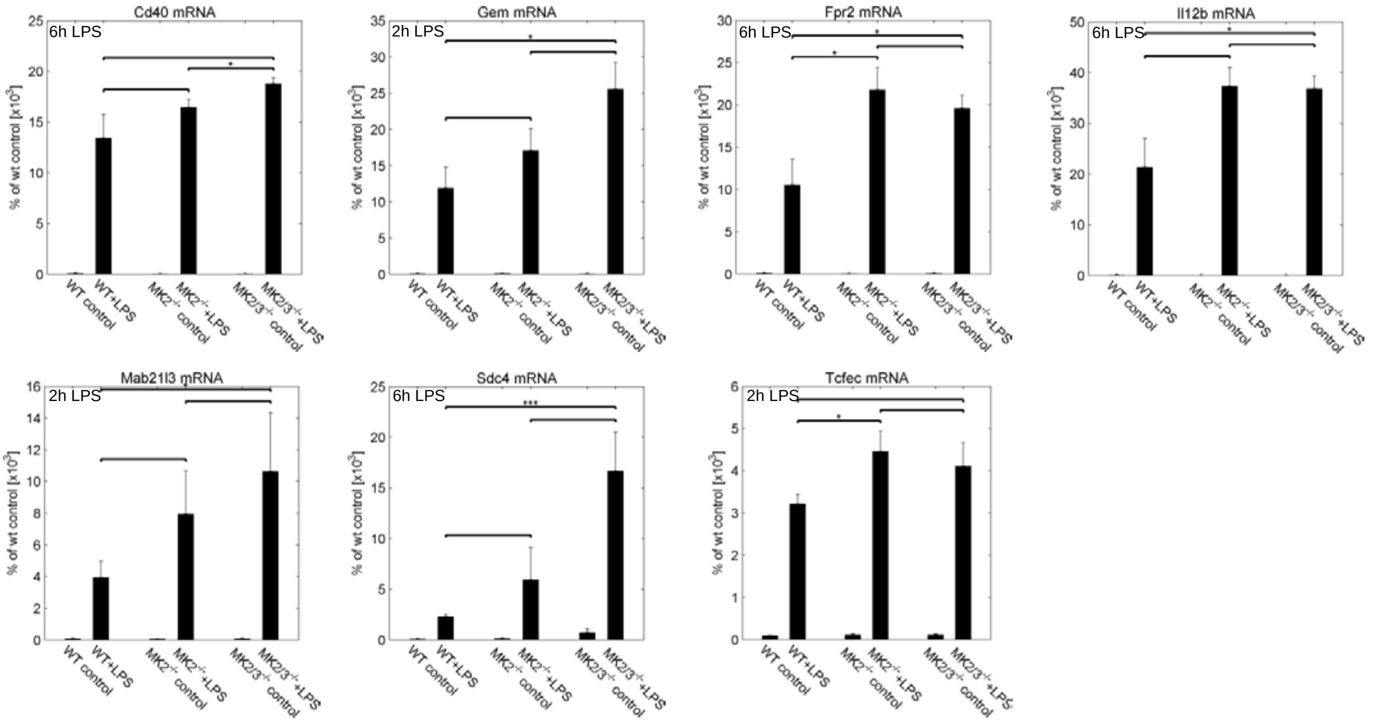

j

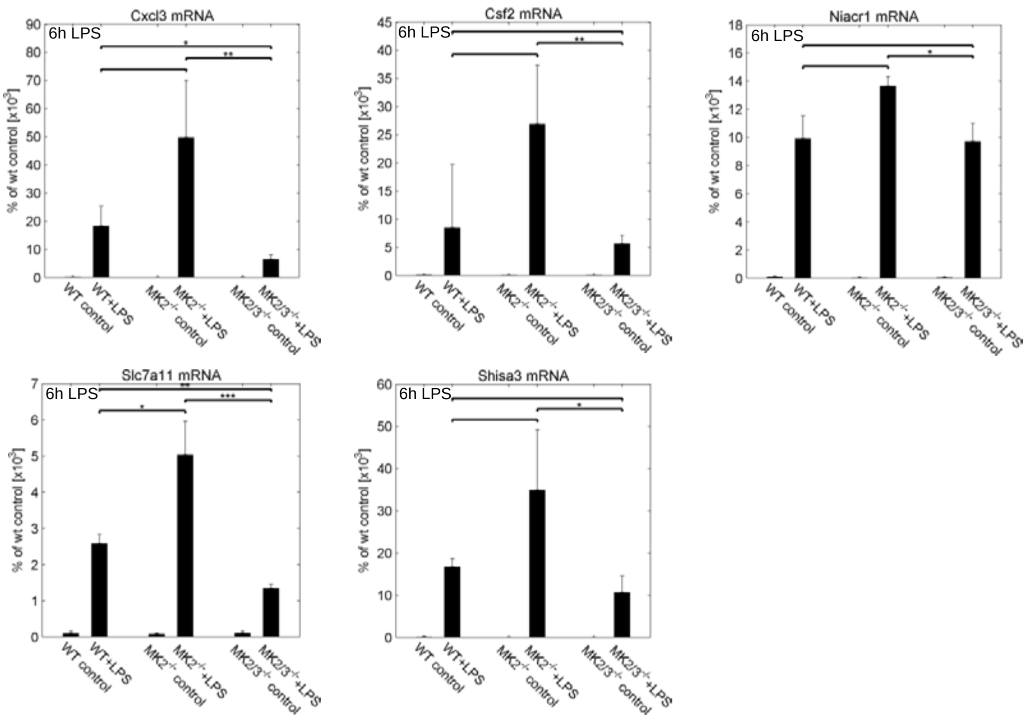

supplemental figure S2

k

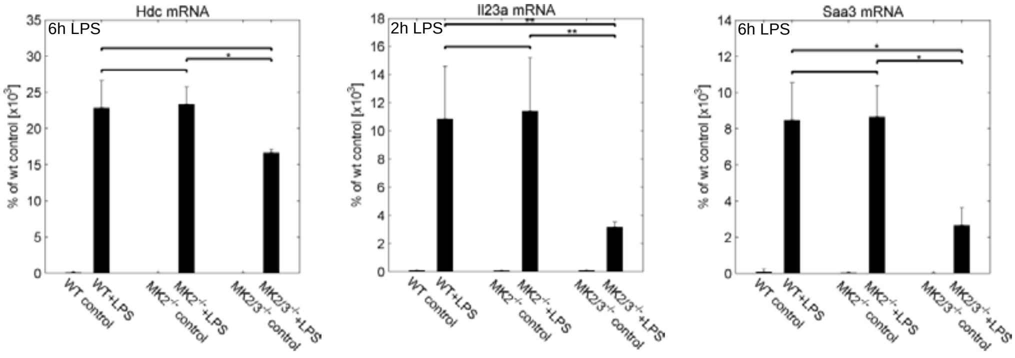

l

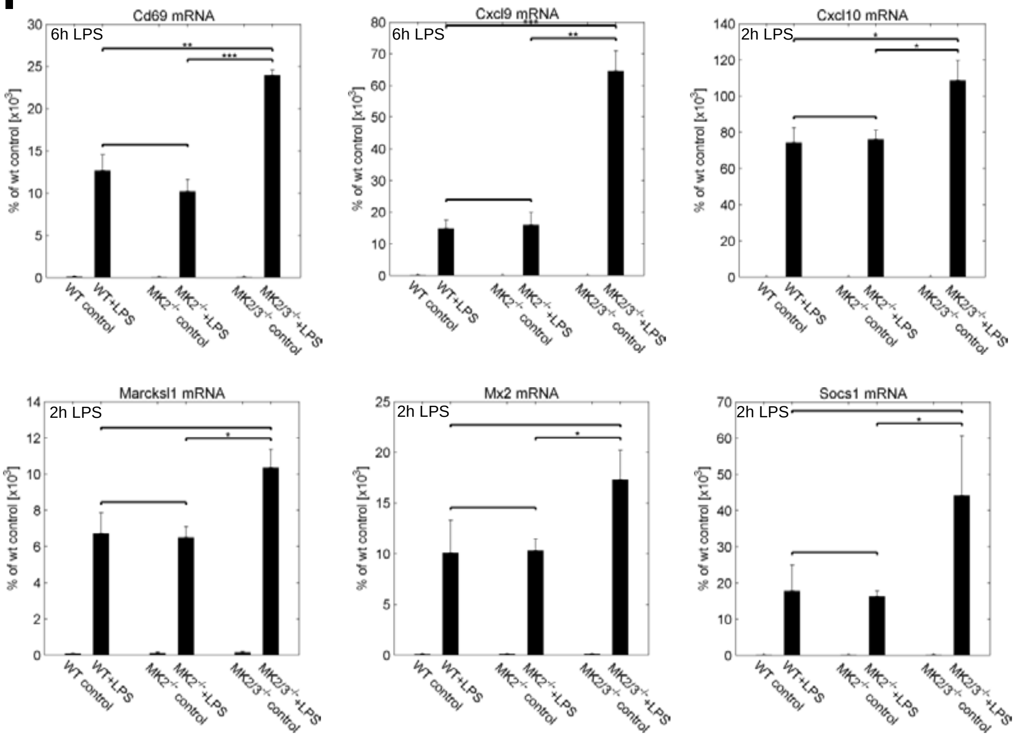

supplemental figure S2

a 2h LPS

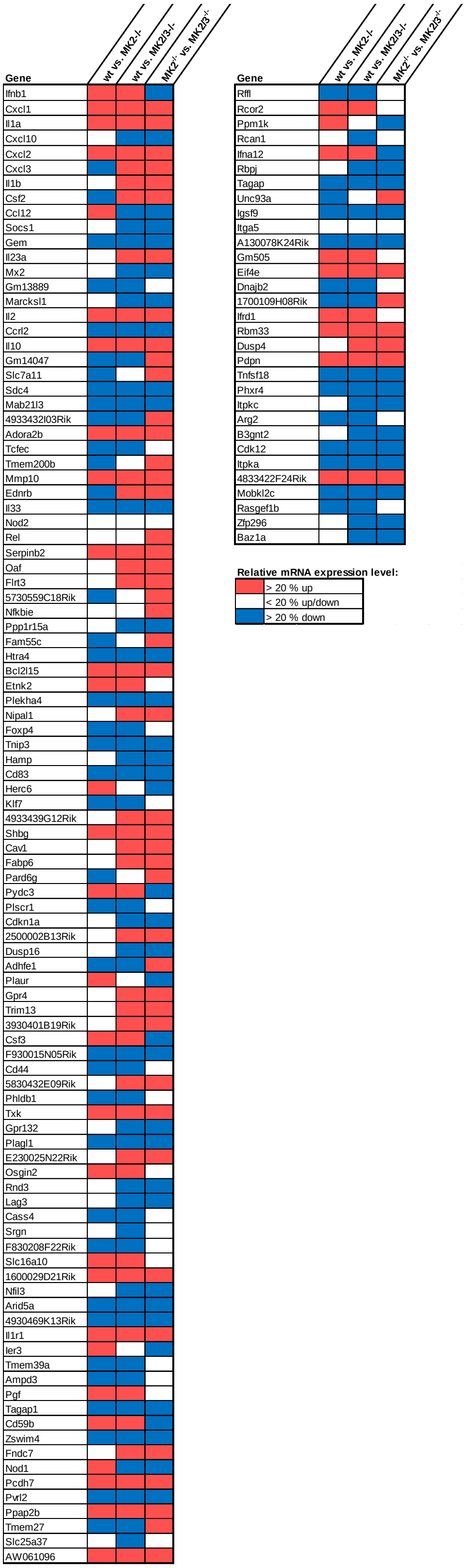

supplemental figure S3

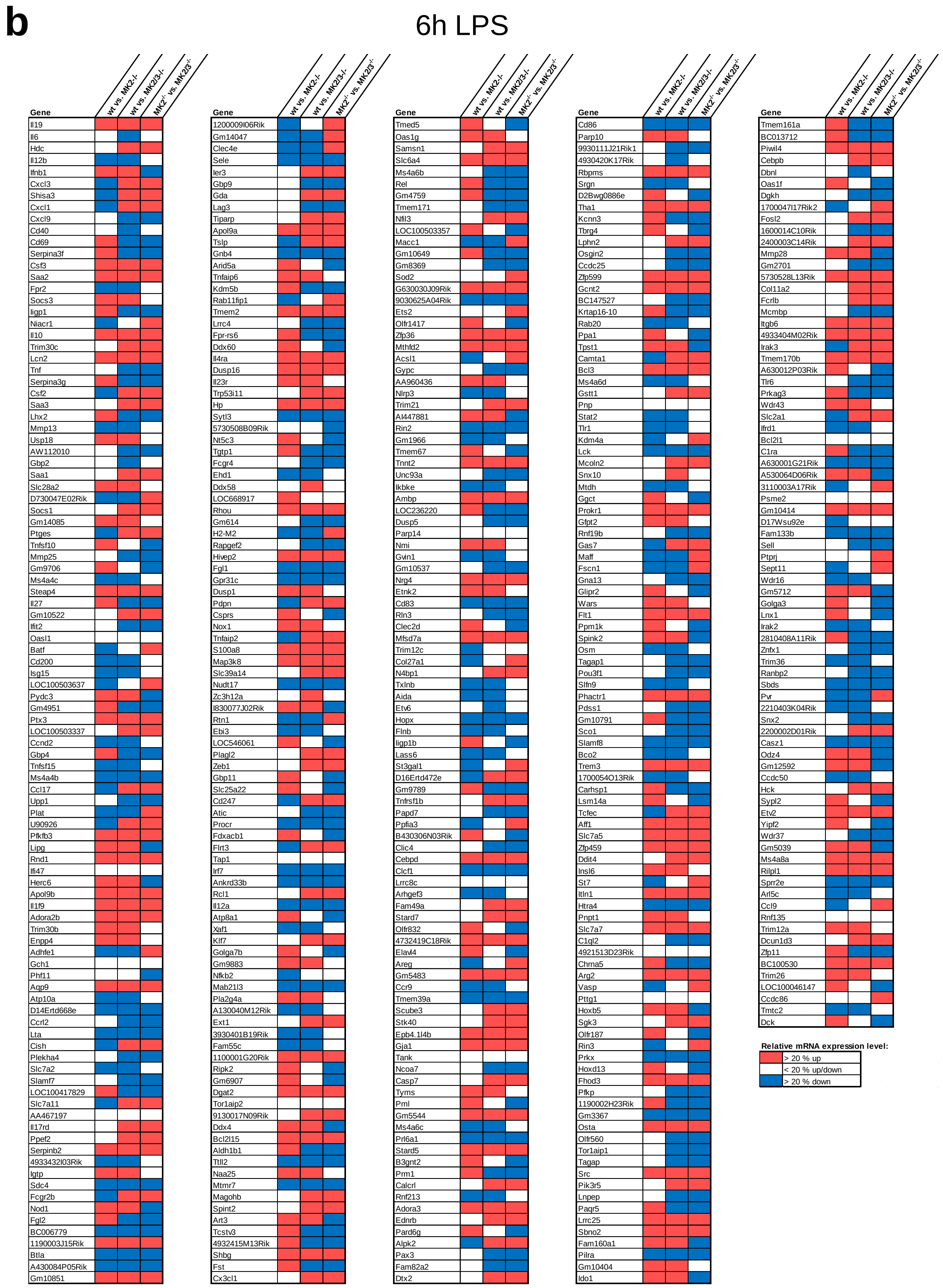

supplemental figure S3

a

2h LPS

	group I	group II	group III	group IV	group V	group VI
	**Il10-type**	**Il12b-type**	**Ifnb1-type**	**Cxcl3-type**	**Hdc-type**	**Cxcl9-type**
1	Cxcl1	Gem	Ifnb1	Cxcl3	Il1b	Cxcl10
2	Il1a	Gm13889	Ccl12	Csf2	Il23a	Socs1
3	Cxcl2	Ccrl2	Herc6	Gm14047	Oaf	Mx2
4	Il2	Sdc4	Pydc3	Slc7a11	Flrt3	Marcksl1
5	Il10	Mab21l3	Plaur	4933432I03Rik	Nipal1	Ppp1r15a
6	Adora2b	Tcfec	Csf3	Tmem200b	4933439G12Rik	Hamp
7	Mmp10	Il33	Ier3	Ednrb	Cav1	Cdkn1a
8	Serpinb2	Htra4	Cd59b	5730559C18Rik	Fabp6	Dusp16
9	Bcl2l15	Plekha4	Nod1	Fam55c	2500002B13Rik	Gpr132
10	Etnk2	Foxp4	Ppm1k	Pard6g	Gpr4	Rnd3
11	Shbg	Tnip3	Ifna12	Adhfe1	Trim13	Lag3
12	Txk	Cd83		Tmem27	3930401B19Rik	Nfil3
13	Osgin2	Klf7		Unc93a	5830432E09Rik	Rbpj
14	Slc16a10	Plscr1		1700109H08Rik	E230025N22Rik	Itpkc
15	1600029D21Rik	F930015N05Rik			Fndc7	B3gnt2
16	Il1r1	Cd44			Dusp4	Zfp296
17	Pgf	Phldb1				Baz1a
18	Pcdh7	Plagl1				
19	Ppap2b	Cass4				
20	AW061096	F830208F22Rik				
21	Rcor2	Arid5a				
22	Gm505	4930469K13Rik				
23	Eif4e	Tmem39a				
24	Ifrd1	Ampd3				
25	Rbm33	Tagap1				
26	Pdpn	Zswim4				
27	4833422F24Rik	Pvrl2				
28		Rffl				
29		Tagap				
30		Igsf9				
31		A130078K24Rik				
32		Dnajb2				
33		Tnfsf18				
34		Phxr4				
35		Arg2				
36		Cdk12				
37		Itpka				
38		Mobkl2c				
39		Rasgef1b				

supplemental figure S4

b

6h LPS

	group I<br>Il10-type	group II<br>Il12b-type	group III<br>Ifnb1-type	group IV<br>Cxcl3-type	group V<br>Hdc-type	group VI<br>Cxcl9-type
1	Il19	Il12b	Ifnb1	Cxcl3	Hdc	Cxcl9
2	Csf3	Fpr2	Cd69	Shisa3	Trim30c	Tnf
3	Saa2	Mmp13	Serpina3f	Cxcl1	Saa3	AW112010
4	Socs3	Ms4a4c	Iigp1	Niacr1	Saa1	Mmp25
5	Il10	Cd200	Serpina3g	Csf2	Socs1	Ifit2
6	Lcn2	Isg15	Lhx2	D730047E02Rik	Gm10522	Upp1
7	Usp18	Ccnd2	Tnfsf10	Ptges	LOC100503337	Ccrl2
8	Slc28a2	Tnfsf15	Gm9706	Batf	Il17rd	Plekha4
9	Gm14085	Ms4a4b	Il27	LOC100503637	Ppef2	Slamf7
10	Steap4	Atp10a	Pydc3	Ccl17	Ier3	Gbp9
11	Ptx3	D14Ertd668e	Gm4951	Plat	Gda	Lag3
12	Pfkfb3	Lta	Gbp4	U90926	Tiparp	Lrrc4
13	Rnd1	Slc7a2	Lipg	Adhfe1	Trp53i11	Fcgr4
14	Apol9b	4933432I03Rik	Herc6	Cish	Slc39a14	Gm614
15	Il1f9	Sdc4	LOC100417829	Slc7a11	Plagl2	Rapgef2
16	Adora2b	BC006779	Nod1	Fcgr2b	Zeb1	Atic
17	Trim30b	Btla	Fgl2	1200009I06Rik	Rcl1	Ms4a6b
18	Enpp4	A430084P05Rik	Arid5a	Gm14047	Klf7	Tmem171
19	Aqp9	Sele	Kdm5b	Clec4e	Ext1	Gm8369
20	Serpinb2	Gnb4	Fpr-rs6	Tslp	9130017N09Rik	Gypc
21	Igtp	Sytl3	Ddx60	Rab11fip1	Magohb	Unc93a
22	1190003J15Rik	Ehd1	Nt5c3	H2-M2	Spint2	Dusp5
23	Gm10851	Fgl1	Tgtp1	Pdpn	Cx3cl1	Gm10537
24	Apol9a	Gpr31c	Csprs	Tnfaip2	Samsn1	Rln3
25	Tnfaip6	Nudt17	I830077J02Rik	Rtn1	Nfil3	Papd7
26	Tmem2	Ebi3	LOC546061	Cd247	Trim21	Clic4
27	Il4ra	Procr	Gbp11	Flrt3	N4bp1	Ncoa7
28	Dusp16	Irf7	Slc25a22	Macc1	Tnfrsf1b	Pax3
29	Il23r	Ankrd33b	Fdxacb1	Acsl1	Fam49a	Fam82a2
30	Hp	Il12a	Atp8a1	Col27a1	Stard7	9930111J21Rik1
31	Rhou	Xaf1	Golga7b	St3gal1	Scube3	Osgin2
32	Hivep2	Mab21l3	Ripk2	D16Ertd472e	Stk40	Ccdc25
33	Dusp1	A130040M12Rik	Gm6907	Ppfia3	Casp7	BC147527
34	Nox1	3930401B19Rik	Ddx4	Areg	Calcrl	Rnf19b
35	S100a8	Fam55c	Aldh1b1	Alpk2	Ednrb	Gna13
36	Map3k8	Ttll2	Art3	Camta1	Dtx2	Tagap1
37	Gm9883	Mtmr7	Tcstv3	Kdm4a	Lphn2	Pou3f1
38	Pla2g4a	9030625A04Rik	4932415M13Rik	Gas7	Gstt1	Pdss1
39	1100001G20Rik	Nlrp3	Fst	Maff	Mcoln2	Sco1
40	Dgat2	Rin2	Tmed5	Fscn1	Ddit4	C1ql2
41	Bcl2l15	Gm1966	Rel	Tcfec	Sgk3	Pfkp
42	Naa25	Ikbke	Gm4759	St7	Pik3r5	Olfr560
43	Shbg	Gvin1	LOC100503357	Vasp	Cebpb	Tor1aip1
44	Oas1g	Cd83	Gm10649	Rin3	Fosl2	Tagap
45	Slc6a4	Txlnb	Olfr1417	1700047I17Rik2	2400003C14Rik	Lnpep
46	G630030J09Rik	Aida	AI447881	Irak3	Col11a2	Dgkh
47	Zfp36	Hopx	Tmem67	Slc2a1	Fcrlb	1600014C10Rik
48	Mthfd2	Flnb	LOC236220	3110003A17Rik	2200002D01Rik	Gm2701
49	AA960436	Lass6	Clec2d	Sept11	Hck	Mcmbp
50	Tnnt2	Clcf1	Iigp1b	Pvr	Dcun1d3	Tlr6
51	Ambp	Arhgef3	Gm9789	Ccl9		Sell
52	Nmi	Ccr9	B430306N03Rik			Znfx1
53	Nrg4	Tmem39a	Olfr832			Ranbp2
54	Etnk2	Ms4a6c	Elavl4			Snx2
55	Mfsd7a	Prl6a1	Pml			Wdr37
56	Cebpd	Rnf213	B3gnt2			
57	4732419C18Rik	Cd86	Prm1			
58	Gm5483	Srgn	Pard6g			
59	Epb4.1l4b	Rab20	D2Bwg0886e			
60	Gja1	Ms4a6d	Kcnn3			
61	Tyms	Stat2	Tbrg4			
62	Gm5544	Tlr1	Krtap16-10			
63	Stard5	Lck	Ppa1			
64	Adora3	Mtdh	Tpst1			
65	Parp10	Osm	Ggct			
66	Rbpms	Slfn9	Glipr2			
67	Tha1	Slamf8	Ppm1k			
68	Zfp599	Bco2	Spink2			
69	Gcnt2	1700054O13Rik	Gm10791			
70	Bcl3	Htra4	Carhsp1			
71	Prokr1	Prkx	Lsm14a			
72	Gfpt2	Gm3367	Chrna5			
73	Wars	Pilra	Hoxb5			
74	Flt1	Ifrd1	Olfr187			
75	Phactr1	A630001G21Rik	Hoxd13			
76	Trem3	Fam133b	1190002H23Rik			
77	Aff1	Wdr16	Paqr5			
78	Slc7a5	Irak2	Fam160a1			
79	Zfp459	Trim36	Ido1			
80	Insl6	Sbds	Tmem161a			
81	Itln1	2210403K04Rik	BC013712			
82	Pnpt1	Casz1	Oas1f			
83	Slc7a7	Ccdc50	Mmp28			
84	Arg2	Sprr2e	A630012P03Rik			
85	Fhod3	Arl5c	Prkag3			
86	Osta	Tmtc2	C1ra			
87	Src		A530064D06Rik			
88	Lrrc25		Gm5712			
89	Sbno2		Golga3			
90	Gm10404		Lnx1			
91	Piwil4		2810408A11Rik			
92	5730528L13Rik		Odz4			
93	Itgb6		Gm12592			
94	4933404M02Rik		Sypl2			
95	Tmem170b		Yipf2			
96	Wdr43		Gm5039			
97	Gm10414		Zfp11			
98	Etv2		LOC100046147			
99	Ms4a8a		Dck			
100	Rilpl1					
101	Trim12a					
102	BC100530					
103	Trim26					

supplemental figure S4

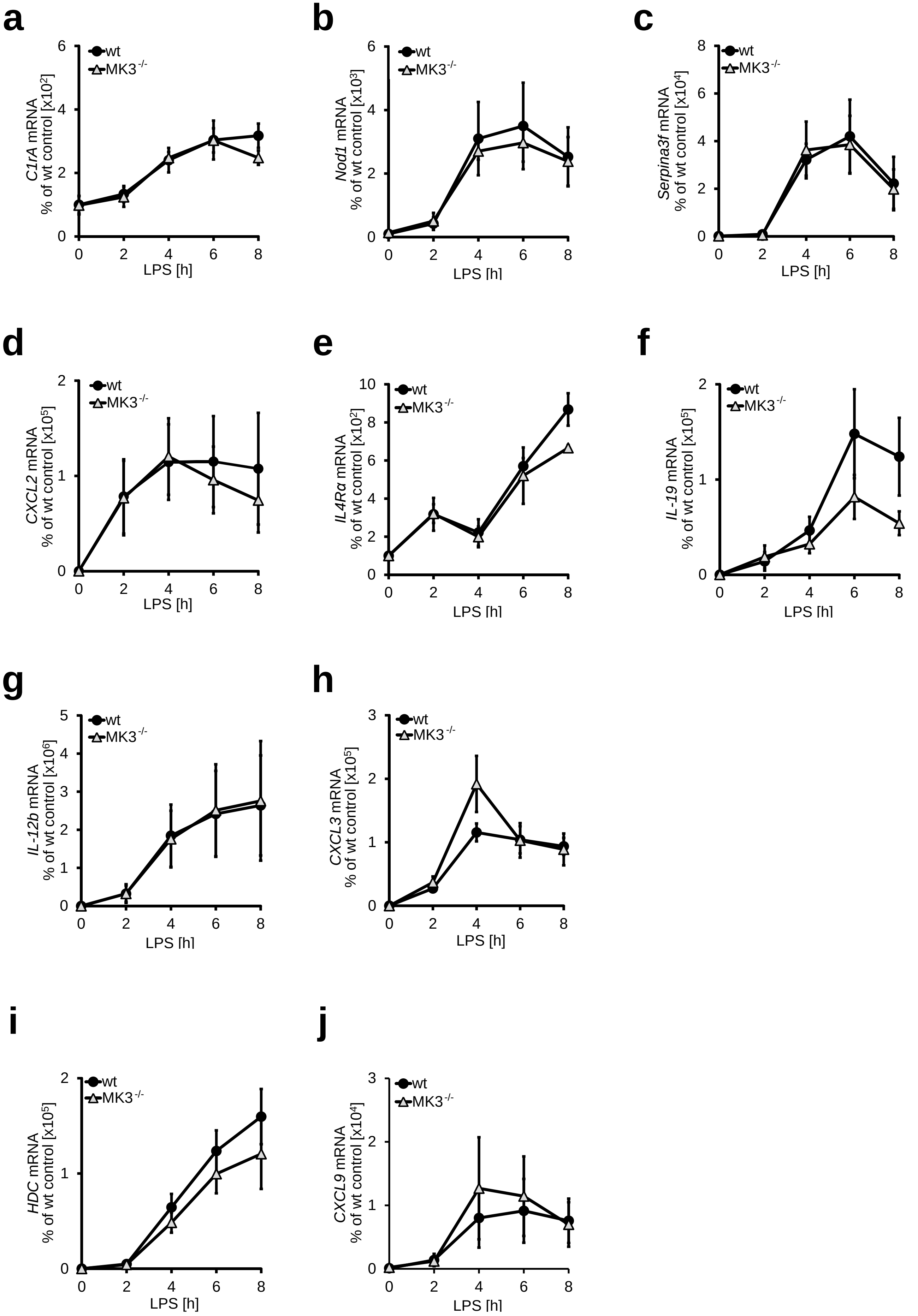

supplemental figure S5

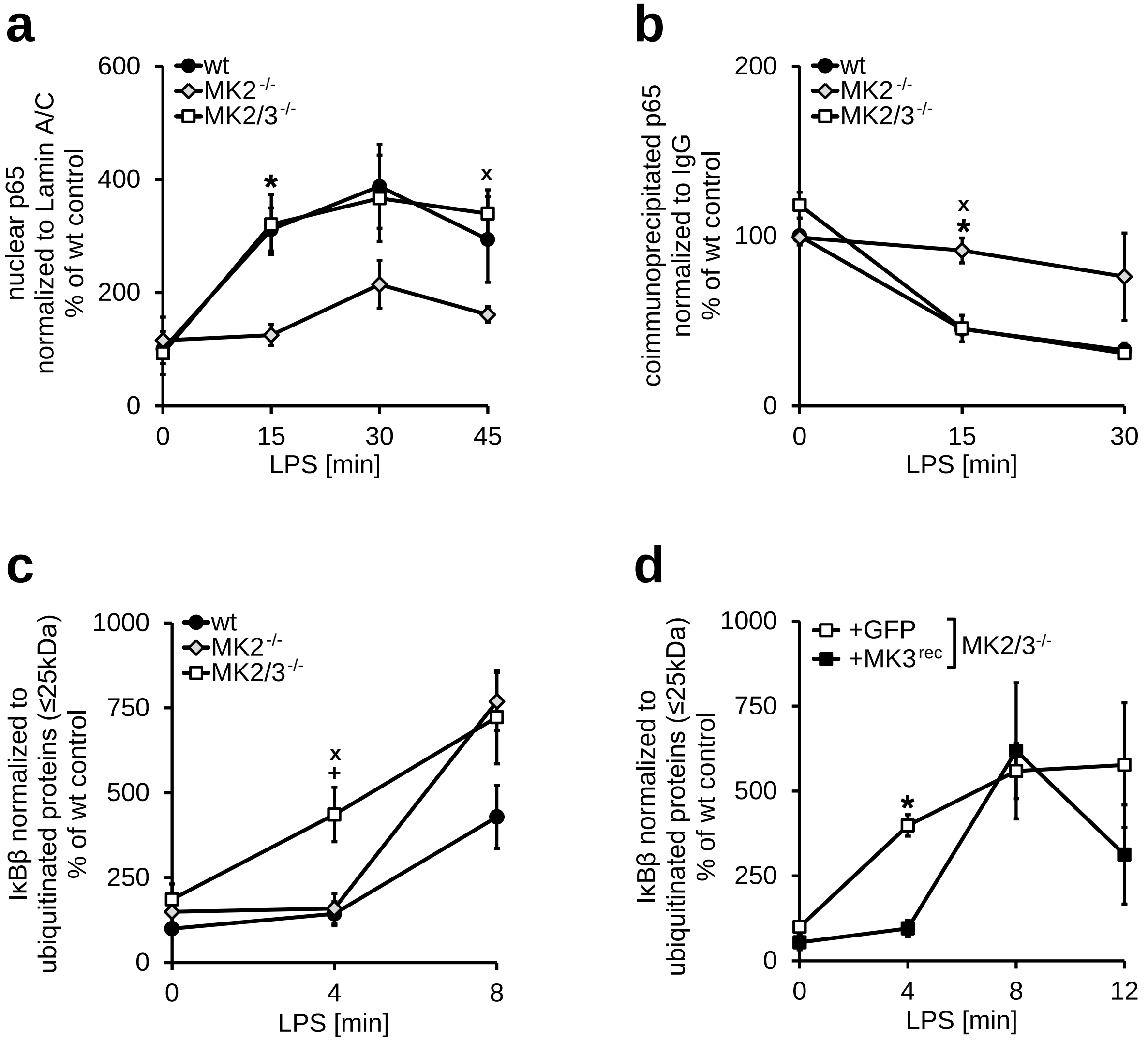

supplemental figure S6

a

p65

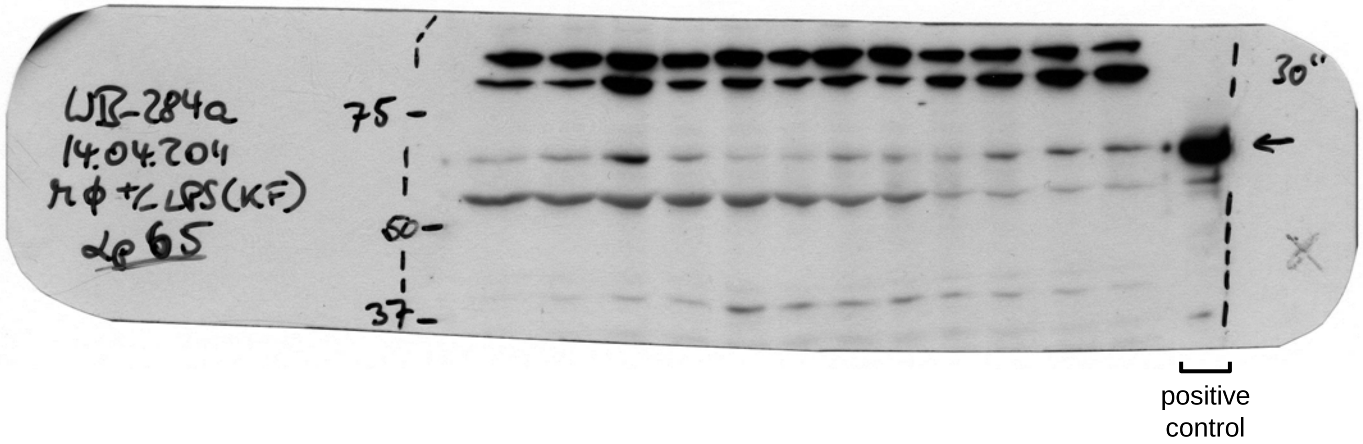

Lamin A/C

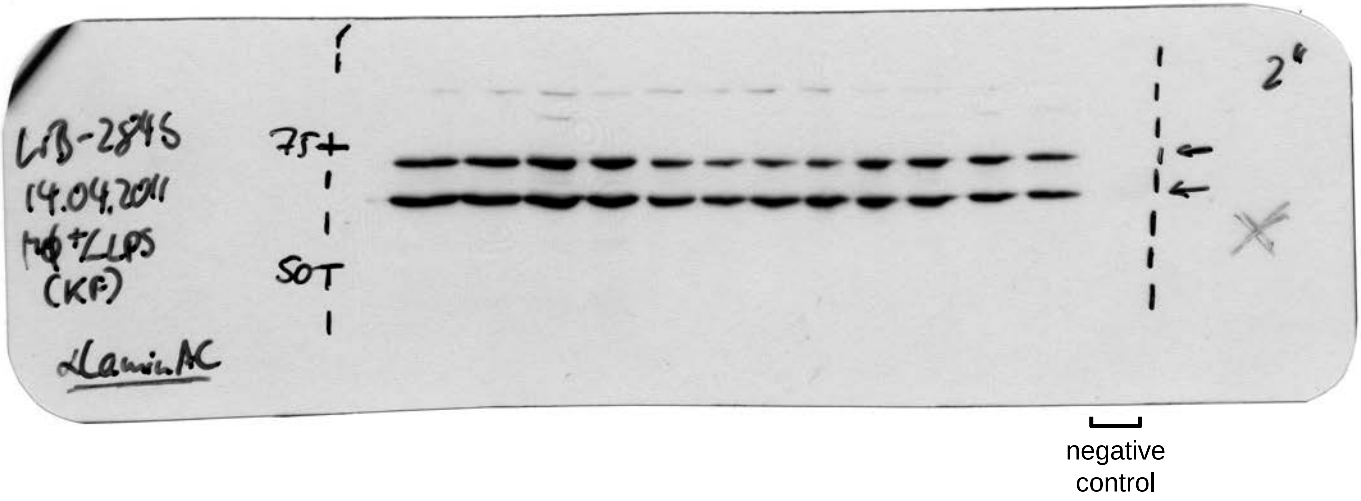

supplemental figure S7a

b

p65

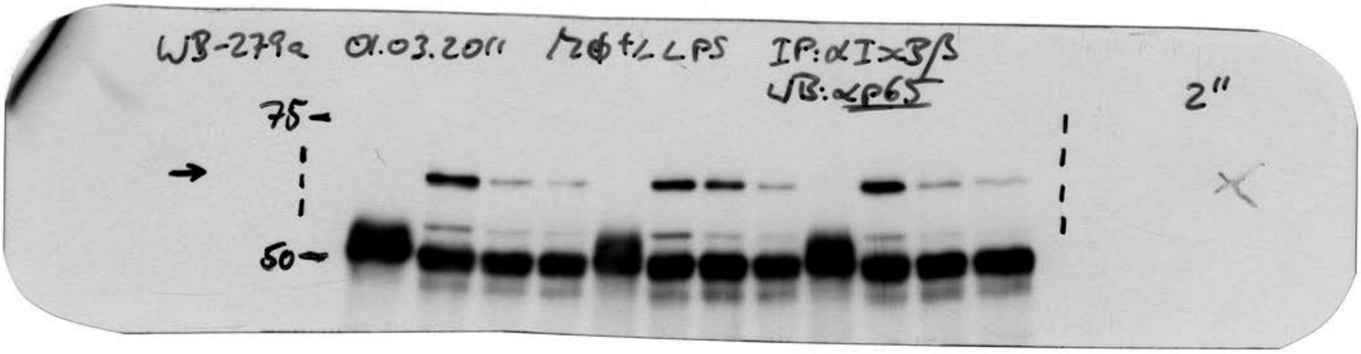

IgG and IκBβ

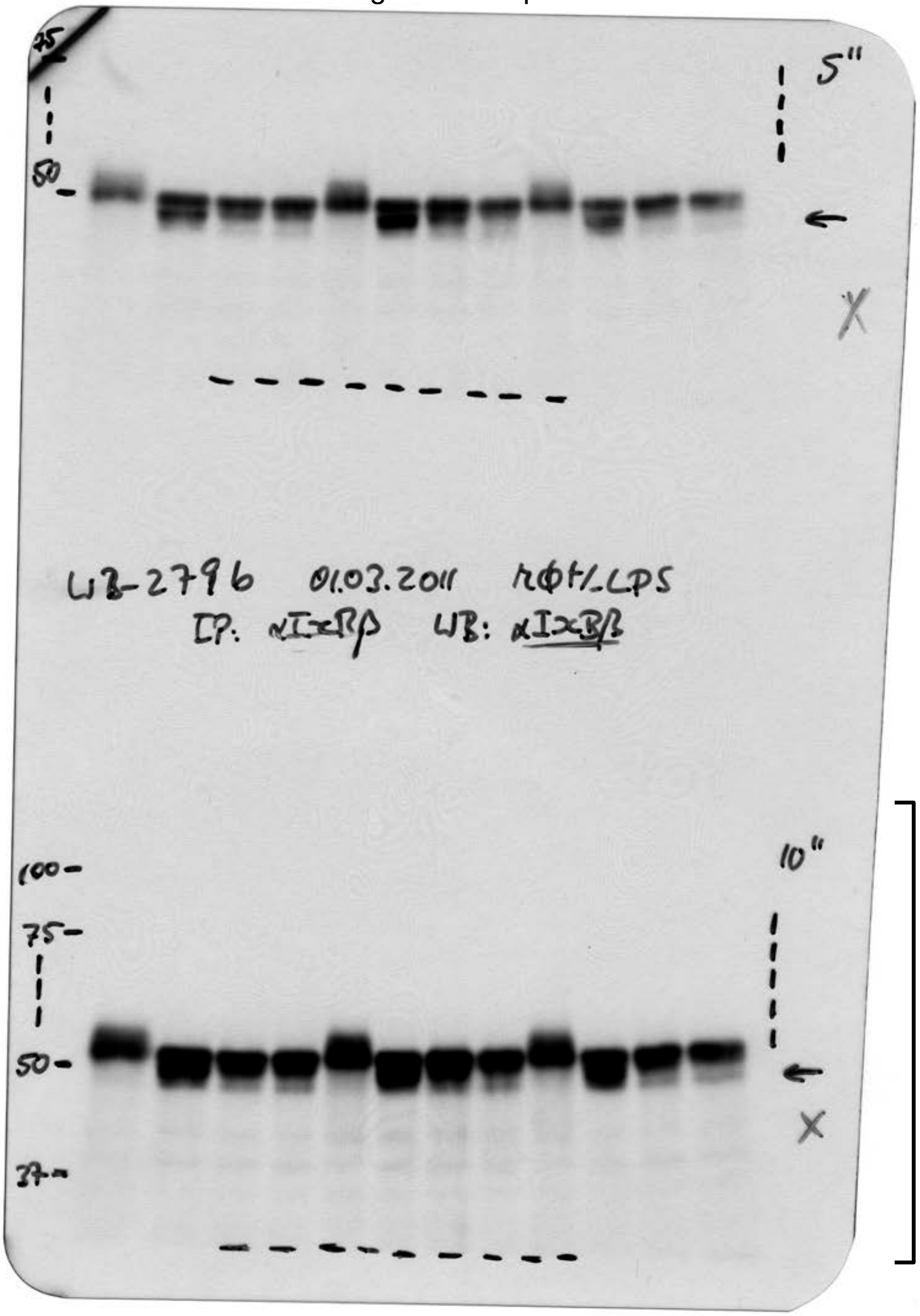

longer exposure time

supplemental figure S7b

c

P^{Ser}-IκBβ

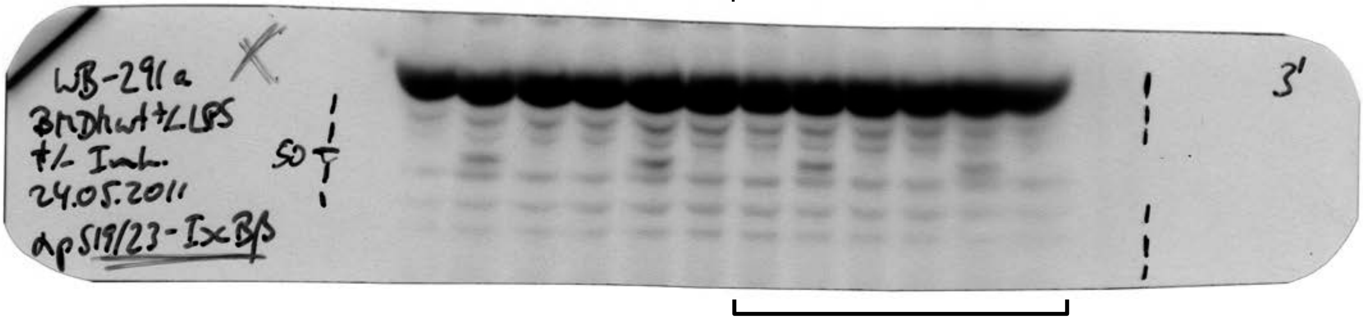

other samples

IκBβ

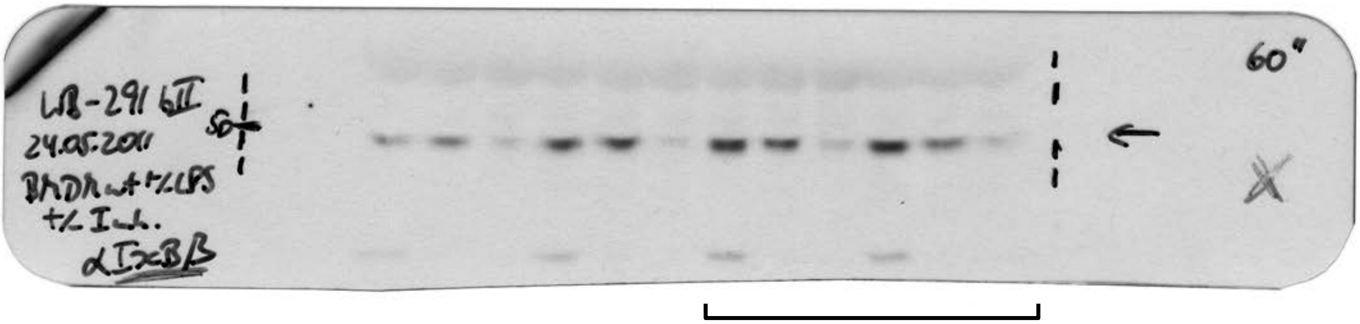

other samples

GAPDH

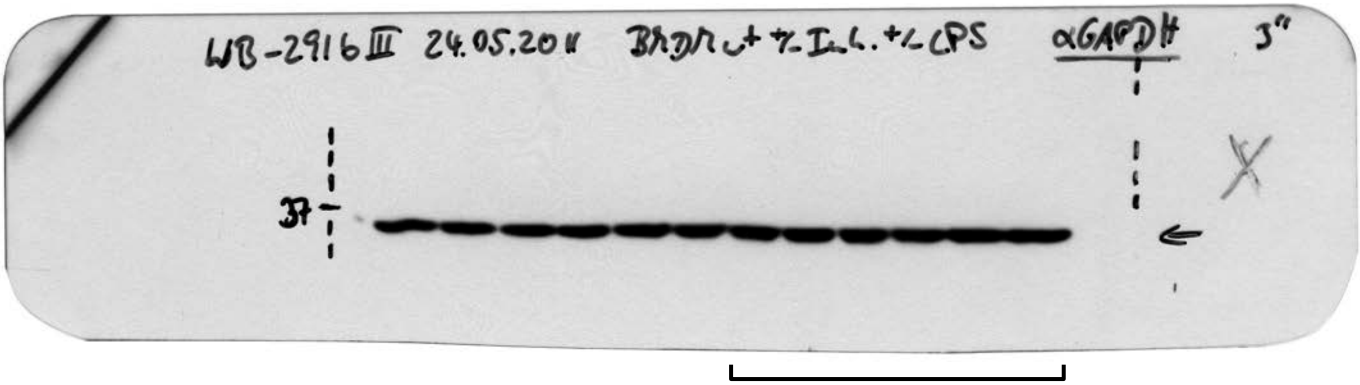

other samples

supplemental figure S7c

d

P^{Ser}-IκBβ

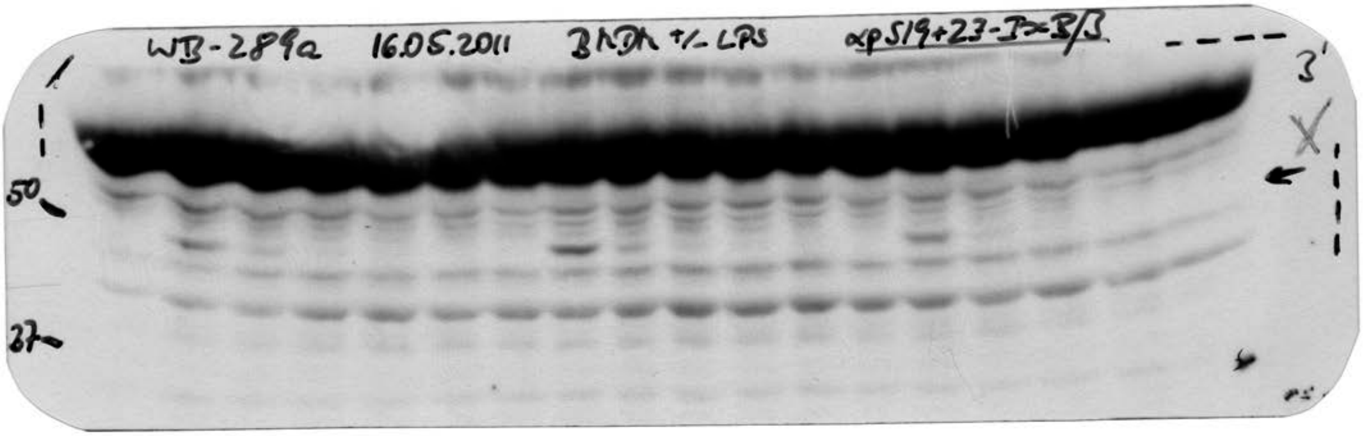

IκBβ

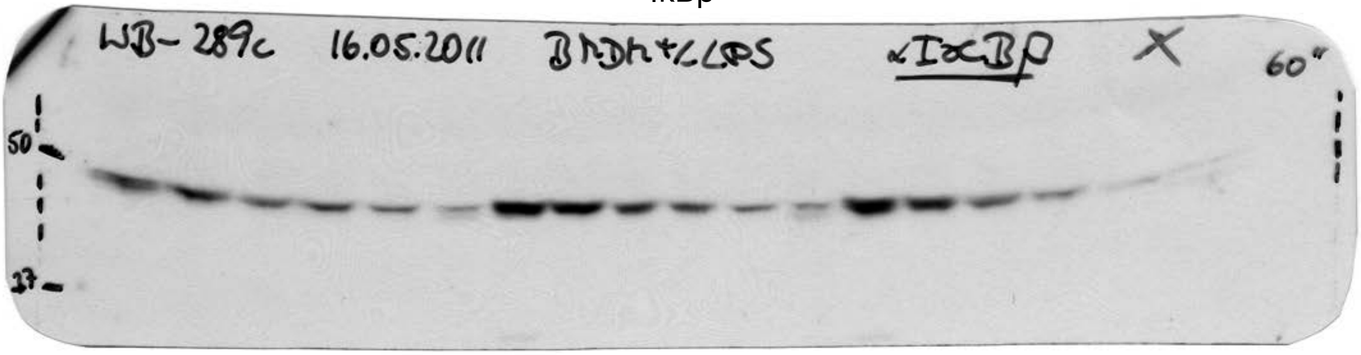

MK2

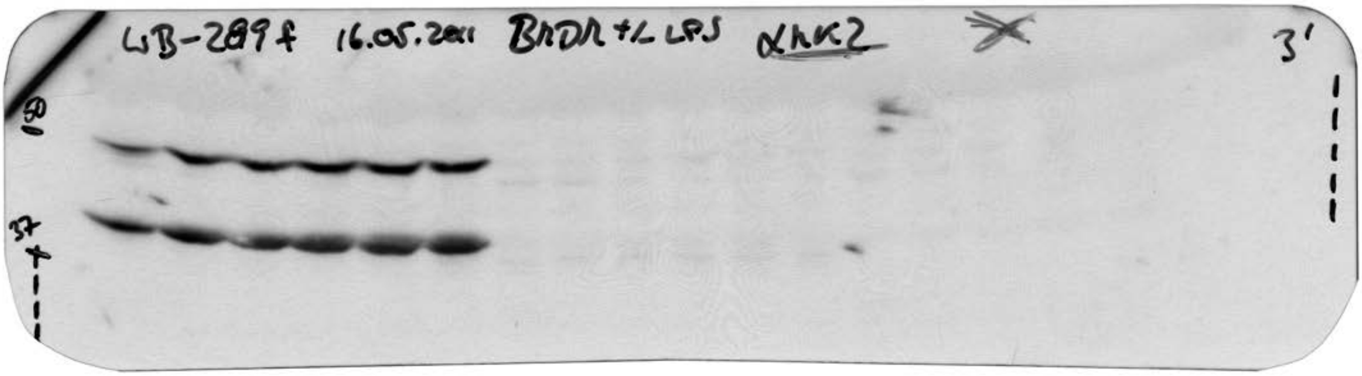

supplemental figure S7d

d

MK3

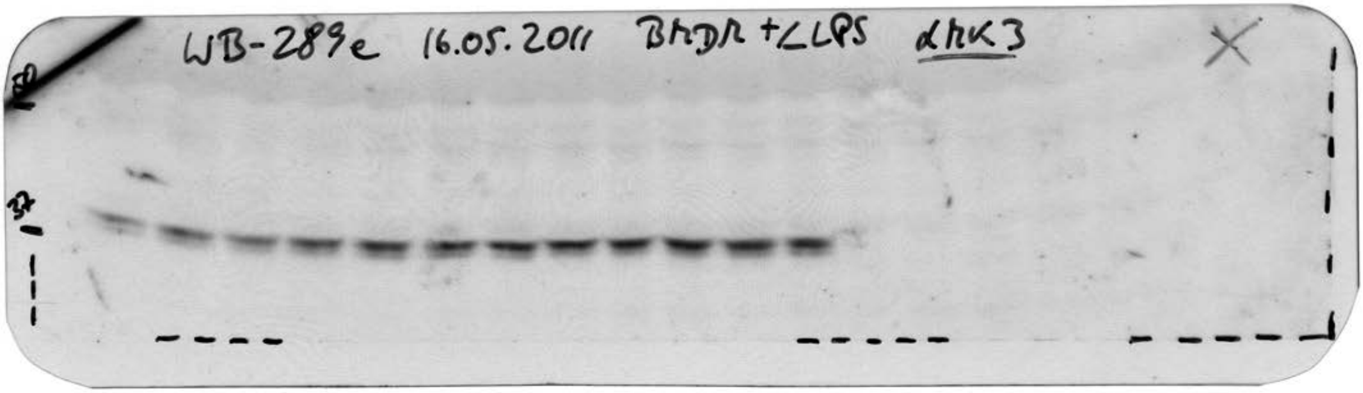

GAPDH

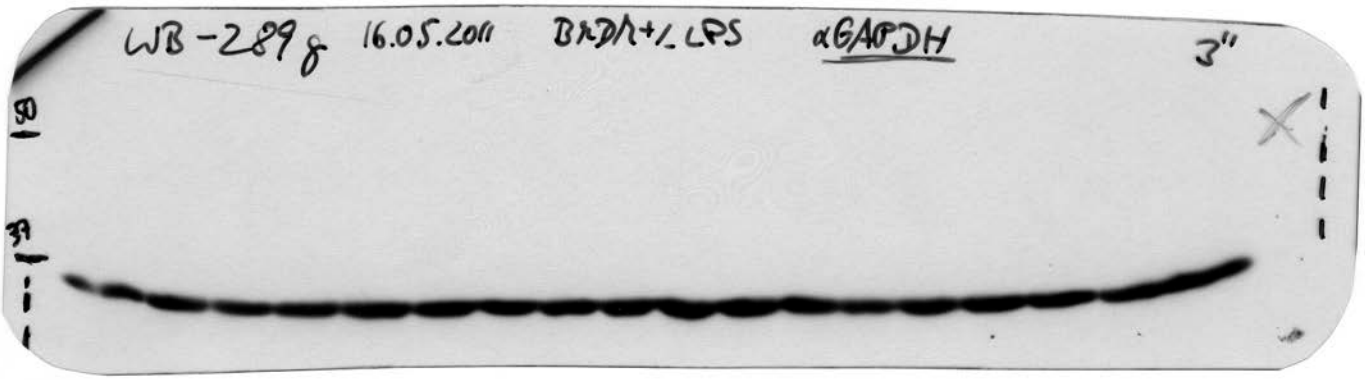

supplemental figure S7d

e

IκBβ

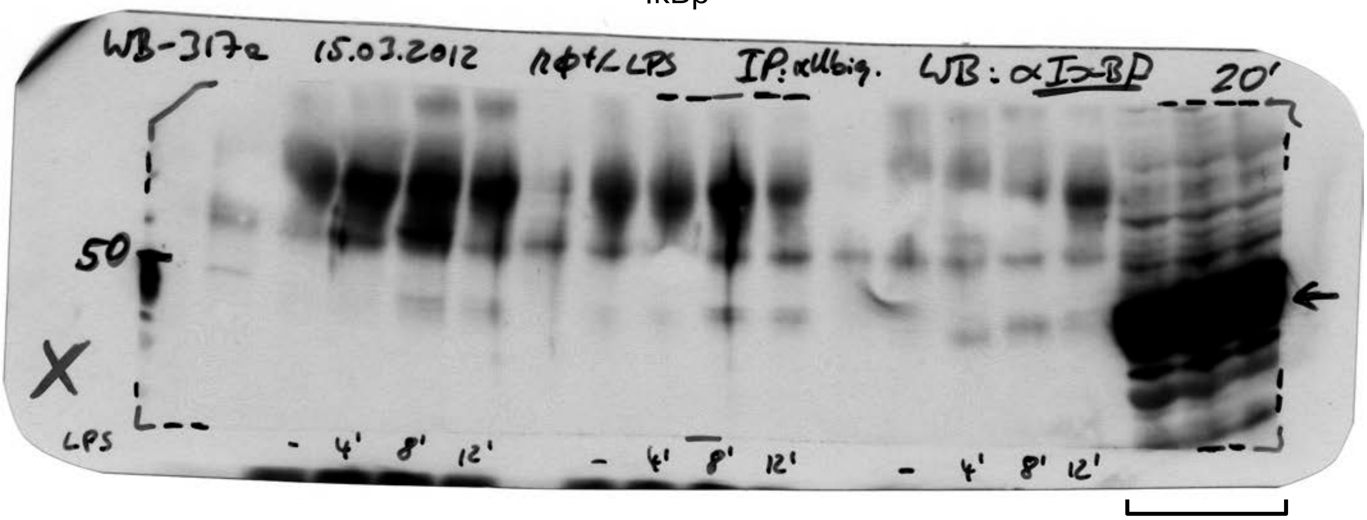

positive controls

ubiquitinated proteins ≤25kDa

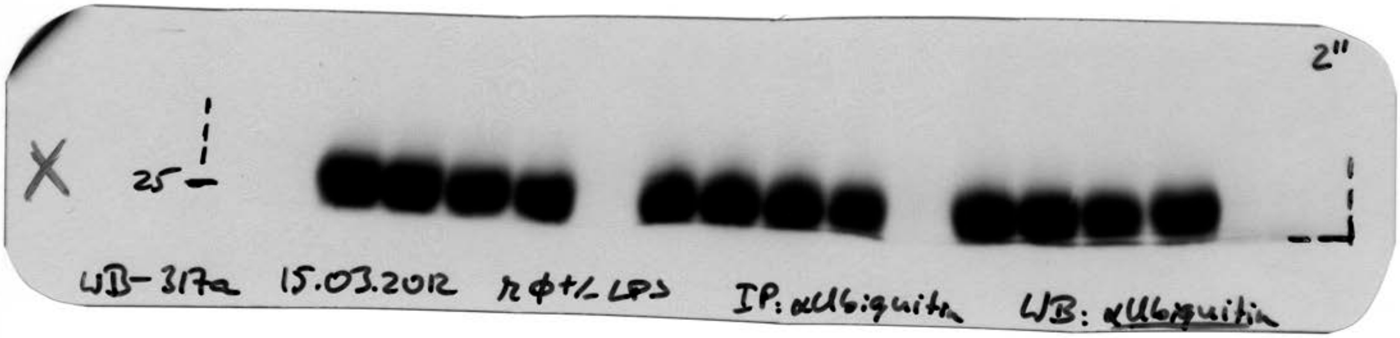

supplemental figure S7e

f

IκBβ

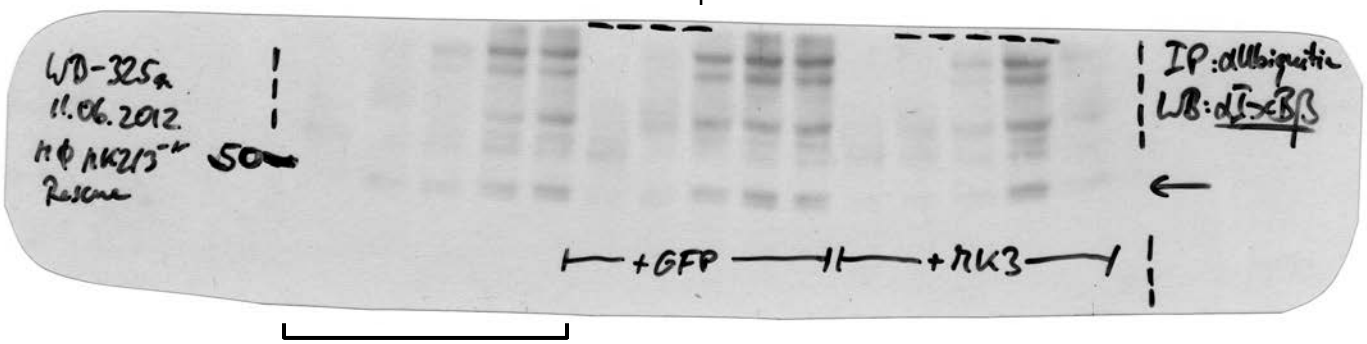

other samples

ubiquitinated proteins ≤25kDa

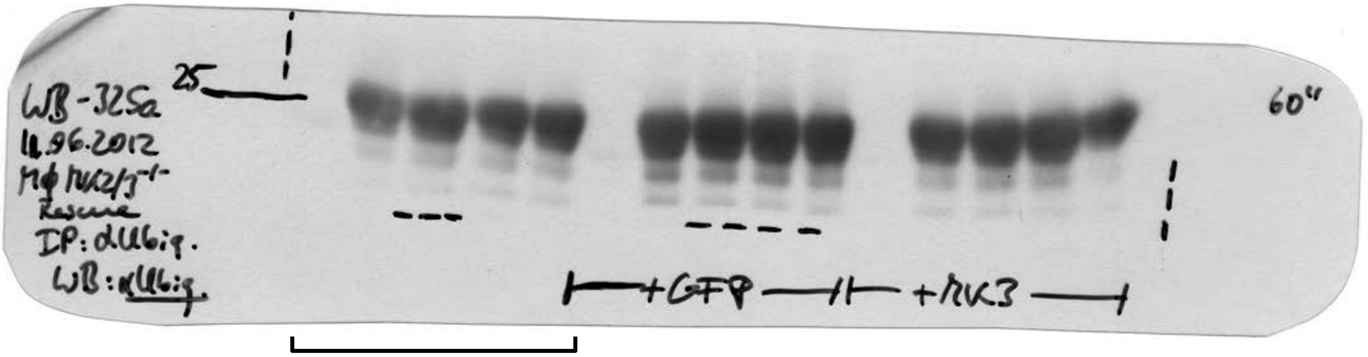

other samples

supplemental figure S7f

Target gene ID	Forward (5' to 3')	Reverse (5' to 3')
C1ra	CAGGAGAGCTGAGGACCCA	CTGAGGGAGGTAAATGGAGCC
Cxcl2	TGCCAAGGGTTGACTTCAAGA	AAGGCAAACTTTTTGACCGC
Cxcl3	CCAGACAGAAGTCATAGCCAC	CTTCATCATGGTGAGGGGCTT
Cxcl9	TCATCTTCCTGGAGCAGTGTG	GATTTGTAGTGGATCGTGCCT
Hdc	AGCCCTGTGAATACCGTGAA	AGGTATCCAGGCTGCACATT
Ifnb1	CCCTATGGAGATGACGGAGA	ACCCAGTGCTGGAGAAATTG
Il4ra	ACAGGCTGATGTTCTTCGAGT	ATCTGTCTGATTGGACCGGC
Il10	CCAAGCCTTATCGGAAATGA	TCCTGAGGGTCTTCAGCTTC
Il12b	TCTCCTGGTTTGCCATCGTT	GGGAGTCCAGTCCACCTCTA
Il19	GTCTGATTTCTGTGGACATGCG	CAGGGACAGGATGGTGACAT
Nod1	AGCTGGAGGATGCTTACGTG	TGGGTATACCTGCTTACTGGGT
Sdha	TGGGGAGTGCCGTGGTGTCA	GTGCCGTCCCCTGTGCTGGT
Serpina3f	CATGTTCATCCTCCCGGACC	GGGCAGGCAGAGCTCATTTA
Ttp	TTTCCCCTTCTGCCTTCTCT	TGGTGCTGGGGGTAGTAGAC

supplemental table 1

SUPPLEMENTAL FIGURE LEGENDS

Supplemental figure S1 – **LPS-induced transcript expression of IL-10 and IFN-β is not impaired upon MK3 deficiency.** Bone marrow derived macrophages (BMDM) were prepared from wild-type (wt) and MK3-deficient ($MK3^{-/-}$) mice and treated with 100 ng/ml LPS for the indicated durations. Total mRNA was extracted and determined using rtPCR as specified in the methods section. Data are presented as means ± SEMs based on at least 5 replicates per condition. Statistics were calculated by the 2way ANOVA test together with the Bonferoni test, a p-value smaller than 0.05 was considered as significant.

Supplemental figure S2 – **LPS-responsive genes controlled by MK2 and/or MK2/3 and observed by a whole genome array.** From the 24,288 genes analyzed in this whole genome array those with minimally 4-fold expression under wild-type conditions in BMDM and with at least one dependency towards MK2 or MK2/3 were studied after 2 (a,c,e) or 6 h (b,d,f) of treatment with 100 ng/ml LPS. In a and b the LPS-induced expression levels of the respective genes in one genotype in relation to the other genotype are shown in percent, the corresponding p-values are shown in c and d. wt vs. $MK2^{-/-}$ indicates the level under wild-type conditions when compared to MK2-deficient conditions. wt vs. $MK2/3^{-/-}$ indicates the level under wild-type conditions when compared to MK2/3-double-deficient conditions. $MK2^{-/-}$ vs. $MK2/3^{-/-}$ indicates the level under MK2-deficient conditions when compared to MK2/3-double-deficient conditions. Therefore, in a and b the percental rates for each gene reflect the degree of the difference between the expression levels of the respective genotypes. Rates higher than 100% indicate enhanced expression levels from the first specified genotype compared (vs.) to the second genotype, whereas rates lower than 100% indicate decreased expression levels when the first genotype is compared to the second specified genotype and a rate of 100% indicates no differences. As shown in c and d, at least one of the compared expression levels shows a significant difference with a p-value of 0.05 or lower ($p \leq 0.05$). A p-value higher than 0.05 was marked in red and was considered to be not significant. In e and f the values indicate the percentaged mean expression levels of the respective gene in different genotypes with or without LPS treatment. The values are specified in

relation to the wild-type control, which was set as 100%. For g to l the expression levels of the respective genes, also presented in e and f, were additionally shown as bar charts in relation to the wild-type control.

Supplemental figure S3 – **LPS-responsive genes controlled by MK2 and/or MK2/3 with at least 20% difference in the expression level upon comparison of wild-type, MK2$^{-/-}$ and MK2/3$^{-/-}$ BMDM.** All of the genes listed in supplemental figure 2 were further analyzed regarding their magnitude of differential gene expression upon LPS treatment after the comparison of two genotypes (wt vs. MK2$^{-/-}$, wt vs. MK2/3$^{-/-}$, MK2$^{-/-}$ vs. MK2/3$^{-/-}$). A threshold of 20% in difference was considered to be relevant. A minimum of 20% enhanced gene expression in the first specified genotype compared (vs.) to the second genotype was marked in red color, whereas a minimum of 20% reduced gene expression in the first specified genotype compared to the second genotype was marked in blue color. Differences in the relative gene expression smaller than 20% were considered to be not relevant and marked in white.

Supplemental figure S4 – **Allocation of the MK2/3-dependent genes in 6 different patterns of regulation.** The six different patterns of regulation dependent on MK2 and MK3 are suggested in figure 3a of the main text and indicated here (a and b) in the upper row as groups I-VI. According to the characteristics of each group mentioned in the main text and according to the analyses shown in supplemental figure 3 all of the genes were allocated to the six distinct groups and listed in a (2h LPS) and b (6h LPS).

Supplemental figure S5 – **LPS-induced transcript expression of target gene representatives of the 6 different types of regulation is not impaired upon MK3 deficiency.** Bone marrow derived macrophages (BMDM) were prepared from wild-type (wt) and MK3-deficient (MK3$^{-/-}$) mice and treated with 100 ng/ml LPS for the indicated durations. Total mRNA was extracted and determined using rtPCR as specified in the methods section. Data are presented as means ± SEM based on at least

4 replicates per condition. Statistics were calculated by the 2way ANOVA test together with the Bonferoni test, a p-value smaller than 0.05 was considered as significant.

Supplemental figure S6 – **MK3 counteracts p65 NFκB nuclear translocation in the absence of MK2 by delaying ubiquitination of the inhibitor of κB (IκB) β - graphics of densitometric analyses.** Densitometric analyses of the experiments shown in figure 6a,b,g and h were performed with n≥3 replicates. In a the mean signal intensity of p65 was normalized to lamin A/C, whereas in b it was normailzed to IgG of the immunoprecipitation antibody. In c and d the mean signal intensity of IκBβ was normalized to the fraction of ubiquitinated proteins with a maximum molecular weight of 25 kDa. Statistics were calculated using the unpaired t-test and a p-value of p≤0.05 was considered to be significant. Therefore, significant differences between wild-type and $MK2^{-/-}$ cells (*), wild-type and $MK2/3^{-/-}$ cells ($^+$) or $MK2^{-/-}$ and $MK2/3^{-/-}$ cells (^{x}) were marked.

Supplemental figure S7 – **MK3 counteracts p65 NFκB nuclear translocation in the absence of MK2 by delaying ubiquitination of the inhibitor of κB (IκB) β - uncropped scans of developed films.** Scans of the original developed films presented in figure 6 as cropped images of the respective immunoblots. Therfor, figure S7a shows the scans of figure 6a, figure S7b shows the scans of figure 6b, figure S7c shows the scans of figure 6c, figure S7d shows the scans of figure 6d, figure S7e shows the scans of figure 6g and figure S7f shows scans of figure 6h. For further details concerning the immunoblot detection and film development see methods section with the subsection “Equipment and settings for the detection of immunoblotted proteins”. Each scan is entitled with the antibody used for detection of the respective protein. Within the scans the molecular weight marker is marked on the left next to the detected signals, numbers reflect the molecular size in kDa. The edges of the respective immunoblot membrane are marked with dashed lines. In S7a on the right side a sample from a cytosolic lysate of untreated wt BMDM is shown, which is a positive control for p65 detection and a negative control for lamin A/C detection. In S7b the lower part of the IgG and IκBβ detection shows a longer exposure of the same immunoblot. In S7c and S7f, besides the samples shown in figure 6c and

6h, also other samples are shown and marked with a bracket. These samples were also placed on the same gel, but from a different experiment.

Supplemental table 1 – **Nucleotide sequences of the oligonucleotides used as primers for rtPCR in this study.**
